# Exploring regulatory networks of miR-96 in the developing inner ear

**DOI:** 10.1038/srep23363

**Published:** 2016-03-18

**Authors:** Morag A. Lewis, Annalisa Buniello, Jennifer M. Hilton, Fei Zhu, William I. Zhang, Stephanie Evans, Stijn van Dongen, Anton J. Enright, Karen P. Steel

**Affiliations:** 1 Wolfson Centre for Age-Related Diseases, King’s College London, Guy’s Campus, London SE1 1UL, UK; 2 Wellcome Trust Sanger Institute, Hinxton, Cambridge, CB10 1SA, UK; 3 EMBL EBI, Hinxton, Cambridge CB10 1SD.

## Abstract

Mutations in the microRNA *Mir96* cause deafness in mice and humans. In the diminuendo mouse, which carries a single base pair change in the seed region of miR-96, the sensory hair cells crucial for hearing fail to develop fully and retain immature characteristics, suggesting that miR-96 is important for coordinating hair cell maturation. Our previous transcriptional analyses show that many genes are misregulated in the diminuendo inner ear and we report here further misregulated genes. We have chosen three complementary approaches to explore potential networks controlled by miR-96 using these transcriptional data. Firstly, we used regulatory interactions manually curated from the literature to construct a regulatory network incorporating our transcriptional data. Secondly, we built a protein-protein interaction network using the InnateDB database. Thirdly, gene set enrichment analysis was used to identify gene sets in which the misregulated genes are enriched. We have identified several candidates for mediating some of the expression changes caused by the diminuendo mutation, including *Fos*, *Myc*, *Trp53* and *Nr3c1*, and confirmed our prediction that *Fos* is downregulated in diminuendo homozygotes. Understanding the pathways regulated by miR-96 could lead to potential therapeutic targets for treating hearing loss due to perturbation of any component of the network.

The diminuendo mouse carries a single base-pair change in the seed region of the microRNA gene *Mir96*. Heterozygotes exhibit rapidly progressive deafness, hair cell degeneration and stereocilia disorganisation, while homozygotes have a vestibular phenotype in addition to deafness and have lost their outer hair cells and most inner hair cells by four weeks of age. Microarrays carried out on diminuendo homozygotes (*Dmdo*/*Dmdo*) and wildtype littermates at four days old (P4), before the hair cell degeneration commences, found 96 transcripts to be significantly affected and, on a broader scale, a transcriptome-wide signature of misregulation of the microRNA^1^. Mutations in *MIR96* have also been reported to cause dominant progressive deafness in humans^2^,^3^. miR-96 is one of three microRNAs clustered together on chromosome 6; the others are miR-182 and miR-183. All three are expressed in multiple sensory organs, including the olfactory epithelium, vomeronasal organ, olfactory bulb, pineal body, tongue papilla and dorsal root ganglia^4^ in addition to sensory hair cells^5^.

miR-96 appears to be a master regulator of hair cell differentiation. Diminuendo homozygote hair cells fail to develop the potassium currents, synaptic machinery and connections typical of a mature hair cell, retaining juvenile characteristics^6^. However, the mechanisms by which the diminuendo mutation causes the failure of hair cell differentiation remain unclear. Several of the genes downregulated in *Dmdo/Dmdo* mice at P4, including *Ptprq*, *Gfi1*, *Kcna10* and *Slc26a5* (prestin), cause deafness when mutated^7^,^8^,^9^,^10^ and some of the unique characteristics of mice null for these genes are replicated in the diminuendo mouse, such as the short outer hair cells of prestin mutants^6^,^9^, and the delay in hair cell stereocilia differentiation seen in Ptprq functional nulls^11^. However, none of these genes can entirely account for the diminuendo phenotype, nor are any of them direct targets of miR-96; rather, the transcriptome-wide effects of the diminuendo mutation suggest it is regulating many genes through multiple pathways.

Regulatory network construction and analysis is a complex problem with no one easy solution. However, knowing both the starting point (the mutant miR-96) and the end points (the misregulated transcripts) means a set of hypothetical pathways can be drawn up, examined for consistency and tested *in vivo*. Here we present a regulatory network for miR-96 based on our existing data, our new expression data and on reports in the literature, and the results of testing some of its predictions. We have also constructed a protein-protein interaction network based on microarray data together with known and predicted targets of miR-96, and carried out gene set enrichment analyses on the microarray data. Our results demonstrate the differences between the methods, and also identify several genes highlighted as important in more than one analysis, which are candidates for therapeutic intervention.

## Results

### Microarray on newborn diminuendo homozygotes shows similar broad effects to that at P4

**Figure 1:**
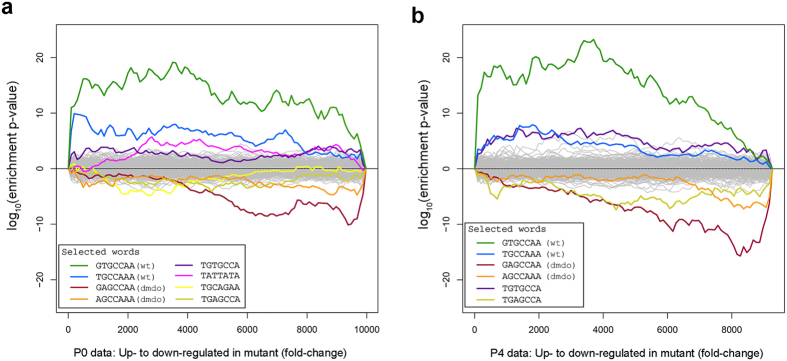
Sylamer analysis of the P0 (**a**) and P4 (**b**) microarrays showing enrichment and depletion of heptamers in 3′UTRs. The x-axis represents the sorted gene list from most upregulated at the left to most downregulated at the right. The hypergeometric significance for enrichment or depletion of each heptamer is measured on the y-axis. In both datasets the heptamers complementary to the wildtype miR-96 seed region (blue, green, marked with “wt”) are markedly enriched in upregulated genes and heptamers complementary to the mutant seed region (red, orange, marked with “dmdo”) are enriched in downregulated genes.

Because the diminuendo mutation appears to delay development, we carried out a microarray on the organ of Corti of newborn homozygote diminuendo and wildtype mice to examine the transcriptome at a younger age than our previous P4 analysis. We found fewer genes significantly affected at P0, but of the 18 that were, 17 showed the same direction of regulation in the P4 microarray (Supplementary Fig. S1, Supplementary Table S1). We then carried out a Sylamer analysis to examine the direct effects of the mutant microRNA on the transcriptome. Sylamer analyses examine all possible heptamers in the 3′UTR of genes in the microarray sorted by expression value and detect whether any heptamers are significantly more common in over- or under-expressed genes than would be expected by chance^12^. In this case, the heptamer complementary to the wildtype miR-96 seed was enriched in upregulated genes and the heptamer complementary to the mutant miR-96 seed was enriched in downregulated genes in the mutant sample. This is very similar to the results of the P4 microarray^1^ and shows that the mutant microRNA is having a broad effect on many genes at this earlier age (Fig. 1).

### Testing microarray and network genes

**Figure 2:**
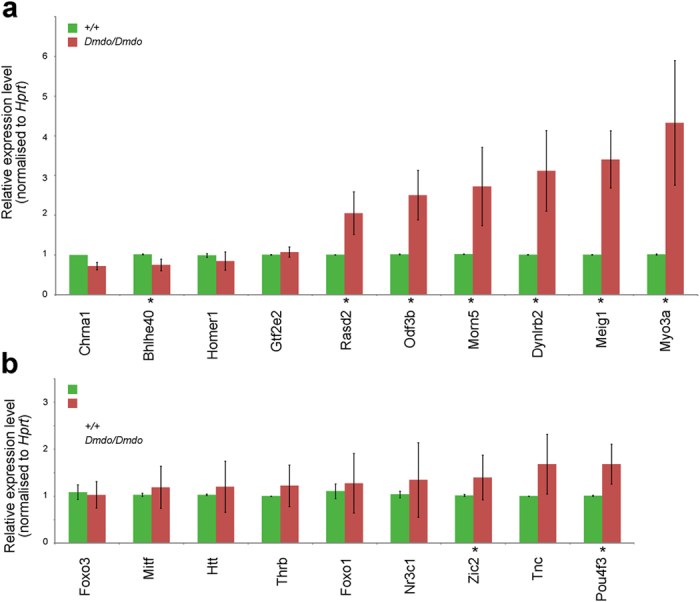
Testing gene expression in diminuendo homozygotes. qRTPCR was carried out on cDNA from P4 organs of Corti in wildtype (green) and diminuendo homozygote (red) littermates to test gene expression changes. Error bars are standard deviation (*adjusted P < 0.05). (**a**) Confirmation of misregulation of gene expression from the P4 microarray (*Chrna1*: wildtype n = 3, mean = 1.00 ± 0.00(s.d.); homozygote n = 3, mean = 0.72 ± 0.10(s.d.) *Gtf2e2*: wildtype n = 5, mean = 1.01 ± 0.01(s.d.); homozygote n = 5, mean = 1.08 ± 0.13(s.d.) *Rasd2*: wildtype n = 5, mean = 1.01 ± 0.01(s.d.); homozygote n = 5, mean 2.06 ± 0.54(s.d.) *Odf3b*: wildtype n = 5, mean = 1.02 ± 0.01(s.d.); homozygote n = 5, mean = 2.51 ± 0.63(s.d.) *Dynlrb2*: wildtype n = 5, mean = 1.01 ± 0.01(s.d.); homozygote n = 5, mean = 3.12 ± 1.02(s.d.) *Meig1*: wildtype n = 5, mean = 1.01 ± 0.01(s.d.); homozygote n = 5, mean = 3.41 ± 0.72(s.d.) *Bhlhe40*: wildtype n = 6, mean = 1.02 ± 0.01(s.d.); homozygote n = 6, mean = 0.75 ± 0.15 (s.d.) *Homer1*: wildtype n = 6, mean = 0.99 ± 0.05(s.d.); homozygote n = 6, mean = 0.85 ± 0.23(s.d.) *Morn5*: wildtype n = 6, mean = 1.02 ± 0.01(s.d.); homozygote n = 6, mean = 2.72 ± 0.99(s.d.) *Myo3a*: wildtype n = 6, mean = 1.01 ± 0.02(s.d.); homozygote n = 6, mean = 4.33 ± 1.57(s.d.) Wilcoxon t-test: *Chrna1*: p = 0.28; *Gtf2e2*: p = 0.84; *Rasd2*: p = 0.03; *Odf3b*: p = 0.03; *Dynlrb2*: p = 0.03; *Meig1*: p = 0.03; *Bhlhe40*: p = 0.02; *Homer1*: p = 0.28; *Morn5*: p = 0.02; *Myo3a*: p = 0.02. (**b**) Testing targets and intermediate genes suggested by the literature. *Foxo3*: wildtype n = 5, mean = 1.09 ± 0.16(s.d.); homozygote n = 5, mean = 1.03 ± 0.28(s.d.) *Mitf*: wildtype n = 6, mean = 1.03 ± 0.04(s.d.); homozygote n = 6, mean = 1.19 ± 0.45(s.d.) *Htt*: wildtype n = 5, mean = 1.03 ± 0.02(s.d.); homozygote n = 5, mean = 1.20 ± 0.54(s.d.) *Thrb*: wildtype n = 5, mean = 1.00 ± 0.00(s.d.); homozygote n = 5, mean = 1.22 ± 0.44(s.d.) *Foxo1*: wildtype n = 5, mean = 1.11 ± 0.16(s.d.); homozygote n = 5, mean = 1.28 ± 0.63(s.d.) *Nr3c1*: wildtype n = 5, mean = 1.04 ± 0.07(s.d.); homozygote n = 5, mean = 1.35 ± 0.80(s.d.) *Zic2*: wildtype n = 5, mean = 1.02 ± 0.02(s.d.); homozygote n = 5, mean = 1.40 ± 0.48(s.d.) *Tnc*: wildtype n = 5, mean = 1.00 ± 0.00(s.d.); homozygote n = 5, mean = 1.68 ± 0.64(s.d.) *Pou4f3*: wildtype n = 5, mean = 1.01 ± 0.01(s.d.); homozygote n = 5, mean = 1.68 ± 0.43(s.d.). Wilcoxon t-test: *Foxo3*: p = 0.28; *Mitf*: p = 0.63; *Htt*: p = 0.84; *Thrb*: p = 0.28; *Foxo1*: p = 1; *Nr3c1*: p = 0.95; *Zic2*: p = 0.03; *Tnc*: p = 0.28; *Pou4f3*: p = 0.03.

We had previously confirmed the misregulation of 15 genes from the P4 microarray by qRTPCR^1^), but prior to building the network, 10 more genes were tested and the misregulation was confirmed in 7 of them (Fig. 2a; *Bhlhe40, Rasd2, Odf3b, Mom5, Dynlbr2, Meig1, Myo3a*). We also tested the potential intermediate regulators *Nr3c1*, *Foxo1*, *Foxo3* and *Mitf*, which are all confirmed targets of miR-96 in different tissues^13^,^14^,^15^,^16^), *Pou4f3*, a known deafness gene which regulates *Gfi1*^17^, and *Thrb* which has been shown to regulate *Slc26a5* in the outer hair cells^18^. *Zic2* was identified previously as a target by miRanda prediction^1^, and preliminary networks created using Ingenuity IPA suggested *Htt* and *Tnc* as potential intermediate regulators. Of those nine potential intermediates, only expression of *Zic2* and *Pou4f3* were significantly affected when tested by qRTPCR (Fig. 2b, adjusted P < 0.05).

The misregulation of genes by mutant miR-96 can only be relevant if those genes are co-expressed with miR-96 in the hair cells, so we tested 7 of the misregulated genes by immunohistochemistry and confirmed that *Anxa4*, *Meig1*, *Homer1*, *Kcna10*, *Kalrn*, *Gtf2e2*, and *Tppp* were present in wildtype hair cells (Supplementary Fig. S2). *Nr3c1, Foxo3, Thrb* and *Pou4f3* are known to be expressed in the hair cells^19^,^20^,^21^,^22^, and *Foxo1* is expressed in the otic vesicle early in development^23^. The remaining 4 genes, *Mitf, Htt, Zic2* and *Tnc*, were tested by immunohistochemistry and all found to be expressed in wildtype hair cells (Supplementary Fig. S2). No difference in staining pattern or intensity was observed in *Dmdo/Dmdo* homozygote mutant hair cells.

### Identifying direct targets of miR-96 using an Ago2 pulldown assay

Identifying the targets of miR-96 is critical for exploring the networks it controls. We looked for direct targets of miR-96 by immunoprecipitating Ago2 (one of the RISC complex proteins to which microRNAs bind and direct to their targets) and extracting the RNA bound to it^24^ using RNA from the olfactory bulbs and organs of Corti of six *Dmdo/Dmdo* homozygotes and six sex-matched wildtype littermates at P4, and analysed the RNA by microarray. We used the olfactory bulb as well as the organ of Corti because miR-96 is known to be expressed there^4^ and it offers more material than the organ of Corti.

**Figure 3:**
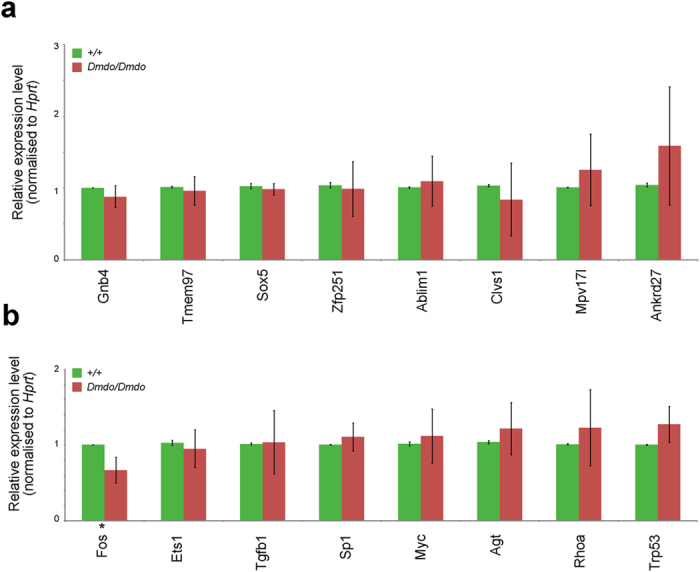
Testing predicted targets of miR-96 and important nodes from the regulatory network. qRTPCR was carried out on cDNA from P4 organs of Corti in wildtype (green) and diminuendo homozygote (red) littermates to test gene expression changes. Error bars are standard deviation (*adjusted P < 0.05). (**a**) Testing predicted targets of miR-96 from the Ago2 pulldown microarray. *Gnb4*: wildtype n = 5, mean = 1.00 ± 0.00(s.d.); homozygote n = 5, mean = 0.88 ± 0.15(s.d.) *Tmem97*: wildtype n = 5, mean = 1.01 ± 0.01(s.d.); homozygote n = 5, mean = 0.96 ± 0.20(s.d.) *Sox5*: wildtype n = 5, mean = 1.03 ± 0.04(s.d.); homozygote n = 5, mean = 0.98 ± 0.08(s.d.) *Zfp251*: wildtype n = 5, mean = 1.04 ± 0.04(s.d.); homozygote n = 5, mean = 0.99 ± 0.38(s.d.) *Ablim*: wildtype n = 5, mean = 1.01 ± 0.01(s.d.); homozygote n = 5, mean = 1.10 ± 0.35(s.d.) *Clvs1*: wildtype n = 3, mean = 1.03 ± 0.02(s.d.); homozygote n = 3, mean = 0.84 ± 0.51(s.d.) *Mpv17l*: wildtype n = 5, mean = 1.01 ± 0.01(s.d.); homozygote n = 5, mean = 1.25 ± 0.50(s.d.) *Ankrd27*: wildtype n = 5, mean = 1.04 ± 0.03(s.d.); homozygote n = 5, mean = 1.59 ± 0.82(s.d.). Wilcoxon t-test: *Gnb4*: p = 0.28; *Tmem97*: p = 0.84; *Sox5*: p = 0.95; *Zfp251*: p = 0.28; *Ablim*: p = 0.84; *Clvs1*: p = 0.84; *Mpv17l*: p = 0.84; *Ankrd27*: p = 0.28. (**b**) Testing important nodes from the regulatory network. *Fos*: wildtype n = 6, mean = 1.00 ± 0.00(s.d.); homozygote n = 6, mean = 0.67 ± 0.17(s.d.) *Ets1*: wildtype n = 6, mean = 1.03 ± 0.03(s.d.); homozygote n = 6, mean = 0.95 ± 0.25(s.d.) *Tgfb1*: wildtype n = 6, mean = 1.01 ± 0.01(s.d.); homozygote n = 6, mean = 1.04 ± 0.42(s.d.) *Sp1*: wildtype n = 6, mean = 1.00 ± 0.00(s.d.); homozygote n = 6, mean = 1.11 ± 0.18(s.d.) *Myc*: wildtype n = 6, mean = 1.02 ± 0.03(s.d.); homozygote n = 6, mean = 1.12 ± 0.36(s.d.) *Agt*: wildtype n = 6, mean = 1.04 ± 0.02(s.d.); homozygote n = 6, mean = 1.22 ± 0.34(s.d.) *Rhoa*: wildtype n = 6, mean = 1.01 ± 0.01(s.d.); homozygote n = 6, mean = 1.23 ± 0.50(s.d.) *Trp53*: wildtype n = 6, mean = 1.00 ± 0.00(s.d.); homozygote n = 6, mean = 1.27 ± 0.24(s.d.). Wilcoxon t-test: *Fos*: p = 0.02; *Ets1*: p = 0.84; *Tgfb1*: p = 1; *Sp1*: p = 0.63; *Myc*: p = 1; *Agt*: p = 0.63; *Rhoa*: p = 1; *Trp53*: p = 0.21.

There were no genes reported as significantly misregulated in mutants (adjusted P < 0.05) after p-value adjustment for multiple testing. However, 216 genes from the organ of Corti and 23 from the olfactory bulb were downregulated in mutant samples with an unadjusted P < 0.01, suggesting these were more strongly bound by the wildtype miR-96 seed region. We tested these genes against three microRNA target predictors: miRDB (http://mirdb.org/cgi-bin/search.cgi)^25^, starBase (http://starbase.sysu.edu.cn/index.php)^26^ and Diana-microT (http://diana.cslab.ece.ntua.gr/microT/)^27^. We also used our target list compiled previously using miRanda^1^ and did a text search for the presence of complementary matches to the miR-96 seed region in the 3′UTR (a less stringent prediction method) (Supplementary Table S2). From the resulting lists, 12 targets were selected based on the strength of their fluorescence in the microarray and on their bioinformatics predictions: *Ablim1*, *Unkl*, *Cacna1c*, *Akap7*, *Tmem97*, *Gnb4*, *Mpv17l*, *Clvs1*, *6820408C15Rik*, *Sox5*, *Ankrd27* and *Zfp251.* We checked for the presence of the 12 transcripts in wildtype organ of Corti cDNA by RTPCR and qRTPCR. *6820408C15Rik* was not detected, *Unkl*, *Cacna1c* and *Akap7* qRTPCR probes did not work reliably, suggesting a very low transcript level, and none of the remaining 8 genes were significantly upregulated in diminuendo mutant organ of Corti (Fig. 3a, adjusted P < 0.05), as would be expected of a target of miR-96. We hoped to identify multiple targets through the Ago2 pulldown, but it may be that the small amount of material limited the results.

### Compiling a list of direct targets of miR-96

We included several targets that have been reported in other studies: *Foxo1, Foxo3, Mitf*, *Adcy6*, *Clic5*, *Scarb1*, *Nr3c1, Scn3a*, *Spast*, *Gpc3*, *Insig2*, *Irs1*, *Slc1a1*, *Gpc1*, *Alk*, *Rad51*, *Rev1*, *Hbp1* and *Reck* (see Supplementary Table S3 for references). We also included *Aqp5*, *Celsr2* and *Odf2*, which were found to be targets by luciferase assay in our previous study^1^; *Hspa2*, *Pnpla8*, *Sdc2*, *Arf2*, *Casc1*, *Gad2* and *St8sia3*, which were upregulated in diminuendo homozygotes at P4^1^ and P0, and have complementary matches to the miR-96 seed region in their 3′ UTRs; *Zic2*, which is a predicted target and is upregulated at P4; and *Osbpl2*, a predicted target which is specifically expressed in the outer hair cell stereocilia^28^. We included all these genes in our list of direct targets of miR-96 (Supplementary Table S3).

### Compiling the misregulated gene list

We compiled a list of genes and associated expression values, consisting of all the misregulated genes from the P4 and P0 microarrays with adjusted P < 0.1, and those genes with expression changes detected by qRTPCR with adjusted P < 0.05 (Supplementary Table S4). The less stringent P value cutoff was chosen in order to maximise the genes available for network construction. We had several indicators that genes with adjusted P < 0.1 still offer biological relevance in this case. For example, *Dynlrb2* has an adjusted P = 0.0522 and was shown to be significantly upregulated by qRTPCR (Fig. 2a, adjusted P = 0.0004). *Sdc2* has adjusted P = 0.089 in the P0 microarray, but at P4 it has adjusted P = 0.009 and by qRTPCR, also at P4, it was shown to be upregulated with adjusted P = 0.026^1^. *Pnpla8* has adjusted P = 0.075 in the P4 microarray and adjusted P = 0.036 in the P0 microarray. In the case of the p0 microarray, there were 18 genes with adjusted P < 0.05, and raising the p-value cutoff to 0.1 meant including just 6 more. For the p4 microarray, raising the threshold to p < 0.1 meant 46 genes were added to the initial 85 with p < 0.05. If the microarray gave a significant result but qRTPCR testing did not, the microarray expression value was used where quantitative data was required. We also added genes identified by qRTPCR in our previous studies^1^,^6^. This misregulated gene list includes a few genes predicted to be direct targets, so there is some overlap between this list (Supplementary Table S4) and the list of direct targets (Supplementary Table S3).

### Construction of a regulatory network

**Figure 4:**
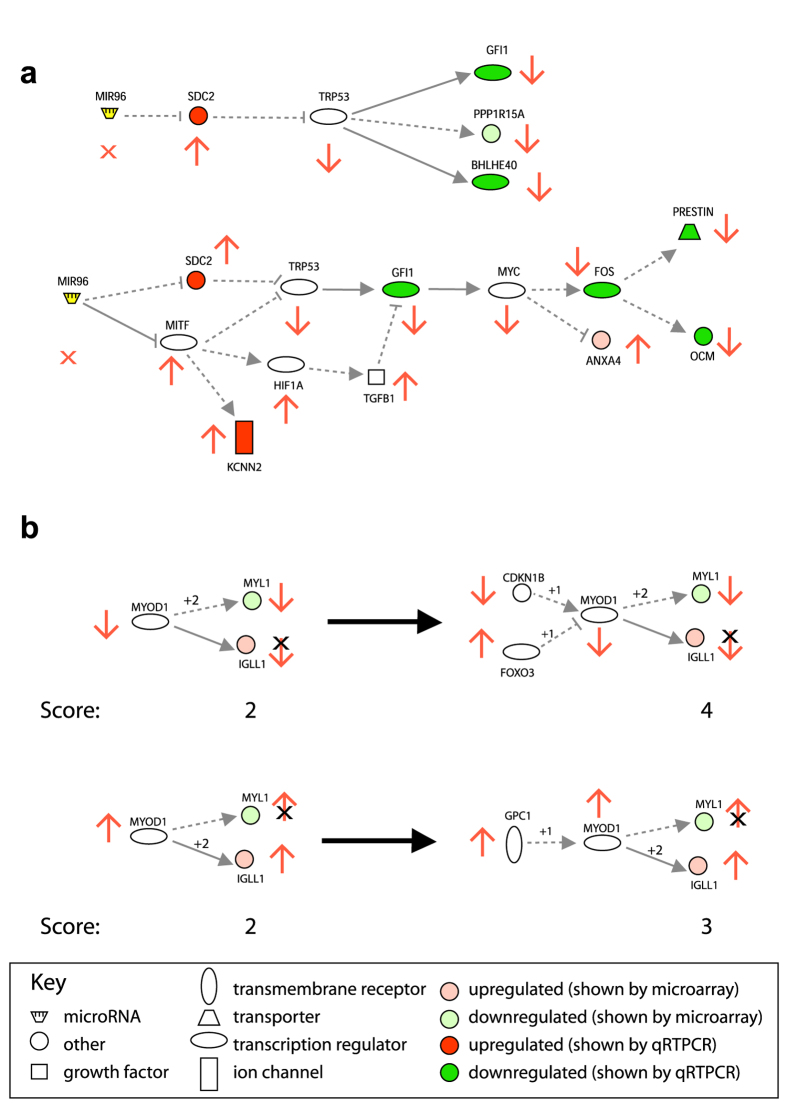
Pathways from the regulatory network construction process. Up- and downregulation is indicated by red and green colouration respectively. Pale green or pink means the node’s misregulation has been reported in the microarrays, and intense green or red means the node’s misregulation has been shown by qRTPCR. miR-96 is shown in yellow. Arrows indicate positive regulation (activation, increased transcription), and bars show negative regulation (repression, reduction in activity); solid lines indicate direct regulation (for example, direct binding of protein to promoter) and dotted lines indirect regulation (where other genes may mediate the observed effect of one gene upon another). **(a**) Two examples of simple pathways. (**b**) An example of the scoring system for Myod1. Genes directly downstream from Myod1 score 2, but that results in an equal prediction score for Myod1 being up- or downregulated (2; on the left). Once the regulating genes are taken into account (on the right), Myod1 scores 4 if it is downregulated but only 3 if it is upregulated, so it is predicted to be downregulated, and the inconsistent links between Myod1 and Igll1, and Gpc1 and Myod1, were removed from the network.

**Figure 5:**
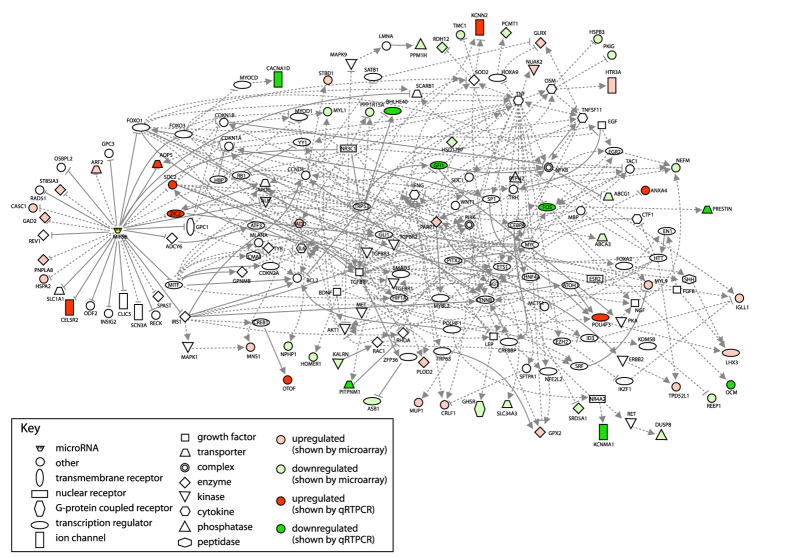
The Regulatory Network. The regulatory network constructed using regulatory interactions reported in the literature. Up- and downregulation is indicated by red and green colouration respectively. Pale green or pink means the node’s misregulation has been reported in the microarray, and intense green or red means the node’s misregulation has been shown by qRTPCR. miR-96 is shown in yellow. Arrows indicate positive regulation (activation, increased transcription), and bars show negative regulation (repression, reduction in activity); solid lines indicate direct regulation (for example, direct binding of protein to promoter) and dotted lines indirect regulation (where other genes may mediate the observed effect of one gene upon another).

To create this network, the miR-96 direct targets (Supplementary Table S3; 31 genes) were treated as one separate group and the rest of the misregulated gene list, excluding the predicted targets, were a second separate group (Supplementary Table S4; 188 genes). The two groups were first connected to each other using Ingenuity IPA. The software has the option to connect input genes using its own manually curated database; for the regulatory network we report here, only this database was used to connect the input genes, and only regulatory interactions were permitted. Not every misregulated gene could be accounted for within the network (most notably *Ptprq*, for which no upstream regulators have been described), but when no further genes could be connected, every link was checked against the original published article and incorrect links were removed (for example, many papers studying Htt carry out experiments using the mutant poly-CAG form, which is not relevant for this network). Each pathway within the network was checked for consistency with respect to the observed gene misregulation, and the intermediate genes along that pathway were annotated with a predicted change of expression/activity (Fig. 4a). Where intermediate genes could be up- or downregulated, and either way could explain a number of observed gene expression changes, a weighting algorithm was used to determine which prediction explained more changes, first taking into account known misregulation, then predicted misregulation, of genes both up- and downstream (Fig. 4b). The resulting network is internally consistent and offers multiple hypotheses for further testing (Fig. 5).

The majority of genes (47) have only one downstream connection. 18 genes have two downstream connections, twelve genes have three, and eight genes have four. The maximum number of downstream connections belongs to miR-96, which has 31 direct targets predicted or confirmed in the literature. In between the two extremes are fourteen genes with between 7 and 17 downstream connections: *Agt* (10), *Cebpa* (7)*, Sdc2* (7)*, Parp1* (8), *Irs1* (9)*, Foxo1* (10), *Foxo3* (9), *Htt* (8), *Mitf* (10), *Myc* (10), *Nr3c1* (14)*, Tgfb1* (17), *Tnf* (17) and *Trp53* (10). Of these, *Sdc2* and *Parp1* are known to be upregulated in diminuendo homozygotes, and only *Irs1* and *Foxo3* lack a direct connection to at least one gene with known misregulation (Fig. 5).

If these genes really do mediate the effects of miR-96, then they offer candidate therapeutic targets for modulating the network as a whole and ultimately the expression of many genes in the hair cells. *Trp53*, for example, regulates *Ppp1r15a*, *Bhlhe40* and *Gfi1* directly, and indirectly regulates *Slc26a5*, *Ocm* and *Anxa4* via *Myc* and *Fos* (Fig. 4a). Trp53 is predicted to be downregulated in diminuendo homozygotes, either by a reduction in mRNA or protein levels, or by decreased activity, and either Sdc2 or Mitf might mediate that downregulation. If Trp53 activity can be somehow increased or Sdc2 activity knocked down in diminuendo homozygotes, it might rescue the misregulation of *Ppp1r15a*, *Bhlhe40*, *Gfi1, Slc26a5*, *Ocm* and *Anxa4*.

However, before any conclusions can be drawn, the involvement of these genes with multiple connections (hub genes) has to be checked, for example by examining expression in the hair cells and testing for misregulation of mRNA or protein in diminuendo homozygotes.

From the entire network, we chose interesting nodes (genes) based on the number of their downstream connections, their position within the network, and their potential influence on downstream nodes, particularly *Ocm* and *Slc26a5*. The genes those nodes represented were tested by qRTPCR, and *Fos* was found to be significantly downregulated in diminuendo homozygotes (adjusted P < 0.05), fitting the prediction. None of the other seven tested were significantly affected (Fig. 3b; *Ets1, Tgfb1, Sp1, Myc, Agt, Rhoa, Trp53*), but this doesn’t rule them out as genuine interactors in the miR-96 regulatory network, because they may have altered protein levels or activity, neither of which would be detected by qRTPCR. Myc, Sp1, Ets1 and Fos were also found to be present in wildtype hair cells by immunohistochemistry (Supplementary Fig. S2); no obvious difference in expression was observed in diminuendo homozygote hair cells.

### Protein-protein interactome analysis

The regulatory network uses only regulatory interactions, and does not take advantage of the extensive protein-protein interaction (PPI) data publicly available. A different approach to network construction is to build a PPI network using the publicly available results from many experiments. This has the advantage of being less constrained by bias towards well-studied “favourite genes” such as *Cdc2*^29^ but it does not result in a directional network, or in predictions of up- or downregulation that can be tested. We used InnateDB, a public PPI database combining data from multiple sources, to carry out over-representation and network analyses. In the first analysis we focussed on transcription factor and pathway over-representation, using the misregulated gene list (Supplementary Table S4) plus *Fos*, but no transcription factors or pathways were over-represented after adjustment for multiple testing (adjusted P < 0.05, Supplementary Table S5). We then carried out a network analysis on both the misregulated gene list (Supplementary Table S4 plus *Fos*) and the miR-96 direct target list (Supplementary Table S3) together, to obtain a PPI network which was analysed in Cytoscape (Supplementary Fig. S3), using the Network Analyser tool to calculate node degree, betweenness centrality and closeness centrality (Supplementary Figs S3, S4). The node degree is the number of neighbours a node has; nodes with a high degree are hub nodes. Betweenness centrality is a measure of how important a node is for connecting distant parts of the network, and closeness centrality measures how important a node is within its immediate vicinity.

Eighteen nodes had a high degree (>75, Table 1), but only Ywhae also scored highly on both centrality measures (Table 1, Supplementary Fig. S4). Of the high-degree nodes, five are predicted targets of miR-96 (Foxo3, Foxo1, Rad51, Irs1 and Nr3c1), and five are known to be misregulated in diminuendo homozygotes (Gfi1, Nphp1, Parp1, Fos and Fadd). The others are Ywhae, Ywhah, Ywhaz, Pgls, Hsd3b7, IDB-74, Ubc and Cenpc1. Ywhae, Ywhah and Ywhaz are members of the 14-3-3 protein family, which are ubiquitous adapter proteins, and Ubc is part of the ubiquitination system, so they have many known interaction partners. Pgls is a hydrolase involved in the pentose phosphate pathway^30^, Hsd3b7 is an enzyme required for bile acid synthesis^31^, and Cenpc1 is a component of the kinetochore plate^32^. Their high degree in the PPI network may reflect biological relevance, or it may be, like the 14-3-3 proteins, because they have a lot of interaction partners and among those partners are genes misregulated in the diminuendo homozygote organ of Corti. IDB-74 is an InnateDB identifier representing a large protein complex, which is why it has a high degree.

### Gene Set Enrichment Analysis

Both the regulatory network and the PPI network were constructed knowing the context of the transcriptome data, that it came from a mouse carrying a mutation in miR-96. For both those networks, we chose to add the predicted miR-96 targets with the aim of connecting as many of the misregulated genes to miR-96 as possible. Gene set enrichment analysis, however, takes as input only the misregulated genes, and looks for collections of genes which occur more often than would be expected by chance. These gene sets are predefined by the user, and for this analysis we used the following gene sets available at MsigDB (http://www.broadinstitute.org/gsea/msigdb/index.jsp): the “Hallmark” gene sets which summarise biological states or processes, gene sets from Gene Ontology, KEGG and Reactome, and transcription factor and microRNA target genes (accessed July 30^th^, 2015).

We compared the most upregulated to the most downregulated genes, and also the most affected to the least affected genes, regardless of the direction of regulation. The input data consisted of the genes with a fold change greater than 10% from the P4 and P0 microarray data and the qRTPCR with adjusted P < 0.05. Where multiple probes were present for the same gene, the fold change with the lowest P-value was included, resulting in 2653 genes (Supplementary Table S6). Significantly enriched gene sets (false discovery rate (FDR) < 0.05) are shown in Table 2, as are all the gene sets with nominal P < 0.05.

One of the regulatory factors identified as having targets enriched in the upregulated genes is miR-96, which is a good positive control. Myc is the other factor with a significantly enriched target set among the upregulated genes, and is of particular interest, being downstream of *Gfi1* and upstream of *Fos*, *Slc26a5* and *Ocm* in the regulatory network (Fig. 4a). Among the factors with a nominal P < 0.05 are Egr4, Pou1f1, Tcf3, Cebpb and Vdr, which have been previously linked to inner ear development and/or deafness, as has Myc itself^33^,^34^,^35^,^36^,^37^,^38^. These genes are implicated in mediating the pattern of misregulation detected in diminuendo homozygotes and are, therefore, also candidates for further testing, and potential therapeutic targets. Multiple gene sets representing pathways and processes, which can also be targets for manipulation by drugs or small molecules, were identified as enriched in the input data, including the p53 pathway and synaptic transmission, which fits with our previous reports of synaptic defects in diminuendo homozygotes^6^.

## Discussion

miR-96 is a master regulator of hair cell differentiation, and controls many genes. Here we have used several different tools to identify genes which play important roles in mediating the action of miR-96.

Our manually created regulatory network demonstrates a novel approach to network creation, and the resulting network has several important features. Firstly, some of the genes are independently known to be involved in hearing, such as *Fgf8*, *Slc26a5* (prestin), *Pou4f3* and *Gfi1*^9^,^10^,^39^,^40^, suggesting that other genes in the network may also underlie deafness. Secondly, several of the genes are known to be involved in cellular stress response pathways, such as *Trp53*, *Hif1a* and *Nfe2l2* (reviewed in Simmons *et al.*^41^), suggesting that the network may be perturbed during stress triggered by environmental factors. Thirdly, a number of the network molecules are already known to be targeted by drugs, like Trp53, Nfkb1 and Nr3c1, providing routes to manipulation of the network as a whole.

While most of the misregulated genes detected by microarray and qRTPCR are present in the protein-protein interaction network created using InnateDB, approximately 31% are not, including Prestin, Chrna10 and Pou4f3. This may be because there are important targets of miR-96 that we have not discovered, and so are missing from the initial input, or it may be that the targets are correct but there is insufficient data from previous publications or databases on the interactions between the input molecules. Of the nodes identified as important by network analysis in Cytoscape, Parp1, Gfi1, Nphp1, Fos, Fadd, Nr3c1, Irs1, Rad51, Foxo1 and Foxo3 are present in the regulatory network, and Parp1, Gfi1, Nphp1, Fos and Fadd have been shown by microarray or qRTPCR to be misregulated in diminuendo homozygotes.

The gene set enrichment analysis implicates further regulators and regulatory pathways in the diminuendo phenotype, some of which are present in the regulatory network but most of which are not, suggesting that there are more pathways to be discovered. miR-96 targets (defined by MSigDB as genes sharing the motif GTGCCAA, which matches the wildtype seed region, in their 3′UTRs) are enriched in the upregulated genes, as would be expected. This correlates well with the Sylamer results (Fig. 1). Of the transcription factors whose targets are enriched in the misregulated genes, Egr4, Pou1f1, Tcf3, Myc, Cebpb and Vdr have been previously implicated in inner ear development and/or deafness^33^,^34^,^35^,^36^,^37^,^38^. None of the 31 direct targets of miR-96 (Supplementary Table S3) were implicated by GSEA, which could be because gene sets controlled by the direct targets were not available at the time of analysis, or it could mean that none of the 31 direct targets control the cascade of misregulated genes seen in diminuendo homozygote inner ears. Of the genes implicated by GSEA, only *Atf2* is predicted by StarBase (http://starbase.sysu.edu.cn/index.php)^26^ to be a target of miR-96.

**Figure 6:**
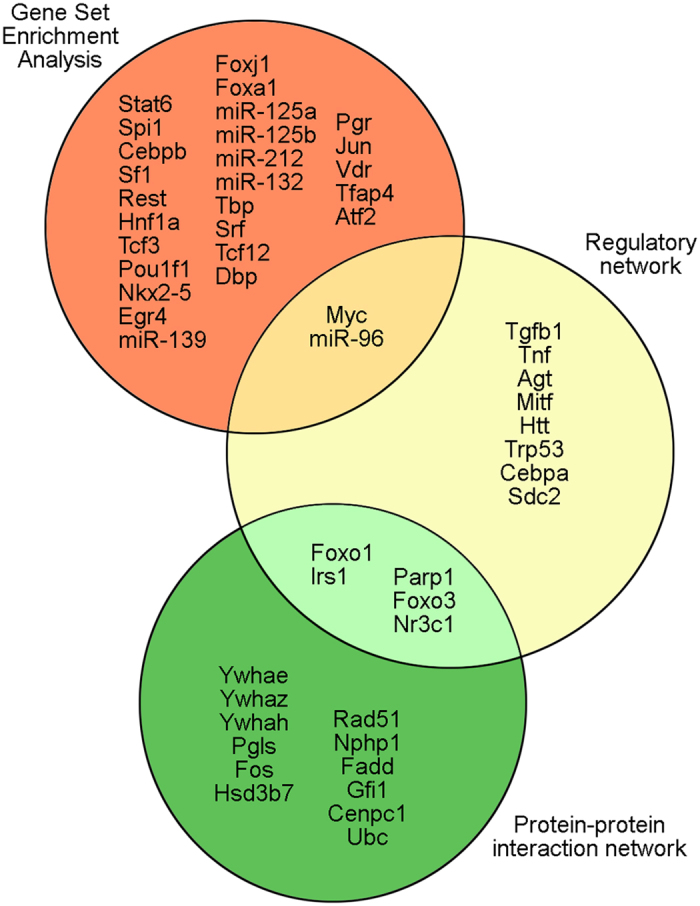
The intersection of genes predicted to be important by the three methods of analysis. 28 genes were detected as having target genes enriched in the microarray data by GSEA, two of which, miR-96 and Myc, are also hub nodes (nodes with more than 7 downstream connections) in the regulatory network. The Cytoscape analysis of the protein-protein interaction network resulted in seventeen genes which were critical to the network, five of which (Parp1, Irs1, Foxo1, Foxo3 and Nr3c1) are hub nodes in the regulatory network.

We have tried three methods of network analysis, all of which identified genes for further testing, but none of which offered a universal solution. Indeed, there is no common gene identified by all three methods as a mediator of the effects of miR-96 (Fig. 6). This reflects the different strengths, biases and weaknesses of the methods used to create the networks. The regulatory network is very dependent on what is reported in the literature, and emphasises connecting miR-96 to the misregulated genes observed in the diminuendo mutant, while the PPI network relies on large databases of protein-protein interactions, which do not offer directional, regulatory links between interacting partners. Gene Set Enrichment Analysis can be useful because it does not rely on correctly identifying the targets of miR-96, many of which remain unknown, but it does need comprehensive gene sets, which may not exist for all the genes mediating the effects of miR-96 in the inner ear. More data may help in the construction of more accurate and comprehensive networks. Transcriptome data from microarrays and RNA-seq, which is available from public databases such as ArrayExpress, may prove useful because they combine directional regulatory interactions with a genome-wide scope, hopefully avoiding at least some of the bias of well-studied genes. The networks are also all subject to a common limitation in that they cannot identify novel interactions, relying as they do on previous studies in other tissues or organisms. The lack of inner ear-specific data is also a potential problem; interactions observed in one type of tissue may not apply to the organ of Corti.

Our analyses implicate *Myc*, *Gfi1* and *Fos* as important candidates; *Fos* regulates both *Slc26a5* and *Ocm*^42^,^43^, two of the most downregulated genes in the P4 microarray^1^, and when we tested it we confirmed its predicted downregulation in diminuendo homozygotes. In the regulatory network *Fos* is controlled by Gfi1 via Myc^44^,^45^, and *Gfi1* is known to be downregulated in diminuendo homozygotes. Myc also regulates *Anxa4*, which is upregulated in diminuendo homozygotes and has recently been found to be specifically expressed in hair cells, along with *Rasd2*, another gene upregulated in diminuendo homozygote organ of Corti^46^. Both *Gfi1* and *Fos* were also identified as important genes in the protein-protein interaction network, and Myc’s targets were detected by GSEA to be significantly enriched in the upregulated genes. All three have been implicated in hearing and deafness, Gfi1 in particular; the hair cells of mice homozygous for a *Gfi1* knockout allele degenerate around P0^10^. Mice homozygous for a null allele of Fos show a reduced startle response at higher frequencies^47^, and Myc has been implicated in protection against noise exposure-induced damage; guinea-pigs inoculated with adenovirus carrying c-myc showed a smaller threshold shift and less cochlear damage after noise exposure than controls inoculated with vector^35^. The other candidate targets are those genes identified by multiple networks (Fig. 6). Further testing *in vitro* would determine whether small molecule agonists or antagonists can modify the expression of these genes, and whether that has an effect further down the line on genes such as *Ocm*, *Ptprq* and *Slc26a5*, whose misregulation in diminuendo homozygotes appears to result in specific aspects of the observed phenotype^6^,^11^.

miR-96 controls many genes, and it is likely that many of the connections between the microRNA and the misregulated genes still remain to be discovered. However, the potential benefits of finding genes and pathways which can be modified by small molecules or drugs are significant. Altering the expression of important genes and pathways may prevent or delay hearing loss caused by mutations in miR-96, or indeed any other trigger which affects the miR-96 network, whether genetic or environmental. Discovering ways to counteract the effect of dysfunctional miR-96 could ultimately lead to therapeutic treatments to delay or prevent progressive hearing loss with a range of aetiologies in the human population.

## Materials and Methods

### Ethics statement

Mouse studies were carried out in accordance with UK Home Office regulations and the UK Animals (Scientific Procedures) Act of 1986 (ASPA) under UK Home Office licences, and the study was approved by both the Wellcome Trust Sanger Institute and the King’s College London Ethical Review Committees. Mice were culled using methods approved under these licences to minimize any possibility of suffering.

### RNA extraction

The organs of Corti of newborn (P0) or four-day-old (P4) mice were dissected during a short time window (6 to 7.5 hours after lights on) and stored at −20 °C in RNAlater stabilisation reagent (QIAgen, cat. no. 76106). RNA was extracted using QIAshredder columns (QIAgen, cat. no. 79654) and the RNeasy mini kit (QIAgen, cat. no. 74104), following the manufacturer’s instructions. RNA concentration was measured using a Nanodrop spectrophotometer (ND-8000).

### Ago2 pulldown

The organs of Corti and olfactory bulbs of mice at P4 were dissected and immediately frozen in liquid nitrogen. Samples were treated as described previously^24^, with the following modifications; samples were homogenized in 300 ul ice-cold buffer, 30 ul of beads were used per sample, prebound to 8.8 ug anti-mouse monoclonal Ago2 antibody (Wako Pure: 018-22021) according to the Dynabead protocol (Invitrogen: 100.07D immunoprecipitation kit). Samples were incubated with antibody for 90 minutes. After immunoprecipitation, 300 ul TRIzol and 60 ul chloroform was added to extract RNA.

### Microarray

For the microarray on P0 mice, organ of Corti samples from six *Dmdo/Dmdo* homozygotes and six sex-matched wildtype littermates were submitted for microarray analysis. For the Ago2 pulldown microarray, samples were pooled in pairs by genotype, resulting in three *Dmdo/Dmdo* homozygote pools and three wildtype pools from each tissue type (organ of Corti and olfactory bulb), and the twelve resulting pools were submitted for microarray analysis. RNA was amplified and purified using the Illumina TotalPrep-96 RNA Amplification kit (Ambion, UK), according to the manufacturer’s instructions. Biotin-labeled cRNA was then normalized to a concentration of 150 ng/ul and 1500 ng was hybridised to Illumina MouseWG-6 v2 beadarrays (Illumina, CA, USA) for 16 hours (overnight) at 58 °C. Following hybridisation, beadarrays were washed and stained with streptavidin-cy3 (GE Healthcare, UK). Beadarrays were then scanned using the Beadarray reader and image data was then processed using Genome Studio software (Illumina, CA, USA). The data were normalised using a quantile normalisation, assuming that the overall intensity distributions of the arrays should be comparable^48^,^49^ and analysed using the LUMI and LIMMA packages from Bioconductor^50^,^51^. P-values were adjusted for multiple tests as described in Benjamini and Hochberg^52^. The microarray data have been submitted to the ArrayExpress database (www.ebi.ac.uk/arrayexpress) and assigned the identifiers E-MTAB-3735 (P0 data) and E-MTAB-3737 (Ago2 pulldown data).

### Immunohistochemistry

P4 and P5 pups were collected, fixed in 10% formalin, embedded in paraffin and cut into 8μm sections. Immunohistochemistry was carried out using a Ventana Discovery machine and reagents according to the manufacturer’s instructions: (DABMap^TM^ Kit (cat.no 760-124), OmniMap DAB anti-Rb Detection Kit (cat.no 760-149), Hematoxylin (cat.no 760-2021), Bluing reagent (cat.no 760-2037), CC1 (cat.no 950-124), EZPrep (cat.no 950-100), LCS (cat.no 650-010), RiboWash (cat.no 760-105), Reaction Buffer (cat.no 95-300), and RiboCC (cat.no 760-107)). For each antibody, at least three wildtype mice were tested, according to the same protocol, and from each animal, at least three sections were used per antibody. Primary antibodies used were: Anxa4 (Abcam, cat.no AB33009, diluted 1:100), Meig1 (Abcam, cat.no AB52012, diluted 1:7.5), Tppp (Abcam, cat.no AB48731, diluted 1:50, Homer1 (Lifespan Biosciences, cat.no LS-C30485, diluted 1:100), Kcna10 (Lifespan Biosciences, cat.no LS-C31214, diluted 1:25), Kalrn (Abcam, cat.no AB2015, diluted 1:5), Gtf2e2 (Abcam, cat.no AB28178, diluted 1:125), Mitf (Abcam, cat.no AB24875, diluted 1:10), Zic2 (Santa Cruz, cat.no sc-28151, diluted 1:10), Htt (Millipore, cat.no MAB2166, diluted 1:500), Fos (Santa Cruz, cat.no sc-52, diluted 1:75), Myc (Santa Cruz, cat.no sc-764, diluted 1:25), Sp1 (Santa Cruz, cat.no sc-59, diluted 1:100), Ets1 (Abcam, cat.no AB59217, diluted 1:100) and Tnc (Santa Cruz, cat.no sc-20932, diluted 1:25). The secondary antibodies were anti-goat (Jackson ImmunoResearch, cat.no 705-065-147, diluted 1:100), anti-rabbit (Jackson ImmunoResearch, cat.no 711-065-152, diluted 1:100), and anti-mouse IgG1 (Epitomics, cat.no 3021-1, diluted 1:500). All antibodies were diluted in staining solution (10% foetal calf serum, 0.1% Triton, 2% BSA and 0.5% sodium azide in PBS). Images were taken using a Zeiss Axioskop 2 microscope with the Plan Neofluar 63× 1.4NA objective, a Zeiss Axiocam camera and the associated Axiocam software. Adobe Photoshop was used to process and prepare images; minimal adjustments were made, including rotation and resizing. Where image settings were altered, the adjustment was applied to the entire image.

### qRTPCR

cDNA was made from normalised organ of Corti RNA using Superscript II Reverse Transcriptase (Invitrogen, cat. no. 11904-018) after treatment with DNAse 1 (Sigma, cat.no: AMP-D1). Quantitative RT-PCR was carried out using probes and reagents from Applied Biosystems (Master Mix: 4364340) and Bio-Rad (SsoFast and SsoAdvanced Master mixes, cat. nos 1725232, 1725281), and the 2^−ΔΔCt^ equation was used to calculate relative expression levels^53^. *Hprt* was used as an internal control, and the quantity of sensory tissue present was checked using *Jag1* or *Ngfr*, which are expressed in supporting cells^1^,^54^,^55^,^56^. Pairs were only used when *Jag1/Ngfr* levels did not differ more than 30% between wildtype and *Dmdo/Dmdo* homozygote littermates. At least three technical replicates of each sample were carried out for each reaction, and each primer/probe set was tested in at least five wildtype-homozygote littermate pairs, with the exception of *Chrna1* and *Clvs1*, which due to problems with the probes had only three biological replicates each. Primer details are in Supplementary table S7; standard primers were designed by primer3^57^ and qRT PCR primers were purchased from Applied Biosystems (Life Technologies). The Wilcoxon rank sum test was chosen to determine significance as a suitable test for small sample sizes and populations of unknown characteristics^58^. P-values were adjusted using the method described in Benjamini and Hochberg^52^ to take multiple testing into account.

### Network construction and analysis software

Networks were generated with the aid of IPA (Ingenuity® Systems, www.ingenuity.com). InnateDB^59^ was used for interactome construction and pathway over-representation analysis with Cytoscape^60^ for visualisation and further analysis, and the Gene Set Enrichment Analysis (GSEA) software^61^ for enrichment analysis. InnateDB and GSEA both provide statistical analyses which are corrected for multiple testing.

## Additional Information

**Accession codes:** ArrayExpress: E-MTAB-3735, E-MTAB-3737.

**How to cite this article**: Lewis, M. A. *et al.* Exploring regulatory networks of miR-96 in the developing inner ear. *Sci. Rep.* **6**, 23363; doi: 10.1038/srep23363 (2016).

## Supplementary Material

Supplementary Information

## Figures and Tables

**Table 1:** Nodes from the protein-protein interaction network with high degree (>75), showing betweenness and closeness scores and their misregulation in diminuendo homozygotes, if known (2 significant figures).

Name	Degree	Betweenness	Closeness	Misregulation (mRNA fold change)	Adjusted p-value
Ywhae	2918	0.81	0.62	n/a	n/a
Pgls	593	0.047	0.45	n/a	n/a
Ywhaz	495	0.047	0.46	n/a	n/a
Hsd3b7	462	0.013	0.35	n/a	n/a
Ywhah	285	0.058	0.44	n/a	n/a
Fos	213	0.046	0.35	−1.5	0.019
Parp1	211	0.050	0.36	1.2	0.094
Nr3c1	207	0.048	0.35	1.3	0.95
IDB-74	146	0.043	0.33	n/a	n/a
Fadd	109	0.032	0.33	1.3	0.013
Cenpc1	99	0.022	0.41	n/a	n/a
Irs1	81	0.020	0.43	n/a	n/a
Rad51	81	0.020	0.33	n/a	n/a
Foxo3	80	0.026	0.43	1.0	0.28
Gfi1	80	0.018	0.33	−1.3	0.0058
Nphp1	80	0.016	0.33	−1.2	0.0726
Ubc	78	0.038	0.37	n/a	n/a
Foxo1	76	0.013	0.35	1.3	1

mRNA fold changes and adjusted p-values are from P0 and P4 microarray analyses, with the exception of *Fos*, for which qRTPCR data is shown (transformed to fold change; see Fig. 3 for data).

**Table 2:** Gene Set Enrichment Analysis results showing gene sets with a nominal P < 0.05 from the microarray and qRTPCR data, divided into genes regulated by regulatory factors, genes associated with cellular locations, genes belonging to defined pathways and genes linked to processes.

Type of gene set	Gene sets which are enriched in:		
	Upregulated genes	Downregulated genes	Most affected genes
Regulatory factor target list	miR-96*, Myc*, Stat6, Spi1, Cebpb	Sf1, Rest, Hnf1a, Tcf3, Pou1f1, Nkx2-5, Atf2, Egr4, Foxj1, Foxa1, miR-125a, miR-125b, miR-212, miR-132	Tbp, Srf, Rest, Tcf12, Hnf1a, Dbp, Pgr, Jun, Vdr, Tfap4, miR-139
Genes associated with cellular location	Ribosome*		Extracellular space
	Extracellular space		
Genes involved in specific pathways	p53 pathway*	Synaptic transmission	Neuroactive ligand receptor transmission
	Peptide chain elongation*		Synaptic transmission
	3′UTR-mediated translational regulation*		Nerve impulse transmission
	Nonsense-mediated decay (enhanced by exon junction complex)*		Peptidase activity
	Srp-dependent cotranslational protein targeting to membrane*		
	Translation*		
	Protein kinase activity		
	Collagen formation		
	RNA binding		
	Extracellular matrix organisation		
	Epithelial-mesenchymal transition*	Spermatogenesis	Spermatogenesis
Genes linked to processes	Influenza viral RNA transcription and replication*	CNS development	Sensory perception
		Neurogenesis	UV response
	Influenza life cycle*	Factors involved in megakaryocyte and platelet production	
	Metabolism of mRNA*		
	Metabolism of proteins		
	Cellular biosynthetic process		
	Skeletal development		
	Positive regulation of cell proliferation		
	Inflammatory response		

*gene sets with FDR < 0.05.

## References

[b1] Lewis M. A. *et al.* An ENU-induced mutation of miR-96 associated with progressive hearing loss in mice. Nat Genet 41, 614–618 (2009). 19363478 10.1038/ng.369 PMC2705913

[b2] Mencia A. *et al.* Mutations in the seed region of human miR-96 are responsible for nonsyndromic progressive hearing loss. Nat Genet 41, 609–613 (2009). 19363479 10.1038/ng.355

[b3] Solda G. *et al.* A novel mutation within the MIR96 gene causes non-syndromic inherited hearing loss in an Italian family by altering pre-miRNA processing. Hum Mol Genet 21, 577–585 (2012). 22038834 10.1093/hmg/ddr493 PMC3259013

[b4] Lumayag S. *et al.* Inactivation of the microRNA-183/96/182 cluster results in syndromic retinal degeneration. Proc Natl Acad Sci USA 110, E507–516 (2013). 23341629 10.1073/pnas.1212655110 PMC3568372

[b5] Weston M. D., Pierce M. L., Rocha-Sanchez S., Beisel K. W. & Soukup G. A. MicroRNA gene expression in the mouse inner ear. Brain Res 1111, 95–104 (2006). 16904081 10.1016/j.brainres.2006.07.006

[b6] Kuhn S. *et al.* miR-96 regulates the progression of differentiation in mammalian cochlear inner and outer hair cells. Proc Natl Acad Sci USA 108, 2355–2360 (2011). 21245307 10.1073/pnas.1016646108 PMC3038748

[b7] Goodyear R. J. *et al.* A receptor-like inositol lipid phosphatase is required for the maturation of developing cochlear hair bundles. J Neurosci 23, 9208–9219 (2003). 14534255 10.1523/JNEUROSCI.23-27-09208.2003 PMC6740823

[b8] Lee S. I. *et al.* A null mutation of mouse Kcna10 causes significant vestibular and mild hearing dysfunction. Hear Res 300, 1–9 (2013). 23528307 10.1016/j.heares.2013.02.009 PMC3684051

[b9] Liberman M. C. *et al.* Prestin is required for electromotility of the outer hair cell and for the cochlear amplifier. Nature 419, 300–304 (2002). 12239568 10.1038/nature01059

[b10] Wallis D. *et al.* The zinc finger transcription factor Gfi1, implicated in lymphomagenesis, is required for inner ear hair cell differentiation and survival. Development 130, 221–232 (2003). 12441305 10.1242/dev.00190

[b11] Chen J. *et al.* A reduction in Ptprq associated with specific features of the deafness phenotype of the miR-96 mutant mouse diminuendo. Eur J Neurosci 39, 744–756 (2014). 24446963 10.1111/ejn.12484 PMC4065360

[b12] van Dongen S., Abreu-Goodger C. & Enright A. J. Detecting microRNA binding and siRNA off-target effects from expression data. Nat Methods 5, 1023–1025 (2008). 18978784 10.1038/nmeth.1267 PMC2635553

[b13] Haflidadottir B. S. *et al.* Upregulation of miR-96 enhances cellular proliferation of prostate cancer cells through FOXO1. Plos One 8, e72400 (2013). 23951320 10.1371/journal.pone.0072400 PMC3741168

[b14] Lin H. *et al.* Unregulated miR-96 induces cell proliferation in human breast cancer by downregulating transcriptional factor FOXO3a. Plos One 5, e15797 (2010). 21203424 10.1371/journal.pone.0015797 PMC3009749

[b15] Riester A. *et al.* ACTH-dependent regulation of microRNA as endogenous modulators of glucocorticoid receptor expression in the adrenal gland. Endocrinology 153, 212–222 (2012). 22128032 10.1210/en.2011-1285

[b16] Xu S., Witmer P. D., Lumayag S., Kovacs B. & Valle D. MicroRNA (miRNA) transcriptome of mouse retina and identification of a sensory organ-specific miRNA cluster. J Biol Chem 282, 25053–25066 (2007). 17597072 10.1074/jbc.M700501200

[b17] Hertzano R. *et al.* Transcription profiling of inner ears from Pou4f3(ddl/ddl) identifies Gfi1 as a target of the Pou4f3 deafness gene. Hum Mol Genet 13, 2143–2153 (2004). 15254021 10.1093/hmg/ddh218

[b18] Winter H. *et al.* Thyroid hormone receptors TRalpha1 and TRbeta differentially regulate gene expression of Kcnq4 and prestin during final differentiation of outer hair cells. J Cell Sci 119, 2975–2984 (2006). 16803873 10.1242/jcs.03013

[b19] Bradley D. J., Towle H. C. & Young W. S. 3rd. Alpha and beta thyroid hormone receptor (TR) gene expression during auditory neurogenesis: evidence for TR isoform-specific transcriptional regulation *in vivo*. Proc Natl Acad Sci USA 91, 439–443 (1994). 8290545 10.1073/pnas.91.2.439 PMC42964

[b20] Gilels F., Paquette S. T., Zhang J., Rahman I. & White P. M. Mutation of Foxo3 causes adult onset auditory neuropathy and alters cochlear synapse architecture in mice. J Neurosci 33, 18409–18424 (2013). 24259566 10.1523/JNEUROSCI.2529-13.2013 PMC6618809

[b21] Xiang M., Gao W. Q., Hasson T. & Shin J. J. Requirement for Brn-3c in maturation and survival, but not in fate determination of inner ear hair cells. Development 125, 3935–3946 (1998). 9735355 10.1242/dev.125.20.3935

[b22] Zuo J. *et al.* Glucocorticoid receptor expression in the postnatal rat cochlea. Hear Res 87, 220–227 (1995). 8567439 10.1016/0378-5955(95)00092-i

[b23] Villarejo-Balcells B., Guichard S., Rigby P. W. & Carvajal J. J. Expression pattern of the FoxO1 gene during mouse embryonic development. Gene Expr Patterns 11, 299–308 (2011). 21397048 10.1016/j.gep.2011.02.002

[b24] Matkovich S. J., Van Booven D. J., Eschenbacher W. H. & Dorn G. W. 2nd. RISC RNA sequencing for context-specific identification of *in vivo* microRNA targets. Circ Res 108, 18–26 (2011). 21030712 10.1161/CIRCRESAHA.110.233528 PMC3017647

[b25] Wang X. miRDB: a microRNA target prediction and functional annotation database with a wiki interface. RNA 14, 1012–1017 (2008). 18426918 10.1261/rna.965408 PMC2390791

[b26] Li J. H., Liu S., Zhou H., Qu L. H. & Yang J. H. starBase v2.0: decoding miRNA-ceRNA, miRNA-ncRNA and protein-RNA interaction networks from large-scale CLIP-Seq data. Nucleic Acids Res 42, D92–97 (2014). 24297251 10.1093/nar/gkt1248 PMC3964941

[b27] Maragkakis M. *et al.* DIANA-microT web server: elucidating microRNA functions through target prediction. Nucleic Acids Res 37, W273–276 (2009). 19406924 10.1093/nar/gkp292 PMC2703977

[b28] Thoenes M. *et al.* OSBPL2 encodes a protein of inner and outer hair cell stereocilia and is mutated in autosomal dominant hearing loss (DFNA67). Orphanet J Rare Dis 10, 15 (2015). 25759012 10.1186/s13023-015-0238-5 PMC4334766

[b29] Edwards A. M. *et al.* Too many roads not taken. Nature 470, 163–165 (2011). 21307913 10.1038/470163a

[b30] Miclet E. *et al.* NMR spectroscopic analysis of the first two steps of the pentose-phosphate pathway elucidates the role of 6-phosphogluconolactonase. J Biol Chem 276, 34840–34846 (2001). 11457850 10.1074/jbc.M105174200

[b31] Shea H. C., Head D. D., Setchell K. D. & Russell D. W. Analysis of HSD3B7 knockout mice reveals that a 3alpha-hydroxyl stereochemistry is required for bile acid function. Proc Natl Acad Sci USA 104, 11526–11533 (2007). 17601774 10.1073/pnas.0705089104 PMC1913850

[b32] Saitoh H. *et al.* CENP-C, an autoantigen in scleroderma, is a component of the human inner kinetochore plate. Cell 70, 115–125 (1992). 1339310 10.1016/0092-8674(92)90538-n

[b33] Bae C. J., Jeong J. & Saint-Jeannet J. P. A novel function for Egr4 in posterior hindbrain development. Sci Rep 5, 7750 (2015). 25583070 10.1038/srep07750 PMC4291570

[b34] Gross J., Angerstein M., Fuchs J., Stute K. & Mazurek B. Expression analysis of prestin and selected transcription factors in newborn rats. Cell Mol Neurobiol 31, 1089–1101 (2011). 21614551 10.1007/s10571-011-9708-z PMC11498639

[b35] Han Y. *et al.* Effect of c-myc on the ultrastructural structure of cochleae in guinea pigs with noise induced hearing loss. Biochem Biophys Res Commun 390, 458–462 (2009). 19785991 10.1016/j.bbrc.2009.09.091

[b36] Karolyi I. J. *et al.* Dietary thyroid hormone replacement ameliorates hearing deficits in hypothyroid mice. Mamm Genome 18, 596–608 (2007). 17899304 10.1007/s00335-007-9038-0

[b37] Masuda M., Pak K., Chavez E. & Ryan A. F. TFE2 and GATA3 enhance induction of POU4F3 and myosin VIIa positive cells in nonsensory cochlear epithelium by ATOH1. Dev Biol 372, 68–80 (2012). 22985730 10.1016/j.ydbio.2012.09.002 PMC3483650

[b38] Zou J. *et al.* Progressive hearing loss in mice with a mutated vitamin D receptor gene. Audiol Neurootol 13, 219–230 (2008). 18259074 10.1159/000115431

[b39] Erkman L. *et al.* Role of transcription factors Brn-3.1 and Brn-3.2 in auditory and visual system development. Nature 381, 603–606 (1996). 8637595 10.1038/381603a0

[b40] Jacques B. E., Montcouquiol M. E., Layman E. M., Lewandoski M. & Kelley M. W. Fgf8 induces pillar cell fate and regulates cellular patterning in the mammalian cochlea. Development 134, 3021–3029 (2007). 17634195 10.1242/dev.02874

[b41] Simmons S. O., Fan C. Y. & Ramabhadran R. Cellular stress response pathway system as a sentinel ensemble in toxicological screening. Toxicol Sci 111, 202–225 (2009). 19567883 10.1093/toxsci/kfp140

[b42] Johnston I. M. *et al.* Regulation of a multigenic invasion programme by the transcription factor, AP-1: re-expression of a down-regulated gene, TSC-36, inhibits invasion. Oncogene 19, 5348–5358 (2000). 11103936 10.1038/sj.onc.1203927

[b43] Yu N. *et al.* Prestin up-regulation in chronic salicylate (aspirin) administration: an implication of functional dependence of prestin expression. Cell Mol Life Sci 65, 2407–2418 (2008). 18560754 10.1007/s00018-008-8195-y PMC2548279

[b44] Duan Z. & Horwitz M. Targets of the transcriptional repressor oncoprotein Gfi-1. Proc Natl Acad Sci USA 100, 5932–5937 (2003). 12721361 10.1073/pnas.1031694100 PMC156304

[b45] Kalra N. & Kumar V. c-Fos is a mediator of the c-myc-induced apoptotic signaling in serum-deprived hepatoma cells via the p38 mitogen-activated protein kinase pathway. J Biol Chem 279, 25313–25319 (2004). 15078869 10.1074/jbc.M400932200

[b46] Burns J. C., Kelly M. C., Hoa M., Morell R. J. & Kelley M. W. Single-cell RNA-Seq resolves cellular complexity in sensory organs from the neonatal inner ear. Nat Commun 6, 8557 (2015). 26469390 10.1038/ncomms9557 PMC4634134

[b47] Paylor R., Johnson R. S., Papaioannou V., Spiegelman B. M. & Wehner J. M. Behavioral assessment of c-fos mutant mice. Brain Res 651, 275–282 (1994). 7922576 10.1016/0006-8993(94)90707-2

[b48] Bolstad B. *Probe Level Quantile Normalization of High Density Oligonucleotide Array Data.* (2001). Available at: http://bmbolstad.com/stuff/qnorm.pdf. (Accessed: 17th February 2016)

[b49] Bolstad B. M., Irizarry R. A., Astrand M. & Speed T. P. A comparison ofnormalization methods for high density oligonucleotide array data based on variance and bias. Bioinformatics 19, 185–193 (2003). 12538238 10.1093/bioinformatics/19.2.185

[b50] Smyth G. K. Linear models and empirical Bayes methods for assessing differential expression in microarray experiments. Stat. Appl. Gen. Mol. Biol. 3 (2004). 10.2202/1544-6115.1027 16646809

[b51] Smyth G. K. In Bioinformatics and Computational Biology Solutions using R and Bioconductor (eds Gentleman R. *et al.*) 397–420 (Springer, 2005).

[b52] Benjamini Y. & Hochberg Y. Controlling the false discovery rate: a practical and powerful approach to multiple testing. J. R. Stat. Soc. Ser. B 57, 289–300 (1995).

[b53] Livak K. J. & Schmittgen T. D. Analysis of relative gene expression data using real-time quantitative PCR and the 2(-Delta Delta C(T)) Method. Methods 25, 402–408 (2001). 11846609 10.1006/meth.2001.1262

[b54] Morrison A., Hodgetts C., Gossler A., Hrabe de Angelis M. & Lewis J. Expression of Delta1 and Serrate1 (Jagged1) in the mouse inner ear. Mech Dev 84, 169–172 (1999). 10473135 10.1016/s0925-4773(99)00066-0

[b55] Mueller K. L., Jacques B. E. & Kelley M. W. Fibroblast growth factor signaling regulates pillar cell development in the organ of corti. J Neurosci 22, 9368–9377 (2002). 12417662 10.1523/JNEUROSCI.22-21-09368.2002 PMC6758064

[b56] Zine A., Van De Water T. R. & de Ribaupierre F. Notch signaling regulates the pattern of auditory hair cell differentiation in mammals. Development 127, 3373–3383 (2000). 10887092 10.1242/dev.127.15.3373

[b57] Untergasser A. *et al.* Primer3–new capabilities and interfaces. Nucleic Acids Res 40, e115 (2012). 22730293 10.1093/nar/gks596 PMC3424584

[b58] Bridge P. D. & Sawilowsky S. S. Increasing physicians’ awareness of the impact of statistics on research outcomes: comparative power of the t-test and and Wilcoxon Rank-Sum test in small samples applied research. J Clin Epidemiol 52, 229–235 (1999). 10210240 10.1016/s0895-4356(98)00168-1

[b59] Breuer K. *et al.* InnateDB: systems biology of innate immunity and beyond--recent updates and continuing curation. Nucleic Acids Res 41, D1228–1233 (2013). 23180781 10.1093/nar/gks1147 PMC3531080

[b60] Cline M. S. *et al.* Integration of biological networks and gene expression data using Cytoscape. Nat Protoc 2, 2366–2382 (2007). 17947979 10.1038/nprot.2007.324 PMC3685583

[b61] Subramanian A. *et al.* Gene set enrichment analysis: a knowledge-based approach for interpreting genome-wide expression profiles. Proc Natl Acad Sci USA 102, 15545–15550 (2005). 16199517 10.1073/pnas.0506580102 PMC1239896

