# Supplementary material for: Exploring regulatory networks of miR-96 in the developing inner ear
Source: Sci Rep. 2016 Mar 18;6:23363. doi: 10.1038/srep23363 (PMC4796898; doi:10.1038/srep23363)
Supplement: Supplementary Information [file srep23363-s1.pdf]

Exploring regulatory networks of miR-96 in the developing inner ear

Morag A. Lewis, Annalisa Buniello, Jennifer M. Hilton, Fei Zhu, William I. Zhang, Stephanie Evans, Stijn van Dongen, Anton J. Enright, Karen P. Steel

Supplementary Figures

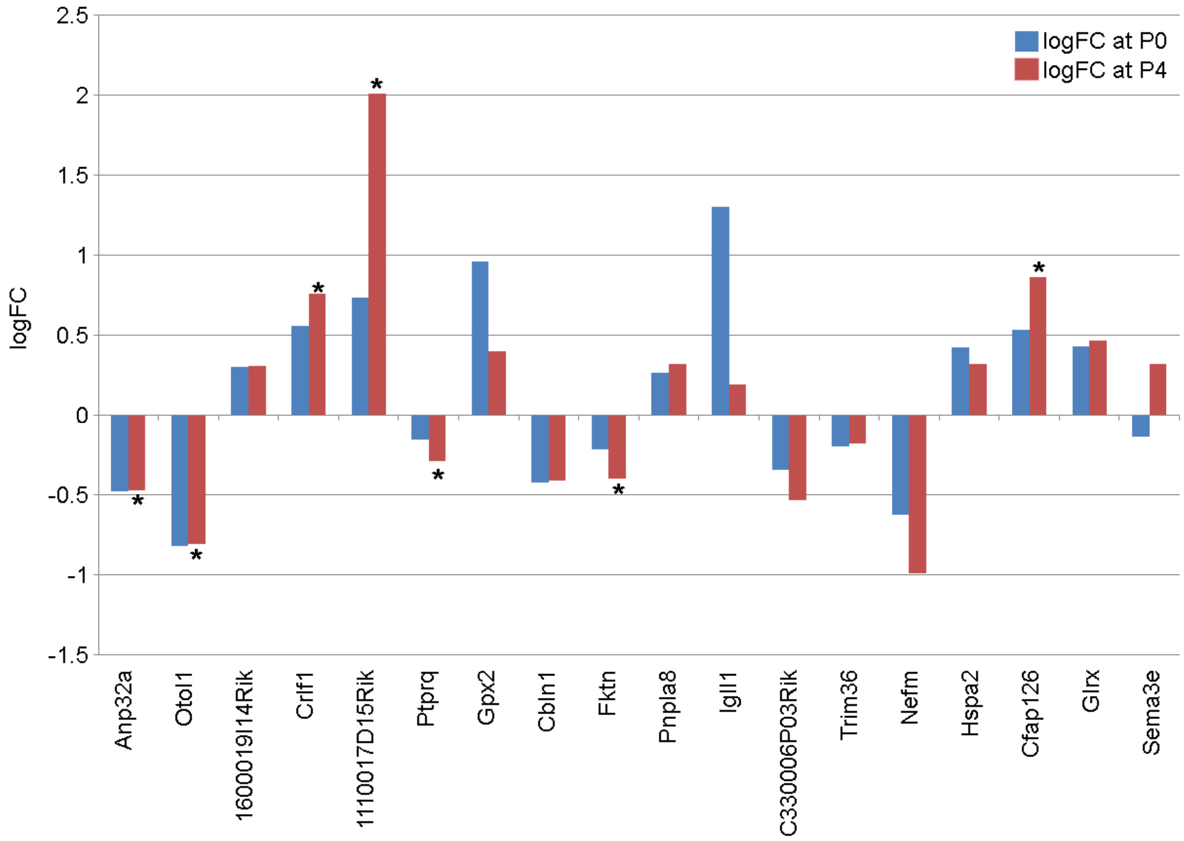

Figure S1. Comparison of the P0 and P4 microarrays. The bar chart shows up- and downregulation of the 18 genes significant in the P0 microarray (blue), compared to their reported regulation in the P4 microarray (red). All genes except Sema3e showed the same direction of regulation at P0 as at P4. Not all the genes which were significantly misregulated in the P0 microarray were also significantly misregulated in the P4 microarray; those which were are indicated by * (adjusted $P < 0.05$). FC = Fold Change in expression level.

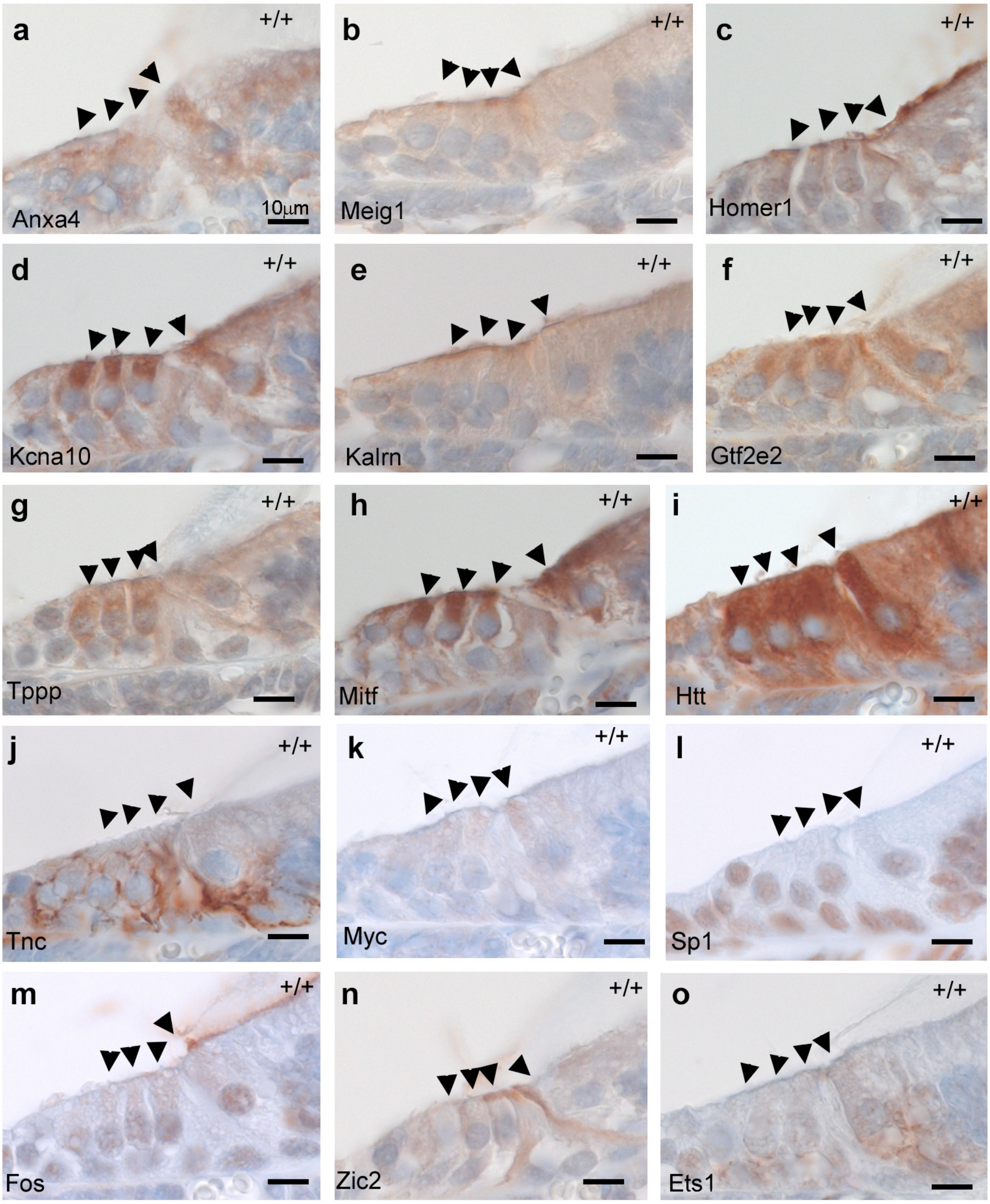

Figure S2. Protein location in the hair cells. Antibody stains on wildtype mice at 5 days old (a-h) and 4 days old (i-o). Tissue is counter-stained in blue to show the structure, and brown stain indicates where the protein is located. Hair cells are indicated by arrowheads. Scale bar = 10μm.

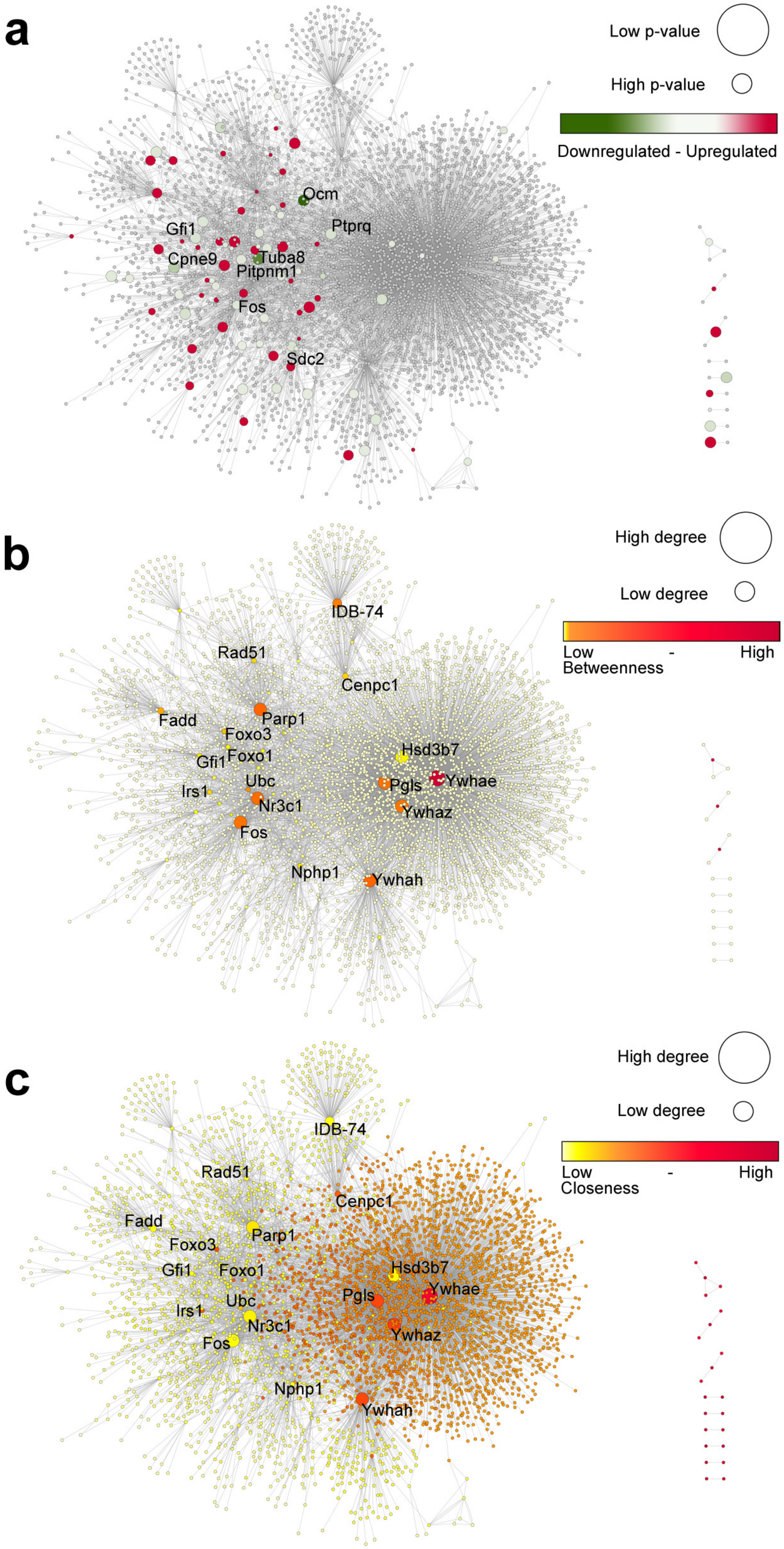

a
Low p-value
High p-value
Downregulated - Upregulated
Ocm
Ptprq
Gfi1
Cpne9
Tuba8
Pitpnm1
Fos
Sdc2
b
High degree
Low degree
Low - High
Betweenness
IDB-74
Rad51
Cenpc1
Fadd
Parp1
Foxo3
Gfi1
Foxo1
Hsd3b7
Ywhae
Ubc
Pgls
Irs1
Nr3c1
Ywhaz
Fos
Nphp1
Ywhah
c
High degree
Low degree
Low - High
Closeness
IDB-74
Rad51
Cenpc1
Fadd
Parp1
Foxo3
Gfi1
Foxo1
Hsd3b7
Ywhae
Pgls
Ubc
Irs1
Nr3c1
Ywhaz
Fos
Nphp1
Ywhah

Figure S3. The protein-protein interaction network generated by InnateDB and laid out in Cytoscape. The small networks to the right represent input genes with one or two known interactors, but which could not be connected to the main network. (a) The network with nodes marked by up- and downregulation (red to green colour) and by significance (the largest nodes being the most significant). (b) The network with nodes coloured by betweenness score, from low (yellow) to high (red). The size of node represents the degree, with the nodes with the highest degree being largest. (c) The network with nodes coloured by closeness score, from low (yellow) to high (dark red). The size of node again represents degree, with the high degree nodes being the largest.

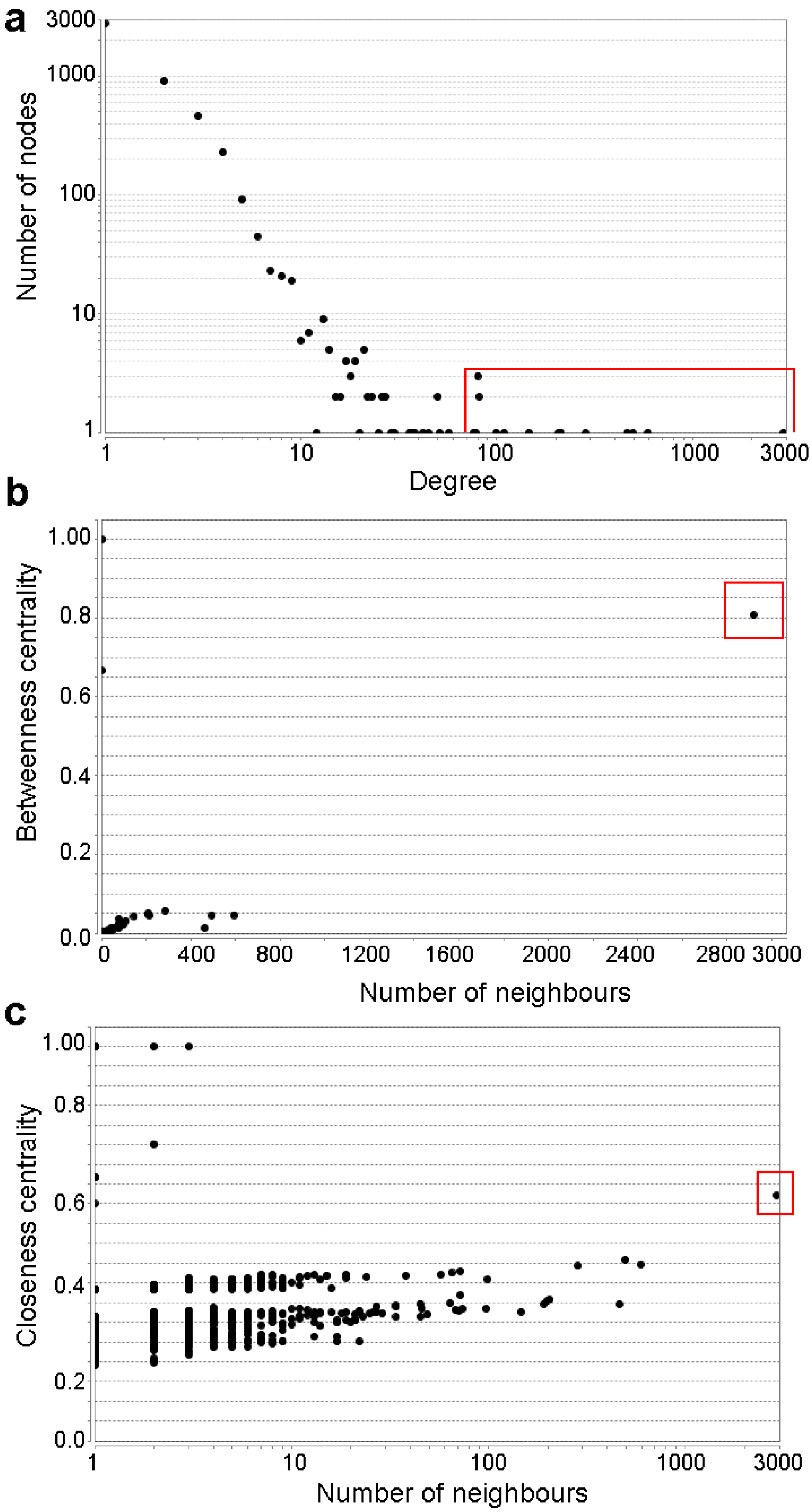
a
Number of nodes
3000
1000
100
10
1
Degree
1
10
100
1000
3000
b
Betweenness centrality
1.00
0.8
0.6
0.4
0.2
0.0
0
400
800
1200
1600
2000
2400
2800
3000
Number of neighbours
c
Closeness centrality
1.00
0.8
0.6
0.4
0.2
0.0
1
10
100
1000
3000
Number of neighbours

Figure S4. Distribution of nodes in the protein-protein interaction networks.(a) The distribution of degree in the protein-protein interaction network. The red box indicates the genes with high degree (>75). (b) The distribution of betweenness centrality in the protein-protein interaction network. Most nodes have few neighbours and a low betweenness centrality (<0.1). (c) The distribution of closeness centrality in the protein-protein interaction network. Most nodes have a similar closeness score (0.2-0.4) regardless of how many neighbours they have. Only Ywhae had a notably high betweenness and centrality score (red box in b,c). Nodes with high betweenness or closeness centrality scores but low degree are those central to the small networks (visible at the right in Fig S3a-c).

Supplementary Tables

Table S1. Table showing the 18 significantly misregulated genes in the P0 microarray, their fold change and adjusted P-value and a comparison to the P4 microarray.

ProbeID	Gene	Chr	logFC	P.Value	adj.P.Val	Misregulation similar at P4?
1090142	Anp32a	9	-0.476155	3.63E-08	0.000753636	Yes
6860161	Otol1	3	-0.820350637	1.16E-06	0.00832001	Yes
4150301	1600029I14Rik	9	0.303498647	1.20E-06	0.00832001	Yes (although not significant)
7380619	Igll1	16	1.306573165	2.52E-06	0.013044536	No
610170	Otol1	3	-0.790878657	4.27E-06	0.016891252	Yes
3780450	Crlf1	8	0.558727254	5.16E-06	0.016891252	Yes
540274	1110017D15Rik	4	0.735348562	5.71E-06	0.016891252	Yes
2360343	C330006P03Rik	13	-0.341791138	6.51E-06	0.016891252	No
6270609	Ptprq	10	-0.15284922	7.34E-06	0.016922029	Yes
3130241	Trim36	18	-0.19890729	1.00E-05	0.020716954	No
4920121	1110017D15Rik	4	0.237243889	1.10E-05	0.020716954	Yes
1820196	Nefm	14	-0.620242384	1.51E-05	0.026135049	No
5260095	Hspa2	12	0.427327576	1.97E-05	0.029519152	No
2680291	Gpx2	12	0.964959665	1.99E-05	0.029519152	Yes (although not significant)
4060072	Cbln1	8	-0.421361398	2.67E-05	0.035634536	Yes (although not significant)
3710328	Fktn	4	-0.211544084	2.77E-05	0.035634536	Yes
1660156	Pnpla8	12	0.264339759	2.92E-05	0.035634536	Yes (although not significant)
4670553	1110017D15Rik	4	0.667308807	3.45E-05	0.03819887	Yes
610709	Cfap126	1	0.534690143	3.73E-05	0.03819887	Yes
5670482	Sema3e	5	-0.137684512	3.83E-05	0.03819887	No
4050112	Glrx	13	0.432214688	3.87E-05	0.03819887	Yes (although not significant)

Table S2. Targets identified as upregulated in wildtype mRNA in the Ago2 pulldown microarray, with their log fold change and unadjusted p values. miR-96 targetting information is included, using the presence of the miR-96 seed region, miRDB, starBase, Diana-microT and miRanda.

					Target in...				
	**Gene name**	**logFC**	**p-value**	**Seeds present**	**miR DB**	**starBase (miRanda, PITA, RNA22, picTar, targetScan)**	**Diana-microT**	**miRanda (annotated)**	**Chosen for testing**
**Organ of Corti**	1520401A03RIK	0.60083178	1.82564E-05						
	MRPS31	0.51282606	0.000864895						
	PTPN22	0.4932611	0.007403266						
	COPZ2	0.45111143	0.000334067						
	ZFP251	0.43743729	0.000494482						Yes
	OLFR1384	0.43559966	0.000350434						
	CCDC39	0.40295931	0.001913311						
	ABLIM1	0.40273578	0.002543472	Yes					Yes
	HACE1	0.39509619	0.00032329						
	A430060F13RIK	0.37820808	0.003554988						
	Serpine3	0.37548445	0.003524418						
	CACNA1C	0.369199	0.008528066	Yes	Yes		Yes		Yes
	XPO4	0.35444894	0.000483995						
	PSMA7	0.35023984	0.002691563						
	TMEM100	0.34498267	0.000696192						
	OLFR530	0.34415201	0.002803871						
	0610007C21RIK	0.34126892	0.007292558						
	PPAP2C	0.33016458	0.000313635						
	D6WSU163E	0.32851156	0.006270356						
	6820408C15RIK	0.32831234	0.001841394	Yes				Yes	Yes
	STARD8	0.32056929	0.000901109						
	OLFR874	0.31640336	0.009489432						
	OLFR66	0.31542495	0.004673802						
	B230380D07RIK	0.31115577	0.006073222						
	GNB4	0.30786546	0.003251329	Yes					Yes
	TMEM97	0.30725867	0.002054388	Yes					Yes
	PLXNA3	0.30636278	0.008855254	Yes					
	ASCC2	0.29952146	0.009544362						
	HGF	0.29763601	0.000238129						
	CTDSPL2	0.29609484	0.008212709						
	SPAG4L	0.29084975	0.006993939	Yes					
	MIA3	0.28972908	0.000330305						
	GTF2H2	0.28927714	0.007778276	Yes					
	D130052B06RIK	0.28552381	0.001901356						

	2310016C08RIK	0.28237428	0.005131174						
	KRT36	0.28113388	0.004173388						
	CNOT2	0.27977169	0.000717741	Yes					
	WDR41	0.27312128	0.009300686						
	ANKRD55	0.27110317	0.007777419	Yes					
	WNT5A	0.26977899	0.003106724	Yes					
	AGA	0.26968742	0.002034159						
	CCDC85B	0.26616714	0.003254573						
	CRNKL1	0.26231892	0.00780573						
	MAGEB18	0.26094404	0.009927157						
	LY6G6E	0.25843237	0.003783373						
	TRUB2	0.25604381	0.005731673	Yes					
	PIGYL	0.25548586	0.000562583						
	IGFL3	0.25521451	0.001706318						
	TIMP1	0.25430756	0.009369112						
	V2R9	0.25334252	0.000792192						
	PRKCH	0.2519283	0.004767421						
	DNAIC1	0.24957044	0.001472168						
	CCT6B	0.24597319	0.007900426						
	WFDC15B	0.24571489	0.004617324						
	9830165L15RIK	0.24038042	0.001211562						
	CSPRS	0.23665974	0.0017935						
	IFT81	0.23651482	0.004595171	Yes					
	AKAP7	0.23556343	0.00106868	Yes					Yes
	UNKL	0.23550101	0.003132373	Yes			Yes		Yes
	2700050L05RIK	0.23409252	0.002500826						
	PSMB7	0.23408854	0.008300336						
	METTL1	0.23254468	0.003721714	Yes					
	Prmt3	0.23101366	0.005207556	Yes					
	ITSN1	0.23022931	0.004664726	Yes					
	NAV3	0.22845984	0.004622507	Yes					
	ENDOV	0.22814058	0.001444749	Yes					
	Sptan1	0.22486157	0.005104234						
	1700049G17RIK	0.22372711	0.000569901						
	OLFR1491	0.2231311	0.00920504						
	SMG6	0.21950005	0.003623197						
	Piezo2	0.21841392	0.000763755						
	ZFP654	0.2174404	0.007459867						
	DOCK6	0.2164	0.001084941						
	ITGA5	0.21495137	0.004685319						
	A130004B21RIK	0.21367746	0.002038166						
	AU018778	0.21326333	0.002866974						
	Nckap5l	0.21318905	0.00904463	Yes					
	Cd300lh	0.21314089	0.003265468						
	CABYR	0.21036108	0.003196232						

	ZFP238	0.20715918	0.009457293							
	UBE2D3	0.20652752	0.007081937	Yes						
	DIAP1	0.20613133	0.004417074							
	E2F1	0.20466072	0.001349189							
	OLFR186	0.20237665	0.002731336							
	EID1	0.20117414	0.007200698							
	RNF185	0.19998114	0.008533872							
	OLFR170	0.19972042	0.008689134							
	NPCD	0.19970292	0.009130174							
	OLFR1079	0.19782997	0.008779822							
	WDR31	0.19771733	0.00331896							
	ALDH8A1	0.1972311	0.007694988							
	PRL2B1	0.19608764	0.003932945							
	NARG1L	0.19406159	0.000418858							
	CDC5L	0.19372488	0.006742926							
	GM9994	0.19255503	0.008968345							
	TMED4	0.19132079	0.001133505							
	OLFR1447	0.18996884	0.001663541							
	3110001I22RIK	0.18902046	0.008706598							
	Gm9391	0.189009	0.008234251							
	2010004A03RIK	0.18648609	0.00607803							
	SLC25A35	0.18604606	0.00627635							
	TCEAL1	0.18594917	0.008148827							
	Ipo5	0.18542285	0.004981213							
	4933421E11RIK	0.18479204	0.006826938							
	CYP2A5	0.1845597	0.005663404							
	MED8	0.18295186	0.00217319							
	Kdm3a	0.18287215	0.008390046							
	DHRSX	0.18230518	0.001297159							
	DNAHC8	0.18179485	0.005793666							
	TIMM13	0.18138043	0.001223398							
	IL18BP	0.17888365	0.009767102	Yes						
	LOC100045280	0.17877914	0.003929907							
	GOSR2	0.17699958	0.00195055	Yes						
	FAM161a	0.17686709	0.0030527	Yes						
	WDR77	0.17670775	0.006412897							
	RDM1	0.17387307	0.00624105							
	PCTK3	0.17341952	0.009058767							
	GSS	0.17255125	0.00847699							
	ALS2CR11	0.1720232	0.004189181							
	Gm7134	0.17123538	0.003100374							
	LOC383240	0.17044079	0.002390373							
	CTAGE5	0.17002904	0.003792136	Yes						
	AI429214	0.16951991	0.009635738	Yes						
	IKBKAP	0.16949902	0.006790133							

	BC048609	0.16935065	0.003898913						
	4931440B09RIK	0.16783967	0.001103159						
	COBLL1	0.16774378	0.006199631	Yes					
	Solh	0.16722793	0.007691681						
	SOX5	0.16580493	0.003451908	Yes	Yes		Yes		Yes
	Tmem182	0.16522817	0.007755306						
	LOC383327	0.16441648	0.008569627						
	TSSK5	0.16416491	0.002901484						
	AMHR2	0.16395339	0.007956769						
	AI842396	0.16383812	0.006178186						
	FAT1	0.16351589	0.003584159						
	ZFP503	0.16187583	0.009599417						
	SLC47A1	0.16159859	0.00531692						
	PPP1R7	0.16075791	0.008912648	Yes					
	4930427A07RIK	0.16063397	0.005293888						
	5031411O12RIK	0.16050378	0.001082101						
	Cntn4	0.16024817	0.001384746						
	PHACTR1	0.15826293	0.005765706						
	ARNTL2	0.1571677	0.005850204						
	PRP15	0.156376	0.008035805						
	IL13RA2	0.15599814	0.00913627						
	TCF7	0.15447556	0.005605959						
	OLFR473	0.15438197	0.007047124						
	CIZ1	0.15430978	0.009897508						
	HPS1	0.15389542	0.003104938	Yes					
	PAX9	0.15371635	0.005262234						
	CD274	0.15338436	0.005100984						
	SNUPN	0.15321306	0.007604394						
	4933439C10RIK	0.15311329	0.0096587						
	SRGAP2	0.15216665	0.003620621						
	Ccdc146	0.15106829	0.005094847						
	B130038J23RIK	0.14906053	0.006825614						
	CCRL1	0.14864507	0.005832847	Yes					
	PRC1	0.1483079	0.003723784	Yes					
	D430031C12RIK	0.14776663	0.00778131						
	ATP6V0A1	0.14698774	0.005373087						
	SEC1	0.14689869	0.002815158						
	2700049P18RIK	0.14678253	0.007350129						
	D230026E03RIK	0.1463289	0.008433852						
	9830107H15RIK	0.14602851	0.005031109						
	MINPP1	0.14580625	0.004975312	Yes					
	C130032M10RIK	0.14533067	0.009538825						
	RTBDN	0.14530755	0.006850283						
	TKT	0.14528446	0.008110255						
	TCRG	0.14505236	0.008229437						

	C430017H16RIK	0.144985	0.008100365						
	C130074O09RIK	0.14435693	0.009190178						
	4921523A10RIK	0.14409202	0.007463156						
	LOC386451	0.14316434	0.007541881						
	H2AFV	0.14315718	0.003212387						
	Gm4746	0.14297811	0.003325455						
	FNBP1	0.14248629	0.0086253	Yes					
	IGHV1S119_L33961_IG_HEAVY_VARIABLE_1S119_14	0.14225324	0.008176115						
	AIFM2	0.14201765	0.005859015	Yes					
	ANKRD27	0.14161135	0.003942218	Yes		Yes	Yes		Yes
	VMN2R4	0.1415653	0.006723207						
	Gm5319	0.14051518	0.004554466						
	MRPL54	0.13928683	0.008248353						
	A430096A09RIK	0.13927998	0.004487655						
	MFSD3	0.13906684	0.008240235						
	FMO2	0.13697303	0.009168699						
	2310043I08RIK	0.13675742	0.007931692						
	NFATC1	0.13351232	0.00769707						
	LOC382514	0.1332091	0.009690478						
	LOC218580	0.1332014	0.008691343						
	SCL0001533.1_63	0.13237579	0.009408001						
	D930046M07RIK	0.1322169	0.009799673						
	B130044D17RIK	0.13158186	0.008888599						
	Kdm5d	0.13101743	0.007079115						
	MOBP	0.12980793	0.009360265	Yes					
	OTTMUSG00000001070	0.12861726	0.004094268						
	SCL0001487.1_50	0.12834807	0.009214326						
	OLFR190	0.12620034	0.004520021						
	GSN	0.12607468	0.00815504						
	NEK1	0.12511225	0.006718731						
**Olfactory bulb**	PSME1	0.23628465	0.000330893						
	PYGO1	0.20551507	0.000905419	Yes					
	PRKCSH	0.20909281	0.002594994						
	FBXO10	0.25245696	0.002921351						
	AI317395	0.19069364	0.0034233	Yes					
	KCNA2	0.13378501	0.003747461						
	2210010N04RIK	0.1533259	0.003820157						
	RABGGTA	0.19136186	0.004539543						
	ZFP42	0.18233886	0.004605196						
	CHADL	0.13701321	0.005442086	Yes					
	C130093N16RIK	0.13167462	0.005449955						

	TMEM47	0.15294077	0.005728007						
	PAFAH2	0.1876717	0.005899386	Yes					
	TRIM33	0.31262959	0.005907365						
	GJB3	0.14589304	0.005940781						
	2010001P08RIK	0.13835879	0.005954361						
	A830009P14RIK	0.16214548	0.006306309						
	PHF7	0.14358327	0.007262192	Yes					
	IL18R1	0.14921928	0.007493511	Yes					
	NDUFS6	0.16184393	0.00757149						
	MPV17L	0.13433217	0.008791176	Yes	Yes		Yes		Yes
	DUSP16	0.14003542	0.009105462						
	CLVS1	0.13708884	0.005978792	Yes			Yes		Yes

Table S3. List of targets of miR-96, both known targets from the literature[13-16,62-74] and predicted targets from expression data in this and previous studies[1,28].

Gene name	Ensembl Gene ID	EntrezGene	mRNA misregulation in Dmdo homozygotes
Adcy6[16]	ENSMUSG00000022994	11512	Unknown
Alk[70]	ENSMUSG00000055471	11682	Unknown
Aqp5[1]	ENSMUSG00000044217	11830	Upregulated
Arf2[1]	ENSMUSG00000062421	11841	Upregulated
Casc1[1]	ENSMUSG00000043541	320662	Upregulated
Celsr2[1]	ENSMUSG00000068740	53883	Upregulated
Clic5[63]	ENSMUSG00000023959	224796	Unknown
Foxo1[13]	ENSMUSG00000044167	56458	Not significant
Foxo3[14]	ENSMUSG00000048756	56484	Not significant
Gad2[1]	ENSMUSG00000026787	14417	Upregulated
Gpc1[69]	ENSMUSG00000034220	14733	Unknown
Gpc3[65]	ENSMUSG00000055653	14734	Unknown
Hbp1[73]	ENSMUSG00000002996	73389	Unknown
Hspa2	ENSMUSG00000059970	15512	Upregulated
Insig2[66]	ENSMUSG00000003721	72999	Unknown
Irs1[67]	ENSMUSG00000055980	16367	Unknown
Mitf[16]	ENSMUSG00000035158	17342	Not significant
Nr3c1[15]	ENSMUSG00000024431	14815	Not significant
Odf2[1]	ENSMUSG00000026790	18286	Not significant
Osbpl2[28]	ENSMUSG00000039050	228983	Not significant
Pnpla8	ENSMUSG00000036257	67452	Upregulated
Rad51[72]	ENSMUSG00000027323	19361	Unknown
Reck[74]	ENSMUSG00000028476	53614	Unknown
Rev1[72]	ENSMUSG00000026082	56210	Unknown
Scarb1[71]	ENSMUSG00000037936	20778	Unknown
Scn3a[62]	ENSMUSG00000057182	20269	Unknown
Sdc2[1]	ENSMUSG00000022261	15529	Upregulated
Slc1a1[68]	ENSMUSG00000024935	20510	Unknown
Spast[64]	ENSMUSG00000024068	50850	Unknown
St8sia3[1]	ENSMUSG00000056812	20451	Upregulated
Zic2	ENSMUSG00000061524	22772	Upregulated

Table S4. Misregulated gene list of genes misregulated in either P0 or P4 microarray (adj. P < 0.1) and genes misregulated in qRTPCR tests (adj. P < 0.05) in this and our previous studies [1,6].

Gene name	Ensembl Gene ID	EntrezGene	Fold change	adj.P.Val	Source
1110017D15Rik	ENSMUSG00000028441	73721	1.664799645	0.016891252	P0 microarray
1110017D15Rik	ENSMUSG00000028441	73721	1.178738658	0.020716954	P0 microarray
1110017D15Rik	ENSMUSG00000028441	73721	1.588107758	0.03819887	P0 microarray
1600029I14RIK	ENSMUSG00000046242	69797	1.234133657	0.00832001	P0 microarray
Cfap126	ENSMUSG00000026649	75472	1.448630989	0.03819887	P0 microarray
Anp32a	ENSMUSG00000032249	11737	-1.391031413	0.000753636	P0 microarray
Arf2	ENSMUSG00000062421	11841	1.258276565	0.059526435	P0 microarray
B3gnt8	ENSMUSG00000059479	232984	1.392456204	0.072222183	P0 microarray
Cbln1	ENSMUSG00000031654	12404	-1.339190685	0.035634536	P0 microarray
Crlf1	ENSMUSG00000007888	12931	1.47296919	0.016891252	P0 microarray
Fktn	ENSMUSG00000028414	246179	-1.157926824	0.035634536	P0 microarray
Glrx	ENSMUSG00000021591	93692	1.34930331	0.03819887	P0 microarray
Gpr88	ENSMUSG00000068696	64378	-1.259618047	0.088588259	P0 microarray
Gpx2	ENSMUSG00000042808	14776	1.952008946	0.029519152	P0 microarray
Hspa2	ENSMUSG00000059970	15512	1.344740294	0.029519152	P0 microarray
Igll1	ENSMUSG00000075370	16136	2.47353303	0.013044536	P0 microarray
Nefm	ENSMUSG00000022054	18040	-1.53713341	0.026135049	P0 microarray
Otol1	ENSMUSG00000027788	229389	-1.765835114	0.00832001	P0 microarray
Otol1	ENSMUSG00000027788	229389	-1.730127857	0.016891252	P0 microarray
Pkig	ENSMUSG00000035268	18769	-1.217327097	0.055921542	P0 microarray
Pnpla8	ENSMUSG00000036257	67452	1.201086254	0.035634536	P0 microarray
Ptprq	ENSMUSG00000035916	237523	-1.111762958	0.016922029	P0 microarray
Sdc2	ENSMUSG00000022261	15529	1.324926776	0.088588259	P0 microarray
Sema3e	ENSMUSG00000063531	20349	-1.100138005	0.03819887	P0 microarray
Serpine3	ENSMUSG00000091155	319433	1.116413463	0.095027336	P0 microarray
Trim36	ENSMUSG00000033949	28105	-1.14782865	0.020716954	P0 microarray
	Not annotated on reference assembly	320588	-1.267329035	0.016891252	P0 microarray
Ocm	ENSMUSG00000029618	18261	-15.88947993	0.00000402	P4 microarray
Cpne9	ENSMUSG00000030270	211232	-2.496661098	0.00444	P4 microarray
Myl1	ENSMUSG00000061816	17901	-2.265767771	0.0522	P4 microarray
Hsd17b7	ENSMUSG00000026675	15490	-2.203810232	0.00575	P4 microarray
Tunar	ENSMUSG00000097929	69952	-1.94126894	0.00948	P4 microarray
Chrna9	ENSMUSG00000029205	231252	-1.911890635	0.00444	P4 microarray
1190003J15Rik	ENSMUSG00000025481	76974	-1.898684242	0.00106	P4 microarray
Olfm1	ENSMUSG00000026833	56177	-1.882956929	0.00356	P4 microarray
Endod1	ENSMUSG00000037419	71946	-1.864772973	0.0000737	P4 microarray
Lmod3	ENSMUSG00000044086	320502	-1.792533399	0.00444	P4 microarray
Tuba8	ENSMUSG00000030137	53857	-1.770307529	0.000688	P4 microarray

LOC229389	ENSMUSG00000027788	229389	-1.744725412	0.00589	P4 microarray
Oc90	ENSMUSG00000015001	18256	-1.735077374	0.0936	P4 microarray
Kcna10	ENSMUSG00000042861	242151	-1.729074463	0.000688	P4 microarray
Tmprss7	ENSMUSG00000033177	208171	-1.686462221	0.00155	P4 microarray
Chrna1	ENSMUSG00000027107	11435	-1.658639092	0.059	P4 microarray
Mmp24	ENSMUSG00000027612	17391	-1.631274987	0.017	P4 microarray
Chrna1	ENSMUSG00000027107	11435	-1.626758396	0.0285	P4 microarray
Slc45a3	ENSMUSG00000026435	212980	-1.586667686	0.000413	P4 microarray
Smim18	ENSMUSG00000094500	72632	-1.565908593	0.0146	P4 microarray
Ppp1r3d	ENSMUSG00000049999	228966	-1.53368266	0.0146	P4 microarray
Gabrb3	ENSMUSG00000033676	14402	-1.528376521	0.0845	P4 microarray
D330017J20Rik	ENSMUSG00000039629	320609	-1.52414483	0.0719	P4 microarray
Slc26a5	ENSMUSG00000029015	80979	-1.522033381	0.00076	P4 microarray
Reep1	ENSMUSG00000052852	52250	-1.520978753	0.0387	P4 microarray
Pitpnm1	ENSMUSG00000024851	18739	-1.491744027	0.000254	P4 microarray
Fam217b	ENSMUSG00000070476	71532	-1.480413298	0.00148	P4 microarray
Slc26a5	ENSMUSG00000029015	80979	-1.473247686	0.00076	P4 microarray
Fam155a	ENSMUSG00000079157	270028	-1.456999114	0.0936	P4 microarray
Homer1	ENSMUSG00000007617	26556	-1.447938172	0.000579	P4 microarray
Pkig	ENSMUSG00000035268	18769	-1.446934886	0.0128	P4 microarray
Fcrlb	ENSMUSG00000070524	435653	-1.445932295	0.0625	P4 microarray
Ppm1h	ENSMUSG00000034613	319468	-1.433955248	0.0436	P4 microarray
Pcsk9	ENSMUSG00000044254	100102	-1.403471726	0.000783	P4 microarray
Nup210	ENSMUSG00000030091	54563	-1.393777239	0.048	P4 microarray
Ghsr	ENSMUSG00000051136	208188	-1.391846392	0.00133	P4 microarray
LOC333088	ENSMUSG00000059022	333088	-1.387030969	0.00627	P4 microarray
2900041A09Rik	ENSMUSG00000021573	72948	-1.386069886	0.00366	P4 microarray
Anp32a	ENSMUSG00000032249	11737	-1.385109468	0.0289	P4 microarray
Dusp8	ENSMUSG00000037887	18218	-1.375541818	0.094	P4 microarray
2210407G14Rik	ENSMUSG00000061751	545156	-1.366987452	0.00948	P4 microarray
Smpx	ENSMUSG00000041476	66106	-1.343503426	0.0471	P4 microarray
Olfm1	ENSMUSG00000026833	56177	-1.342572503	0.019	P4 microarray
Fcmd	ENSMUSG00000028414	246179	-1.316766922	0.0153	P4 microarray
Gabrb3	ENSMUSG00000033676	14402	-1.31494276	0.000227	P4 microarray
Bhlhb2	ENSMUSG00000030103	20893	-1.314031627	0.0387	P4 microarray
4930558C23Rik	ENSMUSG00000105734	67654	-1.313121125	0.0443	P4 microarray
Gfi1	ENSMUSG00000029275	14581	-1.310393404	0.00575	P4 microarray
Srd5a1	ENSMUSG00000021594	78925	-1.28877463	0.0963	P4 microarray
Asb1	ENSMUSG00000026311	65247	-1.275444392	0.00135	P4 microarray
4932416K20Rik	ENSMUSG00000069925	442809	-1.274560627	0.0417	P4 microarray
Selt	ENSMUSG00000075700	69227	-1.273677475	0.0107	P4 microarray
Chrna1	ENSMUSG00000027107	11435	-1.258757174	0.0402	P4 microarray
Rtn2	ENSMUSG00000030401	20167	-1.257013375	0.0522	P4 microarray
Selt	ENSMUSG00000075700	69227	-1.247465572	0.0868	P4 microarray

Hspb3	ENSMUSG00000051456	56534	-1.244874235	0.00205	P4 microarray
Lrp11	ENSMUSG00000019796	237253	-1.237990291	0.0564	P4 microarray
Cpne9	ENSMUSG00000030270	211232	-1.236275261	0.00783	P4 microarray
4931406B18Rik	ENSMUSG00000013353	74054	-1.225185332	0.00759	P4 microarray
Slc34a3	ENSMUSG00000006469	142681	-1.224336392	0.0133	P4 microarray
Ptprq	ENSMUSG00000035916	237523	-1.22010051	0.0164	P4 microarray
Rdh12	ENSMUSG00000021123	77974	-1.218410264	0.0256	P4 microarray
4732474A20Rik	ENSMUSG00000061126	320997	-1.217566019	0.028	P4 microarray
Dnajc16	ENSMUSG00000040697	214063	-1.204972315	0.00759	P4 microarray
Myd116	ENSMUSG00000040435	17872	-1.20163605	0.049	P4 microarray
Ccdc74a	ENSMUSG00000041617	72315	-1.20163605	0.0927	P4 microarray
Dffa	ENSMUSG00000028974	13347	-1.194991205	0.0627	P4 microarray
Abhd4	ENSMUSG00000040997	105501	-1.190856849	0.0748	P4 microarray
Sppl3	ENSMUSG00000029550	74585	-1.187559666	0.0162	P4 microarray
Pcmt1	ENSMUSG00000019795	18537	-1.185914499	0.0351	P4 microarray
Actr1b	ENSMUSG00000037351	226977	-1.183451022	0.0936	P4 microarray
Nphp1	ENSMUSG00000027378	53885	-1.182631	0.0726	P4 microarray
Trak1	ENSMUSG00000032536	67095	-1.180174343	0.0831	P4 microarray
Abca3	ENSMUSG00000024130	27410	-1.17609125	0.0868	P4 microarray
Tpgs2	ENSMUSG00000024269	66648	-1.172834949	0.0927	P4 microarray
Dnajc16	ENSMUSG00000040697	214063	-1.167967395	0.0773	P4 microarray
Leprotl1	ENSMUSG00000031513	68192	-1.165541198	0.0443	P4 microarray
Mrps25	ENSMUSG00000014551	64658	-1.160703914	0.0387	P4 microarray
Mtap6	ENSMUSG00000055407	17760	-1.160703914	0.0754	P4 microarray
Defb5	ENSMUSG00000039785	81007	-1.159095952	0.0516	P4 microarray
Pkd1l2	ENSMUSG00000034416	76645	-1.158292806	0.0936	P4 microarray
Abcg1	ENSMUSG00000024030	11307	-1.152686347	0.084	P4 microarray
A630028F16	ENSMUSG00000035916	237523	-1.152686347	0.0936	P4 microarray
C030025P15Rik	ENSMUSG00000029516	12704	-1.151887642	0.0797	P4 microarray
Galnt13	ENSMUSG00000060988	271786	-1.151089491	0.0307	P4 microarray
Lce6a	ENSMUSG00000086848	78382	-1.147902414	0.0977	P4 microarray
Nceh1	ENSMUSG00000027698	320024	-1.104964485	0.065	P4 microarray
Myo3a	ENSMUSG00000025716	667663	1.099616149	0.0754	P4 microarray
Kif6	ENSMUSG00000023999	319991	1.107264584	0.0831	P4 microarray
Casc1	ENSMUSG00000043541	320662	1.120389214	0.0636	P4 microarray
Tpd52l1	ENSMUSG00000000296	21987	1.126619228	0.095	P4 microarray
Ubxn11	ENSMUSG00000012126	67586	1.131314463	0.0436	P4 microarray
Chst10	ENSMUSG00000026080	98388	1.143138335	0.0797	P4 microarray
Mrps16	ENSMUSG00000049960	66242	1.163926534	0.092	P4 microarray
Slc16a13	ENSMUSG00000044367	69309	1.170398641	0.0698	P4 microarray
Lrch2	ENSMUSG00000031290	210297	1.180174343	0.0733	P4 microarray
Kif9	ENSMUSG00000032489	16578	1.182631	0.0845	P4 microarray
Myl9	ENSMUSG00000067818	98932	1.185092771	0.0636	P4 microarray
Dpp8	ENSMUSG00000032393	74388	1.190856849	0.0522	P4 microarray

Rshl3	ENSMUSG00000039552	212892	1.20163605	0.0433	P4 microarray
Nuak2	ENSMUSG00000009772	74137	1.204972315	0.00948	P4 microarray
Lhx3	ENSMUSG00000026934	16871	1.204972315	0.0834	P4 microarray
1810041L15Rik	ENSMUSG00000062760	72301	1.205807828	0.03	P4 microarray
Gad2	ENSMUSG00000026787	14417	1.20664392	0.092	P4 microarray
D5Ertd593e	ENSMUSG00000047963	52331	1.209155676	0.0575	P4 microarray
Parp1	ENSMUSG00000026496	11545	1.209994089	0.094	P4 microarray
AI505012	ENSMUSG00000036298	239606	1.215036792	0.0754	P4 microarray
C2cd4a	ENSMUSG00000047990	244911	1.218410264	0.0748	P4 microarray
Farp1	ENSMUSG00000025555	223254	1.2397077	0.0754	P4 microarray
AA175286	ENSMUSG00000047735	209086	1.242288282	0.00874	P4 microarray
Pnpla8	ENSMUSG00000036257	67452	1.25092908	0.0754	P4 microarray
Fadd	ENSMUSG00000031077	14082	1.255271991	0.00211	P4 microarray
Ift172	ENSMUSG00000038564	67661	1.257013375	0.0133	P4 microarray
Cfap43	ENSMUSG00000044948	100048534	1.25962998	0.0289	P4 microarray
Fadd	ENSMUSG00000031077	14082	1.278099363	0.0133	P4 microarray
Htr3a	ENSMUSG00000032269	15561	1.281647924	0.00155	P4 microarray
1700010A17Rik	ENSMUSG00000026894	75495	1.293248932	0.0455	P4 microarray
1700007G11Rik	ENSMUSG00000057816	75784	1.304954948	0.0205	P4 microarray
Arf2	ENSMUSG00000062421	11841	1.315854525	0.00444	P4 microarray
Myo3a	ENSMUSG00000025716	667663	1.321338406	0.0261	P4 microarray
Pkhd1l1	ENSMUSG00000038725	192190	1.329607108	0.0289	P4 microarray
Ky	ENSMUSG00000035606	16716	1.36983298	0.0564	P4 microarray
4932425I24Rik	ENSMUSG00000022805	320214	1.388955136	0.0798	P4 microarray
1700024G13Rik	ENSMUSG00000072473	67085	1.396678532	0.00444	P4 microarray
A330021E22Rik	ENSMUSG00000040473	207686	1.396678532	0.0113	P4 microarray
1700016K19Rik	ENSMUSG00000053783	74230	1.397646972	0.0748	P4 microarray
Anxa4	ENSMUSG00000029994	11746	1.399585866	0.0285	P4 microarray
Sdc2	ENSMUSG00000022261	15529	1.418140036	0.00948	P4 microarray
St8sia3	ENSMUSG00000056812	20451	1.478362431	0.0387	P4 microarray
2010001J22Rik	ENSMUSG00000047394	70113	1.479387509	0.03	P4 microarray
1700007G11Rik	ENSMUSG00000057816	75784	1.531557997	0.0726	P4 microarray
Cfap52	ENSMUSG00000020904	71860	1.556170353	0.00575	P4 microarray
Plod2	ENSMUSG00000032374	26432	1.556170353	0.0754	P4 microarray
Asb16	ENSMUSG00000034768	217217	1.604362333	0.000254	P4 microarray
1700024G13Rik	ENSMUSG00000072473	67085	1.611048582	0.0108	P4 microarray
Mup1	ENSMUSG00000078683	100039150	1.634670657	0.00771	P4 microarray
Serpine3	ENSMUSG00000091155	319433	1.636938363	0.00481	P4 microarray
Rasd2	ENSMUSG00000034472	75141	1.659789171	0.0146	P4 microarray
Psat1	ENSMUSG00000024640	107272	1.662091723	0.00627	P4 microarray
1700027A23Rik	ENSMUSG00000010136	76420	1.663244197	0.0205	P4 microarray
Pacrg	ENSMUSG00000037196	69310	1.665551542	0.0625	P4 microarray
Crlf1	ENSMUSG00000007888	12931	1.692317193	0.00948	P4 microarray
Dynlrb2	ENSMUSG00000034467	75465	1.731473131	0.0522	P4 microarray
Golm1	ENSMUSG00000021556	105348	1.73748437	0.00104	P4 microarray

Meig1	ENSMUSG00000026650	104362	1.787570325	0.0461	P4 microarray
1700009P17Rik	ENSMUSG00000026649	75472	1.818816504	0.00759	P4 microarray
Mns1	ENSMUSG00000032221	17427	1.931872658	0.0486	P4 microarray
3100002J23Rik	ENSMUSG00000049154	75429	2.056227653	0.00135	P4 microarray
Egfl9	ENSMUSG00000047428	106565	2.188587403	0.0173	P4 microarray
2310046K01Rik	ENSMUSG00000027463	69698	2.411615655	0.00575	P4 microarray
1110017D15Rik	ENSMUSG00000028441	73721	4.0278222	0.000227	P4 microarray
Anxa4	ENSMUSG00000029994	11746	1.903977782	3.45083E-07	RTPCR
Aqp5	ENSMUSG00000044217	11830	1.398023872	0.01288906	RTPCR
Cacna1d	ENSMUSG00000015968	12289	-1.238237946	0.002534893	RTPCR
Celsr2	ENSMUSG00000068740	53883	1.224905881	0.002781088	RTPCR
Chrna10	ENSMUSG00000066279	504186	1.379892635	3.63E-05	RTPCR
Chrna9	ENSMUSG00000029205	231252	-1.611733132	0.003198482	RTPCR
Cpne9	ENSMUSG00000030270	211232	-3.408384702	7.77069E-12	RTPCR
Gabrb3	ENSMUSG00000033676	14402	-1.775760622	0.009626648	RTPCR
Gfi1	ENSMUSG00000029275	14581	-1.78289743	7.98021E-06	RTPCR
Kcna10	ENSMUSG00000042861	242151	-2.616580519	6.68794E-07	RTPCR
Meig1	ENSMUSG00000026650	104362	3.408656087	0.0277795	RTPCR
Morn5	ENSMUSG00000026894	75495	2.723685528	0.01894375	RTPCR
Ocm	ENSMUSG00000029618	18261	-93.56038613	1.24286E-09	RTPCR
Odf3b	ENSMUSG00000047394	70113	2.509726185	0.0277795	RTPCR
Otof	ENSMUSG00000062372	83762	1.680114181	0.04097	RTPCR
Otol1	ENSMUSG00000027788	229389	-1.220161793	0.002939052	RTPCR
Pitpnm1	ENSMUSG00000097096	18739	-1.410619719	0.009406203	RTPCR
Pou4f3	ENSMUSG00000024497	18998	1.683127656	2.78E-02	RTPCR
Ptprq	ENSMUSG00000035916	237523	-1.79836624	6.46202E-05	RTPCR
Sdc2	ENSMUSG00000022261	15529	1.367028665	0.025898496	RTPCR
Slc26a5	ENSMUSG00000029015	80979	-84.71715955	6.17879E-11	RTPCR
Tmc1	ENSMUSG00000024749	13409	-1.18771812	0.04097	RTPCR
Tuba8	ENSMUSG00000030137	53857	-7.93832868	7.68619E-13	RTPCR
Bhlhe40	ENSMUSG00000030103	20893	-1.335227092	0.01894375	RTPCR
Rasd2	ENSMUSG00000034472	75141	2.055058077	0.0277795	RTPCR
Dynlrb2	ENSMUSG00000034467	75465	3.12074543	0.0277795	RTPCR
Myo3a	ENSMUSG00000025716	667663	4.3283585	0.01894375	RTPCR
Zic2	ENSMUSG00000061524	22772	1.399641645	0.0277795	RTPCR

Table S5. InnateDB over-representation analysis of transcription factor binding sites (a) and pathways (b) implicated in the genes misregulated in either microarray (adj. P < 0.1) and in qRTPCR tests (adj. P < 0.05).

**a) Transcription Factor Binding Site Over-Representation Analysis**		
**Transcription Factor**	**TFBS ORA P-Value**	**TFBS ORA P-Value (Corrected)**
Esrra	0.008808966	1
AP-4	0.009457055	0.983533737
Myog	0.011567622	0.802021767
AP-2	0.022054202	1
Myb	0.035080538	1
Nkx6-2	0.041848173	1
Cebpb	0.044673676	1
Trp53	0.049053364	1

**b) Pathway Over-Representation Analysis**			
**Pathway Name**	**Source**	**Pathway p-value**	**Pathway p-value (corrected)**
ABCA transporters in lipid homeostasis	REACTOME	1.56E-03	0.101349392
EPH-Ephrin signaling	REACTOME	7.02E-03	0.228109625
ABC-family proteins mediated transport	REACTOME	0.008745114	0.189477461
EPHB-mediated forward signaling	REACTOME	0.013866997	0.225338699
Axon guidance	REACTOME	0.014969441	0.194602738
O-linked glycosylation of mucins	REACTOME	0.018146654	0.196588752
Neuroactive ligand-receptor interaction	KEGG	0.018534086	0.172102228
Transmembrane transport of small molecules	REACTOME	0.021425871	0.174085201
Muscle contraction	REACTOME	0.021920411	0.158314078
ABC transporters	KEGG	0.025989578	0.153574778
O-linked glycosylation	REACTOME	0.025989578	0.153574778
Semaphorin interactions	REACTOME	0.041088017	0.222560093
MAPK signaling pathway	KEGG	0.048704168	0.243520841

Table S6. List of genes used for gene set enrichment analysis, taken from the qRTPCR data and the normalised genes in both microarrays[1,6] with a fold change greater than 10%.

Gene symbol	Fold change
OCM	-93.56038613
SLC26A5	-84.71715955
TUBA8	-7.93832868
CPNE9	-3.408384702
KCNA10	-2.616580519
A730016F12RIK	-2.496661098
MAG	-2.394957409
FA2H	-2.234574276
HSD17B7	-2.203810232
MBP	-2.158456473
SNAP25	-2.158456473
PMP22	-2.084931522
NEFH	-2.056227653
PRX	-1.987562187
2810011L19RIK	-1.94126894
1190003J15RIK	-1.898684242
PDXP	-1.884262548
2310067E08RIK	-1.864772973
CALB2	-1.837825767
PTPRQ	-1.79836624
GLDN	-1.797510253
LMOD3	-1.792533399
GFI1	-1.78289743
LOC229389	-1.744725412
EGR2	-1.730273381
CLDN19	-1.714752073
6330417K15RIK	-1.710004356
TMPRSS7	-1.686462221
MMP24	-1.631274987
CHRNA1	-1.626758396
HSPA12A	-1.622254311
D330005G19RIK	-1.617762697
CHRNA9	-1.611733132
ART3	-1.607701981
DHH	-1.593280193
POU3F1	-1.593280193
2210413P12RIK	-1.586667686
AW046396	-1.581178233
PCDH20	-1.568080908

Gene symbol	Fold change
CCL7	-1.126619228
KIF23	-1.126619228
TMEM41A	-1.126449299
CRYAB	-1.12625087
2310043K02RIK	-1.125838586
EEF1A2	-1.125838586
RYBP	-1.125838586
SHROOM2	-1.125838586
9030205A07RIK	-1.125838586
UBE4A	-1.125838586
MGST1	-1.125838586
HS3ST3B1	-1.125718718
AP3B2	-1.12534999
CHGB	-1.12522104
CPEB3	-1.125058485
PRMT3	-1.125058485
3222402P14RIK	-1.125058485
CAI	-1.125058485
DGAT2	-1.12496814
DYRK1B	-1.124782168
FARP2	-1.124670656
DPM1	-1.124448558
KCNC4	-1.124278924
E430013O13RIK	-1.124278924
7530428D23RIK	-1.124278924
C130099A20RIK	-1.124278924
ACTL6A	-1.124278924
0610010O12RIK	-1.124278924
1190004M23RIK	-1.124278924
9530090G24RIK	-1.124278924
LOC381980	-1.124278924
MYOZ1	-1.124278924
TNFRSF19	-1.123842799
CLK1	-1.123766607
MIA3	-1.123499903
AW112037	-1.123499903
PCDHB14	-1.123499903
NCALD	-1.123499903
FMN2	-1.123499903

Gene symbol	Fold change
RPS3A	1.122721422
MAS1	1.122721422
AI415282	1.122721422
LOC386330	1.122721422
BSCL2	1.122721422
MRPL17	1.122721422
4933413G19RIK	1.122721422
CD93	1.122721422
SCXA	1.122721422
ACOX2	1.122721422
TCF4	1.122925461
A230070D14RIK	1.123058777
WWC1	1.123060546
SUMO1	1.123249177
PSMD14	1.123499903
GSTA1	1.123499903
RPL18A	1.123499903
LOXL1	1.123499903
AP3S1	1.123499903
KRT8	1.124048221
1810027O10RIK	1.124091737
ALOX12	1.124278924
GNB2L1	1.124278924
LOC224813	1.124278924
LOC235979	1.124278924
NTN3	1.124278924
PHF6	1.124278924
PSTPIP1	1.124278924
BIRC5	1.124278924
IMPA2	1.124278924
IRX2	1.124323987
ANXA3	1.124910241
2810423O19RIK	1.125058485
E030030I06RIK	1.125058485
NOS3	1.125058485
1700010L19RIK	1.125058485
GPR125	1.125058485
SNRPB	1.125058485
KAZALD1	1.125058485

AI850995	-1.566994374	DACT2	-1.123499903	SLC39A10	1.125058485
2700090O03RIK	-1.565908593	TMEM24	-1.123499903	SNX5	1.125058485
AI427515	-1.565908593	A930019L04RIK	-1.123499903	CHST11	1.125058485
C20ORF185	-1.561572985	UBE2Q2	-1.123390984	TCEA3	1.125058485
NEFM	-1.560351822	PCSK5	-1.122867602	PIK3R3	1.125058485
GM691	-1.550786413	IGSF9	-1.122851204	HSD3B7	1.125058485
SNCB	-1.548638056	SPOP	-1.122721422	ARHGEF6	1.125058485
A330049M08RIK	-1.546492675	AGRN	-1.122721422	COPZ1	1.125058485
MYL1	-1.541759732	5033421J10RIK	-1.122721422	VAMP8	1.125195343
2610024M03RIK	-1.53368266	TMOD4	-1.122721422	CYB5R1	1.125838586
NDRG1	-1.531557997	RBMS1	-1.122721422	LOC226283	1.125838586
PCYT1B	-1.531557997	SEMA4G	-1.122721422	CASP7	1.125838586
D330017J20RIK	-1.52414483	PPP1R1A	-1.122721422	ARHGEF19	1.125838586
D6ERTD253E	-1.520978753	UBN1	-1.122721422	WDR34	1.125838586
DIRAS1	-1.515716567	2900024O10RIK	-1.122526387	ETL4	1.125925459
A330097E02RIK	-1.510472586	CD24	-1.121943481	FAM107A	1.12605788
EGFL8	-1.506290467	AW049604	-1.121943481	CD200	1.126208826
MYCBPAP	-1.504719851	UBE4B	-1.121943481	MAP3K7IP2	1.126333025
CHRNA4	-1.504203751	EPHB6	-1.121943481	UNC45A	1.126345291
LOC232400	-1.502119927	9130415E20RIK	-1.121943481	1110007C02RIK	1.126619228
FOS	-1.500857759	CIAPIN1	-1.121943481	C330034C07RIK	1.126619228
ALDH1A3	-1.498812776	LOC384146	-1.121943481	CACNA1G	1.126619228
2310046A06RIK	-1.497960934	5430433G21RIK	-1.121943481	TPD52L1	1.126619228
PITPNM1	-1.491744027	DSCR1L1	-1.121943481	INO80B	1.126627489
ELAVL2	-1.488645255	LOC381036	-1.121943481	PLVAP	1.127400412
DYSFIP1	-1.482253283	BC039161	-1.121943481	LOC269251	1.127400412
CRHBP	-1.481439798	GNB5	-1.121943481	ZFP291	1.127400412
ANK1	-1.480413298	PLEKHA4	-1.121895771	PPP2R5A	1.127400412
9030418K01RIK	-1.480413298	4732458O05RIK	-1.121166078	ATP6V1B1	1.127400412
BC046386	-1.479387509	SESTD1	-1.121166078	NDUFB10	1.127400412
PLLP	-1.471206746	9330175B01RIK	-1.121166078	DLX6	1.127400412
LOC327956	-1.468150636	ARHGAP24	-1.121166078	CAP1	1.127400412
4933402K11RIK	-1.468150636	2510008P16RIK	-1.121166078	GM687	1.127518439
INTS6	-1.463071221	2310010I15RIK	-1.121166078	MYRIP	1.128182137
BCAS1	-1.456999114	P2RY2	-1.121166078	IMMP2L	1.128182137
AW121567	-1.456999114	KLF9	-1.121166078	PAPSS2	1.128182137
C330006P03RIK	-1.447938172	A530062K18RIK	-1.121166078	1190002H23RIK	1.128182137
PKIG	-1.446934886	AJ237586	-1.121166078	LEPREL2	1.128182137
GJA9	-1.443929196	LSAMP	-1.121166078	1700029G01RIK	1.128182137
1810028F09RIK	-1.440929749	1110053F04RIK	-1.121166078	0610009J22RIK	1.128182137
E230022H04RIK	-1.440929749	2810423E13RIK	-1.121166078	IFI30	1.128964405
NDRL	-1.434949535	BZW1	-1.121166078	RAB12	1.128964405
C030002B11RIK	-1.433955248	TTLL4	-1.121166078	D15MGI27	1.128964405
B930076A02	-1.43296165	CHST2	-1.120591609	TPX2	1.128964405
NDRG4	-1.43097652	BCL7A	-1.120389214	SOX8	1.128964405

6430704N06	-1.429984986	D11ERTD707E	-1.120389214	USE1	1.128970772
D5BWG0860E	-1.421092043	ARL1	-1.120389214	RPL19	1.129215922
STXBP1	-1.419123356	HSBP1	-1.120389214	LOC333137	1.129747215
FABP3	-1.414213562	WNT9A	-1.120389214	SESN3	1.129747215
NPY	-1.411275843	UGT1A10	-1.120227913	4833411O04RIK	1.129747215
TRIM45	-1.410178634	ZCCHC3	-1.119612889	2900093K20RIK	1.129747215
KCNA1	-1.4063932	SPATA13	-1.119612889	ITIH2	1.129747215
GAS2L3	-1.4063932	EFCBP2	-1.119612889	HIST1H2AI	1.129747215
PCSK9	-1.403471726	E130107N23RIK	-1.119612889	SYPL1	1.129747215
ADAM23	-1.402499251	CXADR	-1.119612889	CENPA	1.129747215
AQP11	-1.400556321	GUCY1B3	-1.119612889	RPL27A	1.129747215
PVALB	-1.398616083	BRUNOL4	-1.119612889	TBX1	1.129769958
NGFR	-1.395710764	GRID2	-1.119612889	NFIC	1.130530567
RBP7	-1.395710764	FBXO21	-1.119612889	ANAPC5	1.130530567
NUP210	-1.393777239	CPZ	-1.119564901	FCHSD2	1.130530567
ANP32A	-1.390654387	RPN2	-1.119396176	2610307O08RIK	1.130530567
SPOCK3	-1.389577877	PRC1	-1.119289823	9130210N20RIK	1.130530567
NAP1L5	-1.388955136	TESC	-1.118949707	D4S234E	1.130530567
SV2A	-1.387992719	CYBRD1	-1.118837101	GM2A	1.130530567
AI839562	-1.387992719	LOC385625	-1.118837101	LOC241621	1.130628715
LOC333088	-1.387030969	LOC382069	-1.118837101	ID1	1.130733187
2900041A09RIK	-1.386069886	6430548M08RIK	-1.118837101	MYO7A	1.131314463
5830411K21RIK	-1.381274448	9430076M13	-1.118837101	MCM5	1.131314463
CALB1	-1.379360922	CDH15	-1.118837101	DUSP19	1.131314463
DUSP8	-1.375541818	GALNT2	-1.118837101	1700024K14RIK	1.131314463
POLE	-1.375541818	SALL1	-1.118837101	CDH1	1.131314463
2900074C18RIK	-1.375541818	LIPH	-1.118485962	TMEM4	1.131314463
DUSP15	-1.374588696	UBE3B	-1.118457738	D4BWG1540E	1.131314463
SNURF	-1.373636233	C920006C10RIK	-1.118430364	LOC380925	1.131314463
GAL3ST1	-1.370782805	LBH	-1.118061851	PRKX	1.132091884
1500001L03RIK	-1.367935304	0610025P10RIK	-1.118061851	E030024M05RIK	1.132098902
RNASE1	-1.366987452	3010027A04RIK	-1.118061851	FUT2	1.132098902
MT1A	-1.366987452	CHCHD10	-1.117945329	C920005C14RIK	1.132098902
2210407G14RIK	-1.366987452	LOC100043555	-1.117877174	NOLA3	1.132098902
CADPS	-1.366040257	ACO2	-1.117468157	2310005N03RIK	1.132098902
CPLX1	-1.366039502	MELA	-1.117328316	SLC41A3	1.132098902
8430415E04RIK	-1.365093718	EIF4G3	-1.117287138	CDK4	1.132098902
1110030H18RIK	-1.365093718	GSN	-1.117287138	9530058B02RIK	1.132098902
PRKCQ	-1.365093718	GNG3	-1.117287138	IBRDC2	1.132098902
MTAP1B	-1.365093718	E230013M07RIK	-1.117287138	SELM	1.132098902
CPT1B	-1.364147835	A730089K16RIK	-1.117287138	CAPNS1	1.132847617
CABP2	-1.363202607	E330009J07RIK	-1.117287138	ESAM1	1.132883885
P4HA2	-1.363173089	SLC2A3	-1.117287138	GSTT2	1.132883885
A330066M24RIK	-1.362258035	PHGDH	-1.117098908	PPM1F	1.132883885
SNCG	-1.358009833	NECAP1	-1.116600573	PPIC	1.132883885

TMEM25	-1.35754498
4933428A15RIK	-1.35754498
ATP6V1G2	-1.35754498
DKK3	-1.350037985
USH1C	-1.349102534
FNDC5	-1.349102534
SMPX	-1.343503426
OLFM1	-1.342572503
DPP7	-1.342572503
HOMER2	-1.339783602
CBLN1	-1.339453059
CYP26B1	-1.337927555
SEMA3B	-1.337927555
CLDN12	-1.336074078
BHLHB2	-1.335227092
WFDC12	-1.335158246
9030624O13RIK	-1.333298677
4930403O06RIK	-1.333298677
TMEM27	-1.332374825
UCHL1	-1.332374825
SLITRK1	-1.328685814
KCNAB2	-1.328685814
SYP	-1.327765158
6030405A18	-1.326845141
LOC385255	-1.32592576
CPNE6	-1.322074237
DNAJC6	-1.318593614
FCMD	-1.316766922
DPP10	-1.315854525
GABRB3	-1.31494276
RESP18	-1.314031627
4930558C23RIK	-1.313121125
CUEDC2	-1.312211255
PAK3	-1.309485423
9630025B04RIK	-1.309485423
SLC17A7	-1.306765254
MEIS1	-1.304954948
ELAVL4	-1.304050735
EMILIN2	-1.304050735
FRMD3	-1.303147149
MPP3	-1.30224419
5530400B01RIK	-1.297738767
ITGB6	-1.297738767
2310031A18RIK	-1.296839555
3110004L20RIK	-1.296839555

C230080E09RIK	-1.116512962
LOC383414	-1.116512962
4833441J24RIK	-1.116512962
CUGBP2	-1.116512962
SNX25	-1.116512962
1700045I19RIK	-1.116512962
TMPRSS6	-1.116512962
2700087H15RIK	-1.116512962
AW547365	-1.116512962
LOC381582	-1.116512962
KIAA1715	-1.116512962
SLC27A1	-1.116512962
NSDHL	-1.116512962
2610020H15RIK	-1.116512962
PIP5K2A	-1.116512962
SVOP	-1.116333385
C730029A08RIK	-1.11622149
1500015O10RIK	-1.115850146
SNW1	-1.115739322
0610008A10RIK	-1.115739322
BTN2	-1.115739322
PRSS12	-1.115739322
EMCN	-1.115739322
B130021B11RIK	-1.115739322
1110012E06RIK	-1.115739322
2310014F06RIK	-1.115739322
D15WSU169E	-1.115739322
MPV17	-1.115739322
SNTB1	-1.115739322
STARD3	-1.115426706
SLC24A2	-1.115371408
6530401P13	-1.114966219
LRRTM1	-1.114966219
SLC6A8	-1.114966219
KCNQ2	-1.114966219
FURIN	-1.114966219
A630053H17RIK	-1.114966219
3010027N08RIK	-1.114966219
2200001I15RIK	-1.114966219
ADAMTS5	-1.114966219
PDE6D	-1.114966219
2510009E07RIK	-1.114966219
GLS	-1.114966219
ATP2A2	-1.114966219
4631403P03RIK	-1.114966219

C76566	1.132883885
RHOC	1.132883885
SMPDL3B	1.132883885
CCDC53	1.133069008
HINT2	1.133493652
NDRG2	1.133669413
RPL13A	1.133669413
DGKK	1.133724419
PLEKHF2	1.134101952
9130430L19RIK	1.134455485
ATAD3A	1.134455485
LOC383826	1.134455485
1700003E16RIK	1.134455485
NBL1	1.134660449
PKIA	1.135106605
SI	1.135242102
GART	1.135242102
DCTD	1.135242102
PDE8A	1.135242102
ATP5E	1.135242102
GNA13	1.135404726
BLVRB	1.135729815
TNFAIP8	1.136029265
SCYE1	1.136029265
CBR1	1.136029265
NPN1	1.136029265
BC010801	1.136029265
1110028E10RIK	1.136029265
LOC270589	1.136029265
LYPLAL1	1.136029265
AVPI1	1.136029265
RPL38	1.136029265
6720469N11RIK	1.136029265
B230396O12RIK	1.136029265
CLSTN3	1.136177429
2310016M24RIK	1.136237157
LOC100046800	1.136419429
RELN	1.136555705
RPS6	1.136816973
RPL10A	1.136816973
EXOSC10	1.136816973
SLC37A1	1.136816973
AOC3	1.136816973
NLGN2	1.136816973
5330430P07RIK	1.136816973

CAR7	-1.295940965
PLTP	-1.294145654
6030405A18RIK	-1.291709363
TRH	-1.291048613
SPNB1	-1.289668251
SCAMP5	-1.289668251
SCNN1G	-1.28877463
2810425F24RIK	-1.28877463
SRD5A1	-1.28877463
AA960436	-1.287079806
SNRPN	-1.286989247
C230009H10RIK	-1.286989247
ALS2CR13	-1.285206337
AATK	-1.282536603
C030027L06RIK	-1.279872414
ST8SIA5	-1.279872414
SOSTDC1	-1.279872414
B130052G07RIK	-1.278985581
2310040C09RIK	-1.278099363
GYPC	-1.278099363
RAB3A	-1.276328769
ASB1	-1.275444392
LOC234668	-1.274560627
B3GALT5	-1.274560627
SNAP91	-1.273677475
C130023K05RIK	-1.273677475
AI464131	-1.273677475
TFRC	-1.272168955
TMPRSS5	-1.271913007
1500001H12RIK	-1.271031689
PPP1R14C	-1.270150983
SV2B	-1.270150983
GPX3	-1.270150983
4833424K13RIK	-1.269270886
AI316787	-1.269270886
GPR88	-1.268206443
SLC24A3	-1.267512522
5930403L14RIK	-1.265756594
SYT11	-1.265756594
F830020C16RIK	-1.264879542
9330186A19RIK	-1.264003098
STAC2	-1.263127262
EXPI	-1.263127262
LY6H	-1.263127262
6330439K17RIK	-1.263127262

BAG2	-1.114966219
EXOC6	-1.114966219
CRSP2	-1.114966219
LYPD1	-1.114741827
GLB1	-1.114573881
FBXO2	-1.114323205
A830073O21RIK	-1.114193651
MBOAT1	-1.114193651
LPHN1	-1.114193651
A630066F11RIK	-1.114193651
DDX25	-1.114193651
A930031L14RIK	-1.114193651
ZFYVE20	-1.114193651
PSMD1	-1.113840864
2610029G23RIK	-1.113629149
FEZ1	-1.113421618
CHN2	-1.113421618
EIF5B	-1.113421618
AFG3L1	-1.113421618
EEF1E1	-1.113421618
CLDN9	-1.113421618
B3GAT1	-1.113421618
4732477C12RIK	-1.113421618
FNBP1	-1.113421618
9530077C05RIK	-1.113421618
LOC100045403	-1.113218667
TPBG	-1.112934309
6330509M05RIK	-1.112650121
ENTPD6	-1.112650121
POGK	-1.112650121
HSD11B1	-1.112650121
AHI1	-1.112650121
6330514A18RIK	-1.112650121
BC006909	-1.112650121
LOC381113	-1.112650121
C230043G09RIK	-1.112650121
AI839735	-1.112650121
ATPAF2	-1.112650121
UBE3C	-1.112650121
CTSA	-1.112650121
SMAP2	-1.112165649
BMP1	-1.112067224
ZNF276	-1.111879158
STIM2	-1.111879158
MFN2	-1.111879158

HSPE1	1.136816973
ARBP	1.136816973
PDGFA	1.136816973
TAC4	1.136816973
DDIT4L	1.137141645
2310047L21RIK	1.137605228
PDLIM2	1.137605228
CAMK2D	1.137605228
9330179O15RIK	1.137605228
NIT2	1.137605228
4732474O15RIK	1.137605228
NDUFA6	1.137896464
TGM2	1.138227533
LOC100039532	1.138329354
TIMP3	1.138391439
E030025L21RIK	1.138394029
CCL27	1.138394029
TAP2	1.138394029
LOC381750	1.138394029
VKORC1	1.138394029
LOC269542	1.138394029
2810017I02RIK	1.138394029
RDH10	1.138394029
BB049667	1.138394029
3110041P15RIK	1.138394029
BC012256	1.138394029
CENTD1	1.138394029
DAP	1.138394029
CDC42EP5	1.138394029
SAP30	1.138474469
LY6A	1.138705653
LOC100045542	1.13913748
A030009H04RIK	1.139183377
ITPR1	1.139183377
3222401M22RIK	1.139183377
SRPX2	1.139183377
PCCB	1.139183377
NOD1	1.139183377
PTGDS	1.139288834
TOR1B	1.139973273
IDB3	1.139973273
EDG2	1.139973273
RBM3	1.139973273
LRRC28	1.139973273
EVC2	1.139973273

ENDOD1	-1.261973008	CYP1B1	-1.111879158	JAM2	1.139973273
TTC9B	-1.260578335	DOS	-1.111879158	2410003B16RIK	1.139973273
KLC1	-1.25962998	ASAH1	-1.111879158	LOC100041388	1.139974851
FGF8	-1.25962998	GALNTL1	-1.111879158	LOC100046207	1.140021956
NRN1	-1.258757174	2310051E17RIK	-1.111879158	B930075F07	1.140197042
SLC17A6	-1.258757174	DNAJC7	-1.111613847	PPM1B	1.140763716
GPR158	-1.257013375	PRKCSH	-1.111569587	C030003A18RIK	1.140763716
RTN2	-1.257013375	DDX17	-1.11129165	HIST1H2BK	1.140763716
PLP1	-1.25696309	A830020B06RIK	-1.111108729	UBE1L	1.140763716
SCN1B	-1.255271991	LCE-PENDING	-1.111108729	2610009I02RIK	1.140763716
CNTN2	-1.255271991	2900075A18RIK	-1.111108729	4632401N01RIK	1.140763716
USP6NL	-1.254402205	ADAMTS20	-1.111108729	LYZ	1.14082763
HCN2	-1.254402205	2410012H22RIK	-1.111108729	COPG2AS2	1.140965024
A230098E07RIK	-1.254402205	B230114J08RIK	-1.111108729	1110059E24RIK	1.14142052
IL16	-1.25353302	D030056L22	-1.111108729	SNX30	1.141513811
F2RL1	-1.252664439	GBA2	-1.111108729	5730593F17RIK	1.141554707
6430702L12	-1.252664439	1110067I12RIK	-1.111108729	2410012C07RIK	1.141554707
ACTA1	-1.252664439	GCG	-1.111108729	GNB1L	1.141554707
SCG5	-1.252383218	FNTA	-1.111108729	NFIB	1.141554707
ST6GAL1	-1.251796459	PHF20	-1.110831569	ST6GALNAC4	1.141554707
ADAMTS19	-1.251796459	PARVA	-1.110707851	SCRG1	1.141554707
EGF	-1.249196126	HAK-PENDING	-1.110593386	SUCLG2	1.142346247
BSG	-1.248330549	SCD	-1.110338834	TFF2	1.142346247
PEA15	-1.248330549	RHOBTB3	-1.110338834	CNTNAP2	1.142346247
MPZ	-1.248330549	LOC211556	-1.110338834	CRIP2	1.142346247
6720460L05RIK	-1.247465572	USP20	-1.110338834	2410146L05RIK	1.142346247
FRMPD1	-1.245737416	C230098O21RIK	-1.110338834	D16BWG1494E	1.142529343
GPR37L1	-1.245737416	6230400G14RIK	-1.110338834	LOC381230	1.142821837
SLITRK5	-1.244874235	BST2	-1.110338834	SH3RF1	1.143138335
SULT4A1	-1.244874235	AP4S1	-1.110338834	0610009L18RIK	1.143138335
HSPB3	-1.244874235	MCOLN3	-1.110338834	ITPR3	1.143138335
FGF12	-1.244011653	MAFG	-1.109711528	2510006D16RIK	1.143138335
APLP1	-1.242288282	ACSL4	-1.109569472	LOC215098	1.143138335
IGFBPL1	-1.242109113	SPAST	-1.109569472	OTOR	1.143138335
SYNGR3	-1.241427492	EPS15	-1.109569472	CHST10	1.143138335
FSCN2	-1.240064588	ZBTB46	-1.109569472	FMOD	1.143138335
CACNA1D	-1.238237946	FAAH	-1.109569472	C130074G19RIK	1.143138335
MAL	-1.237990291	GPX6	-1.109569472	LOC383131	1.143138335
LRP11	-1.237990291	E130310K16RIK	-1.109569472	ZFHX4	1.143138335
TRYGN16	-1.237990291	PI4K2A	-1.109569472	SPNB2	1.143478642
TGFBI	-1.236275261	AI661919	-1.109569472	YBX3	1.143930973
2900026H06RIK	-1.236275261	SLC14A1	-1.109569472	GALT	1.143930973
D430044G18RIK	-1.235418637	XLR3A	-1.109569472	TEMT	1.143930973
SFRS14	-1.234562607	2810441K11RIK	-1.109569472	FKBP11	1.143930973
D5BWG0834E	-1.234562607	SOBP	-1.109349631	RPL12	1.143930973

CNP	-1.234562607
D12ERTD553E	-1.23370717
3830431G21RIK	-1.23370717
EGR1	-1.232852325
EG245190	-1.232832374
ITGA8	-1.231998073
IFITM1	-1.231998073
ESRRG	-1.231664427
3110035E14RIK	-1.231144413
RAB7A	-1.231144413
1110012N22RIK	-1.231144413
E330013P04RIK	-1.229438867
LOC100045304	-1.229385591
SLC9A6	-1.22858698
CHORDC1	-1.22858698
2010317E24RIK	-1.22858698
ZFP537	-1.226884977
CLDN1	-1.226884977
ATP2A3	-1.22664845
DRP2	-1.22603486
HMGCS1	-1.22603486
4933407M15RIK	-1.225185332
RAB6B	-1.225185332
1810029C22RIK	-1.225185332
AGPAT3	-1.225185332
IQSEC3	-1.224336392
SLC34A3	-1.224336392
SYN2	-1.224336392
LOC382924	-1.224336392
ZFP536	-1.222640278
KRTDAP	-1.222640278
SEPT6	-1.221793102
TANK	-1.221793102
CDKN1B	-1.220946513
RGS7	-1.220946513
0610009A07RIK	-1.220946513
GM414	-1.220161793
6330403K07RIK	-1.22010051
5730593N15RIK	-1.22010051
REEP6	-1.219255094
LOC237436	-1.219255094
GPR120	-1.218900738
A630056B16RIK	-1.218410264
2410129E14RIK	-1.218410264
C630013B14RIK	-1.218410264

USP47	-1.109284744
ATP5G3	-1.109178637
C030014L02	-1.108800644
LAPTM4B	-1.108800644
BC051083	-1.108800644
HTATIP2	-1.108800644
2410195B05RIK	-1.108800644
PPM1E	-1.108800644
LAMP2	-1.108800644
TUBA4A	-1.108800644
AQP4	-1.108800644
DOCK9	-1.108800644
KIF5A	-1.108535291
PUNC	-1.108416715
5930418K15RIK	-1.108032348
1500011B03RIK	-1.108032348
ZDHHC7	-1.108032348
CNTNAP4	-1.108032348
A230102O09RIK	-1.108032348
RUTBC2	-1.108032348
PGRMC2	-1.108032348
RTN4RL1	-1.108032348
2410025L10RIK	-1.108032348
LOC245670	-1.108032348
AU040320	-1.108032348
6330414G02RIK	-1.108032348
MLF2	-1.10802079
SIX1	-1.107532553
MYO5B	-1.107307767
BC061928	-1.107264584
1110014O20RIK	-1.107264584
HACE1	-1.107264584
INPP5F	-1.107264584
ATP6V0A1	-1.107264584
B230341H15RIK	-1.107264584
LOC380694	-1.107264584
SCAMP1	-1.107264584
DFFA	-1.107264584
HK1	-1.107264584
6430550H21RIK	-1.107264584
HERC3	-1.107264584
KREMEN1	-1.107264584
ZFC3H1	-1.107037914
CXCL14	-1.106594187
PCBP4	-1.106497353

NOTCH4	1.143930973
MT-ND4L	1.143930973
TGFBR1	1.143975549
GPR83	1.144002397
SESN1	1.144169073
DOK3	1.144724161
DCI	1.144724161
HS3ST3A1	1.144724161
POLE3	1.144724161
1600020H07RIK	1.145517898
BC055107	1.145517898
LOC100036521	1.145664207
2410006H16RIK	1.14593261
LOC100046775	1.146119067
LYRM2	1.146144128
C22	1.146312186
ANGPT1	1.146312186
CRTAP	1.146312186
CDCA7	1.146312186
PDCD2	1.146312186
CLRN1	1.146312186
SOX18	1.146312186
BC042720	1.147107024
NETO2	1.147107024
APRT	1.147107024
AU040950	1.147107024
CHI3L1	1.147107024
1110002B05RIK	1.147900311
NME4	1.147902414
ALAS2	1.147902414
B230343H07RIK	1.147902414
SLC39A11	1.148698355
RPL30	1.148698355
D11ERTD603E	1.148698355
CD109	1.149494848
LTF	1.149494848
2010300G19RIK	1.149494848
PCOLCE	1.149494848
EIF2S3Y	1.150034253
MATN1	1.150291893
1810073P09RIK	1.150291893
ABCD1	1.150291893
NOXO1	1.150291893
2210410E06RIK	1.150291893
CMTM7	1.150579013

CKMT1A	-1.218410264
RDH12	-1.218410264
2610034M16RIK	-1.217768102
4732474A20RIK	-1.217566019
PBX2	-1.217566019
GNG8	-1.216483872
RASD1	-1.215879283
CFL2	-1.215879283
UGT8	-1.215879283
LOC381140	-1.215036792
CLIC6	-1.214371714
B230206H07RIK	-1.214194884
TUBA4B	-1.21335356
EDNRB	-1.21335356
2810407C02RIK	-1.212512819
INA	-1.212512819
D6ERTD245E	-1.21167266
MYH8	-1.21167266
DLX2	-1.21167266
CCL11	-1.210833084
SCIN	-1.210833084
UCP1	-1.210833084
B230343A10RIK	-1.209994089
KIF1B	-1.209994089
2210407P13RIK	-1.209994089
JMJD1A	-1.209155676
2010110P09RIK	-1.209155676
AU040576	-1.209155676
NLK	-1.209155676
PGAM2	-1.207867204
THY1	-1.207480591
CBLN4	-1.207480591
COL18A1	-1.207480591
MLLT11	-1.207178154
XIST	-1.206733542
ESPN	-1.206267558
2310043N10RIK	-1.205807828
9130422H11RIK	-1.205807828
OTX2	-1.204972315
4732437J24RIK	-1.204972315
7530422H14RIK	-1.204972315
KIF21A	-1.204972315
TTYH2	-1.204137381
AMPH	-1.204137381
NNAT	-1.204137381
4631427C17RIK	-1.106497353
SCN3B	-1.106497353
CLDN14	-1.106497353
PITPNA	-1.106497353
AW061290	-1.106497353
LGI4	-1.106497353
5031439G07RIK	-1.106497353
3110068G20RIK	-1.106497353
PHTF2	-1.106497353
0610031J06RIK	-1.106497353
SH3GL3	-1.106497353
ATP6AP2	-1.106497353
1600021P15RIK	-1.106497353
OTOG	-1.106224152
CACNB3	-1.106068772
RAB28	-1.105730653
LOC330267	-1.105730653
2900072M03RIK	-1.105730653
0610006O14RIK	-1.105730653
2700050C12RIK	-1.105730653
LIN7A	-1.105730653
SEPW1	-1.105730653
5430406J06RIK	-1.105730653
B830009D23RIK	-1.105730653
PPP1R16A	-1.105730653
VDAC1	-1.105575539
CAR12	-1.105457814
SPIRE2	-1.105372617
CDKN1A	-1.105234017
CTGF	-1.105085734
SCL000959.1_2	-1.10499881
ARNT	-1.104964485
B230205M03	-1.104964485
CLTB	-1.104964485
MAPK10	-1.104964485
B230106I24RIK	-1.104964485
CDKN2D	-1.104964485
SOD1	-1.104766441
GH	-1.104683335
CAR9	-1.104297345
CNTN1	-1.104266888
4930429O20RIK	-1.104198847
BC033596	-1.104198847
MAPK8IP2	-1.104198847
NHLRC2	-1.104198847
4930533K18RIK	1.151089491
IL18	1.151089491
GLI2	1.151887642
NEURL	1.151887642
PRKG1	1.151887642
LOC280408	1.151887642
FIN14	1.151887642
SLC4A1	1.152686347
EMD	1.152686347
IL11RA2	1.152686347
PSMB1	1.153430055
1110007F05RIK	1.153485605
GSTT1	1.153485605
MFAP4	1.153485605
BC037006	1.153485605
TNNT2	1.153485605
GPNMB	1.153485605
2310076G05RIK	1.153485605
9030013K10RIK	1.153485605
FOXC2	1.153485605
ENTPD2	1.154285418
1110001C20RIK	1.154285418
1700031F13RIK	1.154285418
TUBA1C	1.154285418
4930528G09RIK	1.154285418
RETNLG	1.154285418
ATXN1	1.155050939
TCF19	1.155085785
GNA11	1.155085785
MTHFD2	1.155085785
HTRA1	1.155085785
SCAMP3	1.155085785
ARHGDIG	1.155347131
OSCAR	1.155886707
LOC386192	1.155886707
CHST5	1.155886707
0610007N19RIK	1.155886707
LOC383883	1.155886707
2900093B09RIK	1.155886707
PNRC2	1.155886707
CLDN5	1.155886707
4931433A13RIK	1.155886707
DDX6	1.155899485
NXPH1	1.156640252
2700094K13RIK	1.156688184

TM4SF5	-1.204137381	EYA4	-1.104198847	LGALS3	1.156688184
C130060K24RIK	-1.204137381	5430407P10RIK	-1.104198847	COL5A2	1.156688184
FGFRL1	-1.204137381	IDH3B	-1.104198847	1700006D24RIK	1.156688184
P2RX2	-1.202469249	AKAP11	-1.104198847	ORMDL3	1.156688184
HSP90B1	-1.202213649	RNF6	-1.104198847	RABL4	1.156688184
GAD1	-1.20163605	HSPA8	-1.104146909	LOC100039346	1.156800348
LOC381037	-1.20163605	5430405G05RIK	-1.103832007	MXD4	1.157490217
PTPRT	-1.20163605	NCSTN	-1.103789343	4632428N05RIK	1.157490217
MYD116	-1.20163605	SEC11A	-1.103518318	PCCA	1.157490217
DDN	-1.20163605	C130020C13RIK	-1.10343374	AWP1-PENDING	1.157490217
RAB3IP	-1.20163605	FOXG1	-1.10343374	HSPB8	1.157625486
PDZK1IP1	-1.20146295	CYP51A1	-1.10343374	PGM5	1.158292806
LYNX1	-1.200803427	KTN1	-1.10343374	A330048O09RIK	1.158292806
SCNN1B	-1.200803427	STX1A	-1.10343374	SCN11A	1.158292806
PIGT	-1.200803427	BC040774	-1.10343374	SEMA4A	1.158292806
SIX2	-1.199971382	D030001E08	-1.10343374	E2F5	1.158462211
6430595O10RIK	-1.199971382	PPT2	-1.10343374	MAP3K3	1.158790049
USP2	-1.199971382	ZFPM2	-1.103133936	PON2	1.159257285
SLC4A7	-1.199971382	KLHL17	-1.102669163	H2-T23	1.159278427
PCDH7	-1.198309021	D13BWG1146E	-1.102669163	MEIS2	1.159409876
NF2	-1.198309021	PRKAA1	-1.102669163	TPM1	1.159899655
SYT1	-1.198309021	GPR137B	-1.102669163	CDCA3	1.159899655
POSTN	-1.196648963	BTBD3	-1.102669163	ASGR1	1.159899655
C630022N07RIK	-1.196648963	D430019H16RIK	-1.102669163	GSTT3	1.159899655
NCAM1	-1.195819797	MVK	-1.102669163	GSC	1.160462555
DPP4	-1.195819797	UNC5C	-1.102669163	1200013A08RIK	1.160703914
CHST8	-1.195819797	CDC20	-1.102587082	SLIT2	1.160703914
FMR1	-1.194991205	GAA	-1.102530454	TXNL2	1.160703914
BC026370	-1.194991205	LLGL2	-1.102246239	ANTXR2	1.160703914
A830080H07RIK	-1.194991205	ZDHHC13	-1.102048842	SNHG10	1.160919327
EPYC	-1.194549897	SNN	-1.101905116	1110031K21RIK	1.161508732
GGTL3	-1.194163187	TMPRSS3	-1.101905116	ELFN2	1.161508732
4833418A01RIK	-1.194163187	LOC385454	-1.101905116	1700129I04RIK	1.161655547
1700020I14RIK	-1.194163187	LOC280487	-1.101905116	IGK-C	1.162314108
RPRM	-1.193335743	ARL8A	-1.101905116	SNX10	1.162314108
FGF1	-1.193335743	KCNA2	-1.101905116	MPO	1.162314108
C630015F21RIK	-1.192508872	SEPT9	-1.101905116	B230209E15RIK	1.162374126
UPP1	-1.192508872	GNAO1	-1.101905116	1810014L12RIK	1.163120042
PELI2	-1.192508872	GTF3C2	-1.101905116	SLCO1A4	1.163120042
RYR2	-1.191682575	AKT3	-1.101905116	LOC381229	1.163120042
PCDH17	-1.191650069	1200016B17RIK	-1.101905116	C330027I04RIK	1.163926534
ENO2	-1.191412357	NEK4	-1.101905116	LMO3	1.163926534
D6WSU176E	-1.191189329	CDK10	-1.101905116	5830411I20	1.163926534
HPCA	-1.190856849	SLC8A1	-1.101905116	CIB2	1.163926534
ABHD4	-1.190856849	REV3L	-1.101905116	MRPS16	1.163926534

ARHGAP20	-1.190031696	D6ERTD538E	-1.101905116	SMO	1.163926534
N28178	-1.190031696	DNM1	-1.101905116	CPXM1	1.163926534
GM851	-1.189711825	ARHGEF10	-1.101905116	GPR155	1.164239737
TOMM34	-1.189207115	CPSF1	-1.101905116	2310039H08RIK	1.164733586
OLFML2A	-1.189207115	ERCC3	-1.101838259	KIT	1.164733586
LOC381795	-1.189207115	LSM1	-1.101656373	D130058I21RIK	1.164733586
ATP5J	-1.189049831	PSME3	-1.101558814	BC018222	1.164733586
UBQLN1	-1.188773585	SMG7	-1.101490303	SNX7	1.164733586
DUSP4	-1.188383105	RASL11B	-1.101328511	GOLGA2	1.165541198
C030013G03RIK	-1.188383105	SUI1-RS1	-1.101245747	TTYH3	1.165541198
6820402O20RIK	-1.188383105	1110059G02RIK	-1.101141598	HIST1H2AN	1.165541198
EMID1	-1.188383105	NUPL2	-1.101141598	OLIG1	1.165602778
RGS17	-1.188383105	4933424C13RIK	-1.101141598	S100B	1.16634937
WIPF3	-1.188355894	XCL1	-1.101141598	RPL4	1.16634937
EG244911	-1.188243703	2610024B07RIK	-1.101141598	NRARP	1.16634937
TMC1	-1.18771812	PLEKHB1	-1.101141598	CPD	1.16634937
C130020C07RIK	-1.187559666	SC4MOL	-1.101141598	LOC233529	1.16634937
2610034E13RIK	-1.187559666	TXNDC15	-1.100878267	TCFCP2L1	1.16634937
SYN1	-1.187559666	2610304F08RIK	-1.100754808	OLFML1	1.16634937
PDIA2	-1.187559666	ALG2	-1.100686299	AW536289	1.16634937
A730095J18RIK	-1.187559666	PCDHA7	-1.100392722	S100A9	1.166540781
CAMK4	-1.186936137	RNF44	-1.100378609	GTF2I	1.167158102
0610033M10RIK	-1.186736798	E330036I19RIK	-1.100378609	LOC382885	1.167158102
9530056K15RIK	-1.186736798	BEX2	-1.100378609	2210008A03RIK	1.167158102
1810057C19RIK	-1.186736798	ARHGAP1	-1.100378609	LRRN2	1.167967395
CTSF	-1.186736798	AW544865	-1.100378609	BC022765	1.167967395
1110017D07RIK	-1.185914499	STK39	-1.100378609	EMP1	1.167967395
ACCN3	-1.185914499	ABLIM2	-1.100378609	HSP90AB1	1.167967395
PCMT1	-1.185914499	GAMT	-1.100378609	IGF1	1.168408888
SCN1A	-1.185914499	STK10	-1.100378609	BMP8A	1.168777249
RAB7	-1.185138438	4930541M15RIK	-1.100378609	RHOJ	1.168777249
CCDC3	-1.185092771	MGST3	-1.100378609	LRIG1	1.168777249
2700031B12RIK	-1.185092771	TSC2	-1.100375761	PMM1	1.169587664
CNDP2	-1.185092771	OSR2	1.100122124	9330169N05RIK	1.169587664
1700041B20RIK	-1.185092771	LOC674611	1.100170094	RGS2	1.169587664
2310004N11RIK	-1.184271612	PLCH2	1.100297145	RAB32	1.169587664
SIAE	-1.184271612	ESAM	1.100315523	CARS	1.170398641
4921509J17RIK	-1.184271612	CLPS	1.100349602	LCN2	1.170398641
ACTR1B	-1.183451022	MGC18837	1.100378609	SLC16A13	1.170398641
9630007J19RIK	-1.183451022	DHFR	1.100378609	RBP4	1.170398641
SLC6A15	-1.182631	BMP6	1.100378609	4833413D08RIK	1.17061113
TBC1D7	-1.182631	1810061M12RIK	1.100378609	TEKT1	1.171210181
PROX1	-1.182631	LOC385923	1.100378609	HIST3H2BA	1.171210181
NPHP1	-1.182631	LOC240906	1.100378609	BC017612	1.172022284
HS6ST2	-1.182631	LOC380771	1.100378609	PPP1R3C	1.172022284

PCSK1N	-1.181811547	D4ERTD765E	1.100378609	2610111M03RIK	1.172022284
C530008M17RIK	-1.181811547	CD84	1.100378609	1110021J02RIK	1.172834949
A130092J06RIK	-1.181811547	2810453I06RIK	1.100378609	KLF6	1.172880219
NTNG1	-1.181570655	GARNL3	1.100378609	3110032G18RIK	1.173648178
ARG2	-1.180992661	ISLR	1.100378609	LOC225456	1.173648178
MKIAA0282	-1.180992661	BC006933	1.100378609	SFXN5	1.173648178
KCNIP3	-1.180992661	LEPRE1	1.100378609	GSTM2	1.173648178
GPR126	-1.180174343	IFITM6	1.100378609	GSTM1	1.173648178
2310001H13RIK	-1.180174343	TACSTD2	1.100378609	BCAN	1.174461971
KCNK2	-1.180174343	AI646023	1.100378609	5033405K12RIK	1.174461971
CYP2J9	-1.180174343	2610010A15RIK	1.100378609	KRT1-19	1.174461971
D030028O16RIK	-1.180174343	KCNJ15	1.100378609	CCL21B	1.174461971
COL9A2	-1.17957694	RAI14	1.100378609	RGS5	1.174957614
ACSL1	-1.179356592	4933434I06RIK	1.100378609	GNB4	1.175276328
HIBADH	-1.179356592	2810410A03RIK	1.10048686	IRS1	1.175276328
SCHIP1	-1.179356592	2610020O08RIK	1.100509917	GPX1	1.175276328
FOXQ1	-1.179356592	RAD23A	1.100604117	4933428D01RIK	1.175276328
VSNL1	-1.179001374	SLITRK4	1.100607334	RSPH1	1.175442634
9130229H14RIK	-1.178539408	2010004M13RIK	1.100617342	SOX9	1.175554545
RGS11	-1.178539408	GPRC5B	1.100778319	LOC433955	1.175888914
DUSP1	-1.178539408	4931408A02RIK	1.100790625	LOC381681	1.17609125
4930572J05RIK	-1.178469476	ZFP277	1.100813032	1700081H05RIK	1.17609125
TNC	-1.178407198	KRTCAP3	1.100931707	COL6A1	1.17609125
TSGA14	-1.177815704	ARPC5L	1.100953192	4933412E12RIK	1.176906737
TMEM30B	-1.17772279	GAS5	1.101141598	ACP5	1.176906737
LRRIQ2	-1.17772279	1110012M11RIK	1.101141598	1110061A14RIK	1.176906737
PXMP2	-1.176906737	1700127F16RIK	1.101141598	IL17RE	1.176906737
KCNIP1	-1.176906737	NSD1	1.101141598	KRT2-8	1.17772279
ANGPT2	-1.176906737	ANK3	1.101141598	HOXA2	1.178539408
DAG1	-1.176906737	COL4A5	1.101141598	IGFBP4	1.178539408
CREB5	-1.176906737	RASSF2	1.101141598	LOC100047173	1.178929708
FADS1	-1.176906737	WBP11	1.101141598	DCTN1	1.179356592
OLFML3	-1.176774128	BC013491	1.101141598	1700003M02RIK	1.179356592
GFRA1	-1.17609125	PLEKHM1	1.101141598	EG668850	1.180036125
CRG-L1	-1.17609125	ASPH	1.101141598	COPZ2	1.180174343
ITGA6	-1.17609125	EDG7	1.101141598	RPL31	1.180174343
UTRN	-1.17609125	6030443O07RIK	1.101141598	MLANA	1.180174343
ABCA3	-1.17609125	1700112N14RIK	1.101141598	6030411F23RIK	1.180174343
KIF3A	-1.175276328	CDKL2	1.101141598	TNS1	1.180174343
VTN	-1.175276328	ATAD4	1.101276993	LRCH2	1.180174343
LDLR	-1.175276328	1810058M03RIK	1.10165865	SAMD9L	1.180430195
HMGCS2	-1.175276328	NSBP1	1.101905116	ACOT7	1.180992661
C030003D03RIK	-1.175276328	2810474O19RIK	1.101905116	RIMS3	1.180992661
PTPLAD1	-1.175218922	B4GALT7	1.101905116	ENAH	1.181811547
PDIA5	-1.174462781	EPHB4	1.101905116	LOC100044177	1.18188369

E030041M21RIK	-1.174461971	MIF	1.101905116	PTHR1	1.182631
COL23A1	-1.174461971	SNTA1	1.101905116	KCNQ1	1.182631
TAGLN3	-1.173880093	LOC330844	1.101905116	KIF9	1.182631
VAMP1	-1.173648178	TXNDC1	1.101905116	SORCS3	1.182631
5730437P09RIK	-1.172834949	CTSZ	1.101905116	TNS3	1.182813977
GATA2	-1.172834949	EPHA4	1.101905116	A430091O22RIK	1.183451022
ACE	-1.172834949	SRR	1.101994987	IGFALS	1.183451022
PCSK2	-1.172834949	CAR11	1.102001051	CRYM	1.183451022
1200009O22RIK	-1.172834949	RPS16	1.10220634	ZFP185	1.183451022
WASF1	-1.172834949	MPV17L	1.102440444	ENSMUSG00000068790	1.184007487
KRAS	-1.172834949	C330006A16RIK	1.102450719	ECEL1	1.184510152
BC057371	-1.172834949	NME3	1.102610125	6430537F04	1.184707189
WDR7	-1.172834949	LOC383775	1.102669163	PRSS22	1.185092771
GM22	-1.172224649	COL6A2	1.102669163	MYL9	1.185092771
CAMTA1	-1.172022284	0610037P05RIK	1.102669163	CRYGN	1.185092771
C230095G01RIK	-1.172022284	NDUFB3	1.102669163	BC025206	1.185092771
E430030L01RIK	-1.172022284	TMEM5	1.102669163	BC043944	1.185092771
HIC1	-1.171210181	HM13	1.102669163	6330503K22RIK	1.186532539
ELOVL4	-1.171210181	MEOX2	1.102669163	FBXO36	1.186736798
METRN	-1.170730311	EPPB9	1.102669163	2310015B20RIK	1.187559666
AADACL1	-1.170685159	UPK2	1.102669163	TRPM1	1.187559666
KIF21B	-1.170398641	RNASE4	1.102713426	LOC244710	1.187559666
OTUB2	-1.170398641	MAPK13	1.102790416	PDK4	1.188383105
HEY1	-1.170398641	CADPS2	1.103152477	DLL3	1.188383105
LGI2	-1.170210091	EXOSC8	1.10343374	LOXL2	1.188383105
5830445O15RIK	-1.169587664	2210408I21RIK	1.10343374	1700055O19RIK	1.189207115
B230209C24RIK	-1.169587664	A630080F05RIK	1.10343374	DMP1	1.189207115
MCFD2	-1.169496576	5133401H06RIK	1.10343374	PYGL	1.189643897
PPARGC1B	-1.168777249	RBL1	1.10343374	LOC384710	1.190031696
LGMN	-1.168777249	LOC381860	1.10343374	BC035954	1.190031696
SCGB3A1	-1.168777249	FRZB	1.10343374	PDGFB	1.190031696
SOX11	-1.168777249	SVEP1	1.10343374	POLK	1.190856849
CHST1	-1.167967395	CTDSP1	1.10343374	DPP8	1.190856849
DTX3	-1.167967395	EIF3S2	1.10343374	CDKN1C	1.190856849
MYCBP	-1.167967395	GRB7	1.10343374	POU3F4	1.191009018
ABCA8A	-1.167967395	BC006662	1.10343374	BC028975	1.191682575
BQ952480	-1.167967395	BCCIP	1.10343374	RAMP1	1.191682575
MRPS26	-1.167967395	HIST2H3C1	1.103582233	0610005K03RIK	1.191682575
STMN3	-1.167398967	MFGE8	1.103603856	GREM1	1.191682575
9630058J23RIK	-1.167158102	LOC546015	1.103639249	EEF2	1.192131414
WNT7A	-1.166569765	RPS13	1.103648269	INSM1	1.192508872
OSBPL3	-1.16634937	LOC623568	1.103720714	CHRNB1	1.192508872
KCNMB4	-1.165541198	ELOF1	1.103934104	LOC386169	1.193335743
SLC26A7	-1.165541198	DCPP1	1.103964719	BTBD11	1.193394355
DSCAM	-1.165541198	MAP2K1	1.104198847	CITED4	1.193721544

ODD1	-1.165541198
PCDHA1	-1.165541198
GLUD1	-1.165541198
2310037P21RIK	-1.165541198
LEPROTL1	-1.165541198
PLOD3	-1.165535268
LUM	-1.165071738
ENPP2	-1.164733586
A130028J20RIK	-1.164733586
SYNJ2	-1.164733586
C130078N17RIK	-1.164733586
B230312L03RIK	-1.163926534
AU016693	-1.163926534
INSL3	-1.163926534
A930017G19RIK	-1.163120042
2600009P04RIK	-1.163120042
4632417K02	-1.163120042
SEMA5B	-1.163120042
BC050254	-1.162314108
GGT7	-1.162034066
GCNT2	-1.161877052
FIBCD1	-1.161508732
PNLIPRP1	-1.161508732
KLC2	-1.161508732
CACNA1H	-1.161508732
5930434B04RIK	-1.161508732
COL2A1	-1.161124942
BC048546	-1.160950588
RAP1GAP	-1.160703914
MAP6	-1.160703914
OSTF1	-1.160703914
RAB9B	-1.160703914
MRPS25	-1.160703914
SLC29A1	-1.160506361
2610511M17RIK	-1.159899655
LOC245020	-1.159899655
E230024B12RIK	-1.159899655
IGSF4A	-1.159411701
GANC	-1.159405465
REEP1	-1.159351295
CRY2	-1.159095952
SLC39A6	-1.159095952
DEFB5	-1.159095952
ABHD3	-1.159095952
PLEKHA2	-1.159095952

1810018P12RIK	1.104198847
A230072I16RIK	1.104198847
7530403E16RIK	1.104198847
LOC226864	1.104198847
425O18-1	1.104198847
CCND3	1.104198847
COL4A6	1.104198847
UHRF1	1.104198847
MRPS18C	1.104198847
SLC12A6	1.104198847
CTNNBL1	1.104198847
PROKR1	1.104198847
4432416O06RIK	1.104198847
CDO1	1.104198847
LOC381850	1.104198847
BSND	1.104223953
MYEOV2	1.104475704
DIRAS2	1.104657957
GJB2	1.104964485
AU020206	1.104964485
MYO1B	1.104964485
AMN	1.104964485
RAP2B	1.104964485
SFRS10	1.104964485
HIST1H2AK	1.104964485
D030029J20RIK	1.104964485
NUP93	1.104964485
GUCY1A3	1.104964485
MBNL1	1.104964485
PGLYRP1	1.105020227
1700088E04RIK	1.105056317
QARS	1.105074166
SCARA5	1.105088577
SCARF2	1.105437696
SIVA1	1.105471255
SYPL	1.105476571
LYZ2	1.105678068
CD47	1.105698417
AMD1	1.105730653
DDT	1.105730653
6030404G09RIK	1.105730653
CLDN7	1.105730653
LRP12	1.105730653
9430088P09RIK	1.105730653
CNN2	1.105730653

ABAT	1.194163187
PRPH2	1.194163187
RALYL	1.194712916
HES5	1.194991205
4921528H16RIK	1.194991205
EPB4.1L4B	1.194991205
A730024G14RIK	1.194991205
BC044804	1.194991205
LOC332300	1.194991205
A430096B05RIK	1.194991205
EXT1	1.195819797
SMOC1	1.195819797
4933417K04RIK	1.195819797
SLC2A13	1.196095022
EFNA1	1.196648963
ADCY6	1.196648963
DSG2	1.197478705
2810022L02RIK	1.197478705
C1QTNF4	1.197478705
SST	1.19755555
CARD10	1.198309021
IPO11	1.199139914
2610028F08RIK	1.199971382
IQGAP2	1.200797815
LBP	1.200803427
RIN3	1.200803427
TNFRSF11B	1.200803427
GADD45G	1.200953121
PPP1CA	1.201015572
9130213B05RIK	1.201240855
ST8SIA2	1.20163605
ARHGEF3	1.20163605
LOC270152	1.20163605
RSHL3	1.20163605
ELN	1.202469249
LOC386534	1.202469249
1500012F01RIK	1.202700105
TXNDC13	1.203204169
APOA4	1.203303026
PNPLA8	1.203697047
HAP1	1.203775154
2700069I18RIK	1.204137381
CHST7	1.204137381
1200013B22RIK	1.204972315
PHF10	1.204972315

PPM1J	-1.159095952
2810405F04RIK	-1.159095952
TMEM199	-1.158545446
ADIPOR2	-1.158542758
1700052O22RIK	-1.158292806
PKD1L2	-1.158292806
2210408F21RIK	-1.158292806
FGF10	-1.158292806
GNG2	-1.158292806
ENTPD3	-1.158292806
CNTN5	-1.158193268
DUSP6	-1.157602593
SH3GL2	-1.157490217
PRKAR2A	-1.157490217
CDS1	-1.156688184
NDFIP2	-1.156688184
SHOX2	-1.156688184
WBSCR17	-1.156688184
NAPB	-1.156688184
AI593442	-1.156688184
CALML4	-1.156688184
PDIA6	-1.156688184
ABCG5	-1.156366898
RUFY3	-1.156233822
GM644	-1.155886707
D11ERTD497E	-1.155886707
5730592L21RIK	-1.155886707
LOC100044566	-1.155814049
NEF3	-1.155448322
SLC4A2	-1.15512784
C130090K23RIK	-1.155085785
S100A1	-1.155085785
WSB2	-1.155085785
GDF1	-1.155085785
TMC7	-1.155085785
3010021M21RIK	-1.154285418
APOD	-1.154285418
8030451F13RIK	-1.154285418
RIT2	-1.153485605
SHH	-1.153485605
LIG1	-1.153485605
MARVELD3	-1.153485605
KCNA6	-1.153368256
MMP13	-1.152734234
ABCG1	-1.152686347

NFIA	1.105730653
1700113I22RIK	1.105730653
AGPAT2	1.105730653
LOXL4	1.105730653
MRPS6	1.105730653
6330406I15RIK	1.105823539
RPS9	1.106204699
LOC380863	1.106497353
8430410K20RIK	1.106497353
1300017K07RIK	1.106497353
4933424B01RIK	1.106497353
SUOX	1.106497353
SLC10A3	1.106497353
2410030K01RIK	1.106497353
XPA	1.106497353
LXN	1.106497353
6430573F11RIK	1.106497353
BAI2	1.106497353
D030063E12	1.106881625
2610524G07RIK	1.107264584
FLT1	1.107264584
INSIG2	1.107264584
RPL14	1.107264584
VASN	1.107264584
ETOHD2	1.107264584
TSPAN12	1.107264584
2900001O04RIK	1.107264584
XRN2	1.107264584
STARD4	1.107264584
SH3BGRL	1.107264584
JAG2	1.107264584
1110038B12RIK	1.107264584
D130084M03RIK	1.107264584
DBNDD2	1.107418794
RPL9	1.10760672
UNC84B	1.107932433
9430028L06RIK	1.107956254
BC009118	1.108032348
1110020C03RIK	1.108032348
BC025600	1.108032348
2610036L11RIK	1.108032348
LOC232745	1.108032348
2310061F22RIK	1.108032348
PSMC5	1.108032348
GNG13	1.108032348

BGN	1.205807828
1810041L15RIK	1.205807828
EG626367	1.205874309
RPL7A	1.20634994
6330404F12RIK	1.20664392
FKBP7	1.20664392
LOC386360	1.206956827
PCDH21	1.207480591
RARRES1	1.209107755
D5ERTD593E	1.209155676
4930403E08RIK	1.209155676
PARP1	1.209994089
6720430O15	1.209994089
BC055811	1.209994089
ALDH1A1	1.209994089
SYT4	1.210014853
DDAH1	1.210535049
MRPL52	1.210833084
TMEM20	1.210833084
4833422F24RIK	1.210833084
FBLN2	1.21167266
9930017A07RIK	1.21167266
CCND2	1.21167266
3830422K02RIK	1.212512819
4933405A16RIK	1.212512819
CACNA2D1	1.212940407
SERPINA3N	1.21335356
KDELR3	1.21335356
2410019A14RIK	1.214194884
TXNIP	1.214809071
AI505012	1.215036792
ZHX2	1.215879283
2310020A21RIK	1.215879283
2010308M01RIK	1.215879283
NID2	1.217566019
1700054E11RIK	1.218410264
A830030H10RIK	1.218410264
LOC100043192	1.218969004
CAR13	1.219255094
H6PD	1.22010051
RNF144	1.22010051
CHRNG	1.220726893
WISP1	1.220946513
1600029I14RIK	1.221296623
CASP2	1.221793102

LANCL1	-1.152686347	ANTXR1	1.108032348	CMTM5	1.223488041
A630028F16	-1.152686347	GNG11	1.108032348	A2M	1.223488041
1700018O18RIK	-1.152686347	PROSC	1.108032348	MGC41689	1.223488041
SOX2	-1.152686347	8430415N23RIK	1.108032348	SMPD3	1.223488041
D930029E11RIK	-1.152686347	TIAL1	1.108032348	H19	1.223746756
4930470O13RIK	-1.151887642	SH3BP2	1.108032348	COX7A1	1.224336392
MEGF9	-1.151887642	FRAP1	1.108032348	A930029B02RIK	1.224336392
C030025P15RIK	-1.151887642	NTRK3	1.108032348	MEPE	1.224336392
DLGH2	-1.151887642	WNK4	1.108032348	CELSR2	1.224905881
GALNT13	-1.151089491	4833421E05RIK	1.108032348	SLC32A1	1.224910076
1810073K19RIK	-1.151089491	SFRS6	1.108108454	CLDN11	1.225011965
LAMA2	-1.151089491	DLK1	1.108164421	FXYD5	1.225185332
MAN2B1	-1.151089491	RPL18	1.108202855	OTOS	1.225185332
A630082K20RIK	-1.151089491	LOC674707	1.108232523	ATP4A	1.22603486
D930044O18RIK	-1.151089491	TNIK	1.108302225	HBB-B1	1.22603486
PRSS33	-1.151089491	MCEE	1.108344537	NPNT	1.22603486
PRKAR1B	-1.151089491	LOC277881	1.108800644	EFNB2	1.22603486
FCNA	-1.151089491	RECQL4	1.108800644	NTRK2	1.226884977
GRASP	-1.151083902	CHDH	1.108800644	STARD10	1.226884977
NET1	-1.151049036	3110040N11RIK	1.108800644	CACNG2	1.227735684
FGF9	-1.150291893	NAP1L1	1.108800644	2310057H16RIK	1.229438867
UGT1A6	-1.150291893	LOC381114	1.108800644	1110065P19RIK	1.229438867
2310047N01RIK	-1.150291893	BC061259	1.108800644	LPL	1.229438867
D6ERTD365E	-1.150291893	UBE2G1	1.108800644	LOC626309	1.230041568
2310026J01RIK	-1.150291893	1810009K13RIK	1.108800644	COX8B	1.230291345
0910001K20RIK	-1.149494848	ELL3	1.108800644	SRPX	1.230291345
PDLIM7	-1.149494848	5330431N19RIK	1.109054482	COL8A2	1.230291345
SARS1	-1.149494848	HINT3	1.109384761	A430065P19RIK	1.231144413
ALPI	-1.149494848	ALDH3A1	1.109502673	CCL21A	1.231144413
4930573I19RIK	-1.149494848	1190002A17RIK	1.109526451	COL8A1	1.231998073
DAPK2	-1.149047521	GLRA2	1.109562199	CAR3	1.232107159
SEMA4F	-1.148927075	2310016A09RIK	1.109569472	SFRS5	1.233047853
CRELD2	-1.148776028	NUP50	1.109569472	PDCD7	1.23370717
TNFRSF21	-1.148753452	PPP6C	1.109569472	OLFML2B	1.23370717
C230072K23	-1.148698355	AW550801	1.109569472	PLUNC	1.234562607
AA960558	-1.148698355	1200003I07RIK	1.109569472	PLAC9	1.234562607
STX8	-1.148698355	E030003N15RIK	1.109569472	CTSH	1.234562607
LOC381758	-1.148698355	HRBL	1.109569472	4933425L03RIK	1.235418637
RBMX	-1.148063761	SEC61G	1.109569472	4930457P18RIK	1.236275261
B230387C07RIK	-1.147902414	ZNHIT4	1.109569472	2310040A07RIK	1.236275261
ARAF	-1.147902414	LOC380665	1.109569472	TMEM130	1.237603129
PNKD	-1.147902414	PQLC3	1.109674762	PIK3R1	1.237990291
5730439E10RIK	-1.147902414	AQP1	1.11033365	LOC100046259	1.238820789
2210420J11RIK	-1.147902414	ACTG2	1.110338834	SYNGR2	1.238848698
TULP4	-1.147899613	NFI-X3	1.110338834	COL14A1	1.238848698

D030056L22RIK	-1.147672063
CEP120	-1.14726729
TRIM36	-1.147245668
LOC381217	-1.147107024
TRIM37	-1.147107024
PLA2G12A	-1.147107024
RTN1	-1.147107024
GRPR	-1.147107024
LOC547380	-1.146382655
SIDT1	-1.146312186
SDPR	-1.146312186
DYNC1I1	-1.146312186
IGH-6	-1.146312186
ALDH3A2	-1.146312186
LAMC1	-1.146025163
MMP15	-1.145972003
D130059O18RIK	-1.145517898
4732455O04RIK	-1.145517898
FBXO9	-1.145517898
DOCK10	-1.145517898
BC029169	-1.145517898
2510049J12RIK	-1.145517898
MEF2A	-1.145517898
BVES	-1.145517898
NCDN	-1.145517898
DMRTA1	-1.145517898
GRXCR2	-1.145336511
UGT1A9	-1.144724161
ADPRH	-1.144724161
1600029D21RIK	-1.144724161
BC037034	-1.144724161
ZWINT	-1.143930973
ELOVL6	-1.143930973
MPP7	-1.143930973
C430003P19RIK	-1.143930973
LOC278795	-1.143930973
DST	-1.143930973
DNER	-1.143930973
D230016N13RIK	-1.143138335
1810015C11RIK	-1.143138335
PTPRZ1	-1.143138335
SLC6A17	-1.143138335
2810025O06RIK	-1.143138335
COL15A1	-1.143138335
9930021D14RIK	-1.143138335

LSM4	1.110338834
DTYMK	1.110338834
5830454D03RIK	1.110338834
MATK	1.110338834
NFIX	1.110338834
NGP	1.110338834
ATG10	1.110338834
NMB	1.110338834
SUMF2	1.110832915
CFL1	1.111108729
GYLTL1B	1.111108729
ETFB	1.111108729
PTPRB	1.111108729
GJA7	1.111108729
1190002J23RIK	1.111108729
RHBDL3	1.111108729
MRPS12	1.111108729
LOC333830	1.111108729
MAP2K3	1.111108729
9430081H08RIK	1.111108729
PROM1	1.111108729
H2-Q5	1.111289387
NCRNA00117	1.111409157
IRX3	1.111659565
GABARAPL1	1.111735467
HMGCL	1.111815475
KCNJ16	1.111879158
LRRC8A	1.111879158
HINT1	1.111879158
1810008A14RIK	1.111879158
CD53	1.111879158
SOX17	1.111879158
GNGT2	1.111879158
1110049G11RIK	1.111879158
4631426J05RIK	1.111879158
KRT1-15	1.111879158
G630023A01RIK	1.111879158
MT-ND1	1.111902631
IGF2BP3	1.112059788
APPBP2	1.112348916
IGFBP7	1.11256515
PPIL1	1.112650121
LOC386288	1.112650121
1110017L21RIK	1.112650121
NCF4	1.112650121

FARP1	1.2397077
ITGA11	1.240567298
FAIM2	1.240580651
GGA2	1.242288282
CCL21C	1.242288282
AA175286	1.242288282
NOXA1	1.243149669
CAMK2N1	1.243515539
OGN	1.243728771
IMPACT	1.243972569
COL1A2	1.244011653
8430436L14RIK	1.244874235
3300001A09RIK	1.244874235
PDGFRL	1.245737416
COL12A1	1.247465572
NEDD4L	1.248330549
2610528J11RIK	1.248330549
MRC2	1.248330549
GSTA2	1.249196126
D830014E11RIK	1.250062303
PTPLB	1.250062303
1200006O19RIK	1.25092908
SEMA3D	1.25092908
VAMP3	1.251796459
SYTL1	1.252664439
MORF4L1	1.252664439
E2F2	1.25353302
MORF4L2	1.254402205
FADD	1.255271991
MLF1	1.255271991
G0S2	1.256142381
IFT172	1.257013375
4931426K16RIK	1.257884972
SLC27A3	1.258757174
SLC35A1	1.258757174
2610019E17RIK	1.25962998
AI429486	1.25962998
ANXA1	1.262252032
DLX3	1.262252032
NDPH	1.262252032
CALCA	1.264003098
DLK2	1.265019749
FXYD3	1.265756594
1700023F06RIK	1.265756594
DNASE1	1.266634254

GFER	-1.142697551	2410016F19RIK	1.112650121	1700016K19RIK	1.26698126
ACLY	-1.142346247	CYHR1	1.112906747	COL24A1	1.270150983
3110030K17RIK	-1.142346247	POFUT2	1.113421618	AI428936	1.271913007
ADAMTS1	-1.142346247	PCID1	1.113421618	APOC1	1.272794935
WFDC2	-1.142226487	OTOA	1.113421618	E130304D01	1.272794935
BCL2L11	-1.142143595	ICRFP703B1614Q5.6	1.113421618	PLCD1	1.273677475
LOC638935	-1.142117803	3110023F10RIK	1.113421618	MATN4	1.274560627
COL4A1	-1.141919449	2410004P03RIK	1.113421618	FBP2	1.274560627
GSTM7	-1.141856585	LRRC1	1.113421618	STAC	1.275444392
TRHR	-1.141554707	2510048K03RIK	1.113421618	KRT2-7	1.278099363
CLCN3	-1.141554707	PTPRV	1.113421618	LOC381241	1.278099363
EBF1	-1.141554707	COPE	1.113421618	LTBP2	1.278985581
LAPTM5	-1.140923244	1700008B15RIK	1.113421618	CNNM2	1.282536603
SDK1	-1.140763716	D930007N19RIK	1.113421618	6330404C01RIK	1.284315809
LGALS3BP	-1.140763716	SLC7A3	1.113435656	STFA2	1.285206337
B430216N15RIK	-1.140763716	ACOT1	1.113520853	MYL6	1.285206337
KIF3C	-1.140763716	CDC42BPB	1.113522424	LHX3	1.286989247
GATS	-1.140763716	ZFP219	1.113555632	EFNA5	1.28788163
2810451E09RIK	-1.140763716	GNAI1	1.113810407	2010005H15RIK	1.28877463
4632413K17RIK	-1.140763716	IGFBP3	1.114090885	BMP4	1.28877463
4632415K11RIK	-1.140763716	PLCE1	1.114193651	C920027I18RIK	1.29145735
1190009E20RIK	-1.140395511	LOC212539	1.114193651	SLC25A1	1.291848632
6332401O19RIK	-1.139973273	BICC1	1.114193651	SLC39A1	1.292352831
1810054O13RIK	-1.139973273	HIST1H2AH	1.114193651	CLEC11A	1.292352831
MAPK8IP	-1.139973273	1110003P22RIK	1.114193651	BRIP1	1.293248932
NUCB1	-1.139973273	RFX2	1.114193651	1700010A17RIK	1.293248932
C230026C11	-1.139973273	TST	1.114193651	ADRA2A	1.293248932
IL10RB	-1.139973273	1700022C02RIK	1.114193651	IFITM5	1.296839555
1110001E17RIK	-1.139973273	TCIRG1	1.114193651	BC023928	1.299539062
1810043M15RIK	-1.139973273	2810012D02RIK	1.114193651	SSTR2	1.299539062
SLC35F3	-1.139973273	RPS24	1.114193651	LOC272465	1.303147149
B230386D16RIK	-1.139183377	ODZ3	1.114193651	A630086H07RIK	1.304050735
RHOT1	-1.139183377	UBL4A	1.114193651	EG433016	1.305328449
OSBPL1A	-1.139183377	LOC383802	1.114193651	KCNE1	1.309462895
FGFR3	-1.139183377	HTR3A	1.114597673	THBS2	1.310393404
PADI2	-1.139183377	RAB36	1.114772689	2510042H12RIK	1.311302014
MTAP4	-1.139183377	MMD	1.114811022	5730410E15RIK	1.315854525
PFKL	-1.138991585	RPL22	1.114950881	ARF2	1.315854525
LOC233637	-1.138785985	ETS2	1.114966219	CAPN6	1.318593614
BC023892	-1.138463167	CDKN3	1.114966219	DFNB31	1.319507911
PKD2L1	-1.138402988	RAP2C	1.114966219	CACNA2D2	1.319507911
NRXN1	-1.138394029	ST7	1.114966219	PHEX	1.320422841
FREQ	-1.138394029	HBB-B2	1.114966219	4930449E07RIK	1.322254605
ACCN2	-1.138394029	HMGN3	1.114966219	MSLNL	1.323289247
9030224M15RIK	-1.138394029	IGF2R	1.114966219	MGP	1.32408891

NKD1	-1.138394029
TSPAN2	-1.137605228
1600010D10RIK	-1.137605228
TGFBR3	-1.137605228
MID1IP1	-1.137605228
ZFP521	-1.13754978
DDOST	-1.137509701
PDIA4	-1.137406351
TMEM2	-1.136816973
6330505N24RIK	-1.136816973
1700061J05RIK	-1.136816973
SORL1	-1.136787118
LOC100044322	-1.136636085
CRABP1	-1.136557026
UAP1L1	-1.136029265
GARS	-1.136029265
E130201N16RIK	-1.136029265
A730075L14RIK	-1.136029265
SLC6A13	-1.135242102
6230403H02RIK	-1.135242102
NR1H4	-1.135242102
MAPKBP1	-1.135242102
C130065N10RIK	-1.135242102
STC1	-1.134455485
D630014A15RIK	-1.134455485
CNOT4	-1.134455485
LOC384349	-1.134455485
ISL1	-1.134455485
AI854408	-1.134455485
OTOP1	-1.134455485
3110006E14RIK	-1.134455485
D130066H20RIK	-1.134455485
5730467H21RIK	-1.134455485
BCL2L2	-1.134455485
2610036D13RIK	-1.133888264
CLGN	-1.133669413
2700083E18RIK	-1.133669413
RASL10B	-1.133669413
RNF14	-1.133669413
MDM2	-1.133669413
ABCA5	-1.133669413
4930506D23RIK	-1.133669413
SCARA3	-1.133669413
FAM132A	-1.133578021
D7ERTD715E	-1.132883885

EVPL	1.114966219
MMRN2	1.114966219
LOC100039751	1.115068765
CD44	1.115313973
OTX1	1.115408334
WDR24	1.115473615
SLC16A5	1.11555511
NME2	1.115739322
AB041544	1.115739322
LOC382628	1.115739322
MAK	1.115739322
IL17RB	1.115739322
CSF1R	1.115739322
BC046418	1.115739322
LOC380906	1.115739322
4930422J18RIK	1.115739322
PLCG2	1.115739322
2310057G13RIK	1.115739322
PFDN5	1.11586712
BLCAP	1.11587469
SAR1A	1.116512962
AI481316	1.116512962
FBL	1.116512962
3300001G02RIK	1.116512962
TYMS	1.116512962
COL16A1	1.116512962
HBA-A1	1.116512962
LOC231663	1.116512962
LUZP2	1.116512962
LOC380980	1.116512962
LOC194642	1.116512962
SPINK5	1.116512962
RASGRP3	1.116512962
CPA3	1.116512962
CEPT1	1.116512962
DDX21	1.116512962
7630402G21RIK	1.116512962
5133400G04RIK	1.116725134
LOC100045312	1.116728087
SNIP1	1.117287138
BCL2A1D	1.117287138
SLCO2A1	1.117287138
1810007P19RIK	1.117287138
GALGT1	1.117287138
TRIB3	1.117287138

STFA3	1.325007017
9130024F11RIK	1.32592576
VDR	1.32592576
0610030P10RIK	1.326845141
AI586120	1.326845141
4CYTL1	1.328395297
SP7	1.329607108
PKHD1L1	1.329607108
SLC38A5	1.332374825
GRP	1.337000495
MDH1B	1.339783602
ALCAM	1.340712592
SLC12A8	1.343503426
FAM148C	1.344701033
IGSF1	1.346300069
0610041G09RIK	1.347233577
NUPR1	1.347233577
CLDN10	1.349102534
LOC238726	1.349102534
2410008K03RIK	1.350037985
GLRX	1.351355319
CYP11A1	1.353786279
LYPD2	1.354548992
HSPA2	1.360059264
TTR	1.360384936
FBXW5	1.361314116
LOC385068	1.361353927
DIO2	1.363202607
TCEAL5	1.364147835
0610041D19RIK	1.365093718
E030030K01RIK	1.366621788
C80638	1.366987452
HES6	1.368017121
PPIL6	1.36983298
KY	1.36983298
ENPP6	1.36983298
REC8L1	1.372684431
CHRNA10	1.379892635
GLRX1	1.384149716
AKP2	1.384149716
BMPER	1.387030969
C230047J02RIK	1.387992719
4932425I24RIK	1.388955136
NPPA	1.395615904
CILP2	1.395710764

BBOX1	-1.132883885
TRIP12	-1.132883885
MAGEE1	-1.132883885
DHX33	-1.132883885
SEMA3A	-1.132098902
A830004L04RIK	-1.132098902
RAB15	-1.132098902
TEX2	-1.132098902
MYLPF	-1.132098902
FBXO32	-1.132098902
DARC	-1.132098902
SLC7A4	-1.132083196
MT3	-1.131795506
FKBP9	-1.13154031
NPB	-1.131314463
NFE2L3	-1.131314463
EVL	-1.131314463
PTTG1IP	-1.131314463
TEF	-1.131314463
LOC384158	-1.131314463
LOC384790	-1.131314463
EPB4.1L3	-1.131314463
6430543G08RIK	-1.131314463
CXCR4	-1.131314463
WHRN	-1.131177278
IL33	-1.131051017
ANKRD22	-1.130530567
CCKBR	-1.130530567
6330548O06RIK	-1.130530567
PRSS35	-1.129961129
NPTX2	-1.12978424
PKM2	-1.129754355
2810006K23RIK	-1.129747215
FDPS	-1.129747215
FLRT3	-1.129747215
C030036D22RIK	-1.129747215
OGT	-1.129747215
CTNS	-1.129747215
HS3ST2	-1.129747215
LOC216223	-1.129747215
6030498E09RIK	-1.129747215
1300010O06RIK	-1.129747215
FKTN	-1.129744778
ACTB	-1.129063576
RASSF4	-1.128964405

IL11RA1	1.117287138
6330571D19RIK	1.117287138
LOC381000	1.117287138
EIF3S3	1.117287138
GPM6B	1.117287138
2600003E23RIK	1.117287138
D3ERTD789E	1.117287138
AARD	1.117402997
F730003H07RIK	1.117615919
STMN2	1.117647374
FKBP14	1.118061851
1110011K10RIK	1.118061851
BMP5	1.118061851
AI894139	1.118061851
A930027K05RIK	1.118061851
BC030867	1.118061851
D17H6S56E-5	1.118061851
BCL2A1B	1.118061851
CD68	1.118061851
GTF2H1	1.118473444
SLCO3A1	1.118837101
RPL36	1.118837101
IRAK1	1.118837101
HOOK3	1.118837101
4930546H06RIK	1.118837101
PRKRIR	1.118837101
MSLN	1.118837101
PEG3	1.118844094
LOC668706	1.118958981
B230365C01RIK	1.119422751
RIPK3	1.119612889
SEC61B	1.119612889
TBRG1	1.119612889
NQO1	1.119612889
2310022B05RIK	1.119612889
6430559E15RIK	1.119612889
TTC18	1.119612889
FGFBP1	1.119612889
3110001A13RIK	1.119612889
A930003A15RIK	1.119821675
CITED1	1.119912412
PADI1	1.120389214
LOC382087	1.120389214
5730449L18RIK	1.120389214
NTN4	1.120389214

A330021E22RIK	1.396678532
MGC58818	1.397646972
TSGA2	1.397646972
AQP5	1.398023872
ZIC2	1.399641645
B3GNT8	1.399818665
MSX3	1.402499251
1700028N11RIK	1.41519416
5330432B20RIK	1.416175438
SDC2	1.418140036
2310067L16RIK	1.419123356
1110019O10RIK	1.425037614
GJA1	1.431968741
LOC332309	1.433955248
IL5	1.436940177
DCT	1.457432321
OMD	1.464085696
TMEM91	1.467423939
ESRRB	1.474684317
1110017I16RIK	1.475291457
PKN3	1.475291457
ST8SIA3	1.478362431
KCNE4	1.491744027
PTGIS	1.501079098
1700001C02RIK	1.505246747
1700007G11RIK	1.531557997
MFNG	1.546492675
IBSP	1.556075344
1700019F09RIK	1.556170353
PLOD2	1.556170353
D11BWG0517E	1.571345033
BC064033	1.581178233
VIT	1.592176198
A230005G17RIK	1.604362333
1700024G13RIK	1.611048582
SPP1	1.616641738
RN18S	1.617122685
CTSK	1.617762697
COL1A1	1.631274987
MOGAT1	1.633537982
LOC673501	1.634516365
SLC25A19	1.634670657
E130113E03RIK	1.636938363
PRB3	1.643760375
4732467B22	1.656341323

B430201A12RIK	-1.128964405
SMYD2	-1.128964405
3110054C06RIK	-1.128964405
TRIOBP	-1.128964405
6430515G22RIK	-1.128964405
ADPRHL1	-1.128964405
1500031H04RIK	-1.128964405
0610012G03RIK	-1.128964405
CATNA2	-1.128964405
C630025L14	-1.128964405
SLC7A14	-1.128642614
FADS3	-1.128182137
1200006F02RIK	-1.128182137
D8ERTD587E	-1.128182137
LOC384646	-1.128182137
STRC	-1.128182137
SGK3	-1.128182137
GABARAPL2	-1.127400412
5330414D10RIK	-1.127400412
LLGLH2	-1.127400412
CYCS	-1.127400412
LOC224532	-1.127400412
EPN2	-1.127400412
BZRAP1	-1.127400412
ATXN1L	-1.127137084
4922501L14RIK	-1.126708833
NOMO1	-1.126668842
WIF1	-1.126619228
8030499H02RIK	-1.126619228
MAT2A	-1.126619228
AP1GBP1	-1.126619228
AV028368	-1.126619228
1110003F05RIK	-1.126619228
ATP6V1E1	-1.126619228
CCNDBP1	-1.126619228
0610040J01RIK	-1.126619228

1110055O21RIK	1.120389214
VAT1	1.120389214
ANLN	1.120389214
GNG12	1.120389214
2010315L10RIK	1.120389214
PDCD4	1.120389214
1200015P04RIK	1.120389214
E030026I10RIK	1.120389214
MAN1C1	1.120389214
FLI1	1.120389214
2410015N17RIK	1.120389214
MFI2	1.120434932
TMIE	1.120745494
POLR2I	1.121166078
4930402H24RIK	1.121166078
CCT4	1.121166078
LOC381298	1.121166078
LOC219145	1.121166078
FBN1	1.121166078
EEF1B2	1.121183993
1500004F05RIK	1.121321166
DAAM1	1.121751694
4933428I03RIK	1.121943481
2610318I18RIK	1.121943481
MYLC2B	1.121943481
2300009N04RIK	1.121943481
NUDC	1.121943481
LOC381250	1.121943481
TCF7L2	1.121943481
EML1	1.121943481
GSTP1	1.121943481
LOC234640	1.121943481
SURF4	1.121943481
LOC384179	1.122721422
KRTCAP2	1.122721422

RASD2	1.659789171
LOC381375	1.659789171
PSAT1	1.662091723
1700027A23RIK	1.663244197
PACRG	1.665551542
OTOF	1.680114181
POU4F3	1.683127656
CRLF1	1.692317193
MMP9	1.708819482
D1ERTD471E	1.714752073
GOLPH2	1.73748437
CA3	1.758079122
LOC217071	1.788809804
BGLAP-RS1	1.812523877
1700009P17RIK	1.818816504
ANXA4	1.903977782
BGLAP2	1.922521857
STFA1	1.923854909
MNS1	1.931872658
BGLAP1	1.984808749
GPX2	1.990621188
DDX3Y	2.03290872
S100A8	2.042024251
3100002J23RIK	2.056227653
EGFL9	2.188587403
2310046K01RIK	2.411615655
IGL-5	2.507698682
2010001J22RIK	2.509726185
SCG2	2.602683711
MORN5	2.723685528
4930443F05RIK	2.732080514
DYNLRB2	3.12074543
MEIG1	3.408656087
1110017D15RIK	4.0278222
MYO3A	4.3283585

Table S7. Primers used for RTPCR (a) and Taqman primer/probe sets (b) used for qRTPCR..

**a) cDNA primers for RTPCR**		
**Gene**	**cDNA Primer F**	**cDNA Primer R**
Tmem97	CTGCCGCCCGAACTATACC	TAGATGATTGCAGGGATTCG
Gnb4	TTACAAGTTCGGGAGACACG	CATGTGGCATCATCAGAACC
Mpv17l	GGGAAGGATGACATATTTTTGG	TGGACTGCAGTGTACCATCG
Clvs1	GGAAGATTCTCTTGCTGTTTGC	GGAACTCGGGGTGTATTAGC
Sox5	CAGCAACACCAGGCTTAGG	ACTGCTGAGTCAGGCTCTCC
Ankrd27	GCAGACCACGAGGGTTACG	TGACAGGTCACTCTGCTTTCC
Zfp251	GAGACGCCTCTGACCTTCC	AAAATGGATAGCTCCTTCTCG
Cacna1c	GAGCCACGGTGAATCAGG	GGGAATGTGGTAGGAGAATGG
Akap7	AGGTTCCCTGACAGACATGC	TTGTTGGTGATCGGAATGG
Unkl	GCTTCCTCAAGTGCAAGTCC	GGCTTCCACCTCTTCTTTCC
6820408C15Rik	CGCAACAAAAGCAAGACG	TTGAGCTTGCTGAGGTAGCC

**b) Taqman primer/probe sets for qPCR**	
**Gene**	**qPCR probe**
Chrna1	4331348 (manual design using Applied Biosystems software)
Bhlhe40	4331348 (manual design using Applied Biosystems software)
Homer1	4331348 (manual design using Applied Biosystems software)
Gtf2e2	4331348 (manual design using Applied Biosystems software)
Rasd2	4331348 (manual design using Applied Biosystems software)
1700010A17Rik	4331348 (manual design using Applied Biosystems software)
2010001J22Rik	4331348 (manual design using Applied Biosystems software)
Dynlrb2	4331348 (manual design using Applied Biosystems software)
Meig1	4331348 (manual design using Applied Biosystems software)
Myo3a	4331348 (manual design using Applied Biosystems software)
MiTF	4331348 (manual design using Applied Biosystems software)
Zic2	4331348 (manual design using Applied Biosystems software)
Pou4f3	4331348 (manual design using Applied Biosystems software)
Kcnma1	Mm01268579_m1
Kcnq4	Mm01185500_m1
Kcnn2	Mm01266371_m1
Foxo1	Mm00490672_m1
Foxo3	Mm01185722_m1
Nr3c1	Mm01260500_m1
Htt	Mm01213820_m1
Fos	Mm01302932_g1
Ets1	Mm00468970_m1
Sp1	Mm03053855_g1
Agt	Mm00599662_m1
Trp53	Mm01337166_mH
Thrb	Mm01316714_m1

Myc	Mm00487804_m1
Tnc	Mm00495681_m1
Bmp4	Mm00432087_m1
Rhoa	Mm00834507_g1
Tgfb1	Mm00441729_g1,
Hprt1	Mm01318747_g1
Jag1	Mm01270190_m1
Ngfr	Mm00446296_m1
Ablim1	Mm01254316_m1
Tmem97	Mm01608791_g1
Gnb4	Mm00501973_m1
Mpv17l	Mm01170600_g1
Clvs1	Mm01217360_m1
Sox5	Mm00488386_m1
Ankrd27	Mm01254044_m1
Zfp251	Mm02342310_m1
Cacna1c	Mm00437946_m1
Akap7	Mm02581034_m1
Unkl	Mm00840627_m1

References

62 Chen, H. P. *et al.* Intrathecal miR-96 inhibits Nav1.3 expression and alleviates neuropathic pain in rat following chronic construction injury. *Neurochem Res* **39**, 76-83, (2014).

63 Gu, C. *et al.* MiR-183 family regulates chloride intracellular channel 5 expression in inner ear hair cells. *Toxicol In Vitro* **27**, 486-491, (2013).

64 Henson, B. J. *et al.* Transcriptional and post-transcriptional regulation of SPAST, the gene most frequently mutated in hereditary spastic paraplegia. *PLoS One* **7**, e36505, (2012).

65 Jalvy-Delvaille, S. *et al.* Molecular basis of differential target regulation by miR-96 and miR-182: the Glypican-3 as a model. *Nucleic Acids Res* **40**, 1356-1365, (2012).

66 Jeon, T. I. *et al.* An SREBP-responsive microRNA operon contributes to a regulatory loop for intracellular lipid homeostasis. *Cell Metab* **18**, 51-61, (2013).

67 Jeong, H. J., Park, S. Y., Yang, W. M. & Lee, W. The induction of miR-96 by mitochondrial dysfunction causes impaired glycogen synthesis through translational repression of IRS-1 in SK-Hep1 cells. *Biochem Biophys Res Commun* **434**, 503-508, (2013).

68 Krol, J. *et al.* Characterizing light-regulated retinal microRNAs reveals rapid turnover as a common property of neuronal microRNAs. *Cell* **141**, 618-631, (2010).

69 Li, C. *et al.* GPC1 regulated by miR-96-5p, rather than miR-182-5p, in inhibition of pancreatic carcinoma cell proliferation. *Int J Mol Sci* **15**, 6314-6327, (2014).

70 Vishwamitra, D. *et al.* MicroRNA 96 is a post-transcriptional suppressor of anaplastic lymphoma kinase expression. *Am J Pathol* **180**, 1772-1780, (2012).

71 Wang, L. *et al.* MicroRNAs 185, 96, and 223 repress selective high-density lipoprotein cholesterol uptake through posttranscriptional inhibition. *Mol Cell Biol* **33**, 1956-1964, (2013).

72 Wang, Y., Huang, J. W., Calses, P., Kemp, C. J. & Taniguchi, T. MiR-96 downregulates REV1 and RAD51 to promote cellular sensitivity to cisplatin and PARP inhibition. *Cancer Res* **72**, 4037-4046, (2012).

73 Yan, Z. *et al.* miR-96/HBP1/Wnt/beta-catenin regulatory circuitry promotes glioma growth. *FEBS Lett* **588**, 3038-3046, (2014).

74 Zhang, J. *et al.* miR-96 promotes tumor proliferation and invasion by targeting RECK in breast cancer. *Oncol Rep* **31**, 1357-1363, (2014).
